# Novel 1,2,4-oxadiazole derivatives as selective butyrylcholinesterase inhibitors: Design, synthesis and biological evaluation

**DOI:** 10.17179/excli2021-3569

**Published:** 2021-05-18

**Authors:** Maryam Nazari, Elham Rezaee, Roshanak Hariri, Tahmineh Akbarzadeh, Sayyed Abbas Tabatabai

**Affiliations:** 1 Department of Pharmaceutical Chemistry, School of Pharmacy, Shahid Beheshti University of Medical Sciences, Tehran, Iran; 2 Department of Medicinal Chemistry, Faculty of Pharmacy, Tehran University of Medical Sciences, Tehran, Iran

**Keywords:** Alzheimer's disease, 1,2,4-oxadiazole, biological evaluation, butyrylcholinesterase inhibitor, synthesis

## Abstract

Alzheimer's disease (AD) is a progressive mental disorder that brings a huge economic burden to the healthcare systems. During this illness, acetylcholine levels in the cholinergic systems gradually diminish, which results in severe memory loss and cognitive impairments. Moreover, Butyrylcholinesterase (BuChE) enzyme participates in cholinergic neurotransmission regulation by playing a prominent role in the latter phase of AD. In this study, based on donepezil, which is an effective acetylcholinesterase (AChE) inhibitor, a series of 1,2,4-oxadiazole compounds were designed, synthesized and their inhibitory activities towards AChE and BuChE enzymes were evaluated. Some structures exhibited a higher selectivity rate towards BuChE in comparison to donepezil. Notably, compound **6n** with an IC_50_ value of 5.07 µM and an SI ratio greater than 19.72 showed the highest potency and selectivity towards BuChE enzyme. The docking result revealed that compound **6n** properly fitted the active site pocket of BuChE enzyme, and formed desirable lipophilic interactions and hydrogen bonds. Moreover, according to *in silico* ADME studies, these compounds have proper potential for being developed as new oral anti-Alzheimer's agents (Figure 1(Fig. 1)).

## 1 Introduction

Alzheimer's disease (AD) is a progressive illness characterized by loss of memory, the reduced thinking and cognition abilities, psychiatric disorders like depression, and difficulties in performing daily activities (Sugimoto et al., 2012[27]; Alzheimer's Associaton, 2013[1]; Soria Lopez et al., 2019[26]; Sengoku, 2020[24]). According to the World Alzheimer's Report in 2019, 50 million people suffer from dementia worldwide, and this number is estimated to increase up to more than 152 million by 2050 (Alzheimer's Disease International 2019[3]). Accordingly, this fatal multifactorial disease is very burdensome due to its illness duration before death (Alzheimer's Association 2017[2]; Cass 2017[7]; Femminella et al., 2018[14]). The complex pathological hallmarks of AD seem to result from chronic oxidative stress, mitochondrial dysfunction, extracellular beta-amyloid (Aβ) production, and intra-neuronal neurofibrillary tau tangles accumulation (Oboudiyat et al., 2013[21]; Anand et al., 2014[5]; Busche and Hyman, 2020[6]; Vaz and Silvestre, 2020[29]). In addition, AD is accompanied with the deterioration of the cholinergic system (Hampel et al., 2018[16]; Sharma, 2019[25]). Acetylcholine (ACh) is one of the substantial neurotransmitters in particular brain synapses, deficiency of which would lead to cognitive impairment of the disease (Amenta and Tayebati, 2008[4]; Richter et al., 2018[22]; Hampel et al., 2019[17]). There are two main cholinesterase enzymes (ChEs) throughout the body belonging to the α/β-hydrolase family, which were found to be responsible for the regulation of cholinergic neurotransmission. Acetylcholinesterase (AChE) is mostly found in neuronal cells, the primary function of which is the degradation of ACh. While butyrylcholinesterase (BuChE), also named as nonspecific or pseudocholinesterase, is mainly produced by glial cells, so it is liable for the hydrolysis of choline-based esters (Franjesevic et al., 2019[15]). The structures of both ChEs were found to be very similar as ChEs sequence comparison exhibited a similarity of 66 % (Sawatzky et al., 2016[23]). In this regard, the AChE enzyme has a greater tendency to small molecules like ACh, but BuChE provides a wider space for larger substrates (Franjesevic et al., 2019[15]).

**Figure 2:**
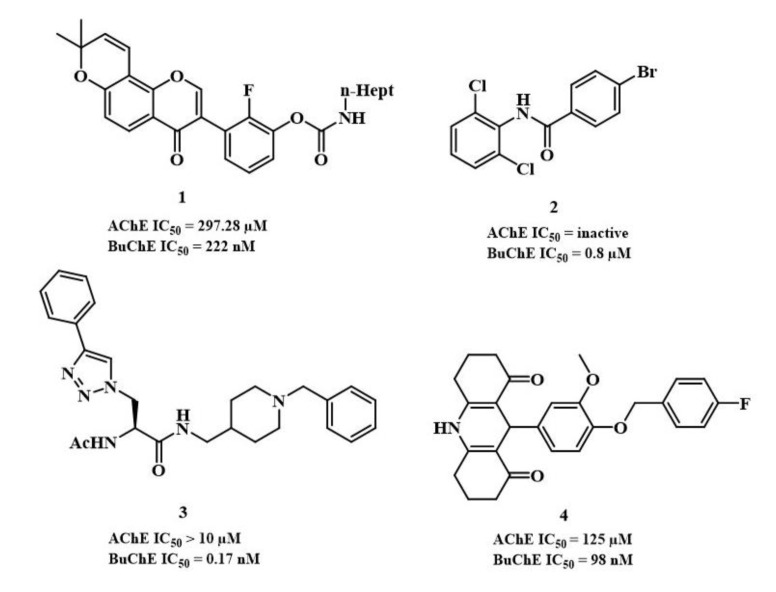
Chemical structures of highly selective BuChE inhibitors

Based on the prominent role of BuChE at the late stages of AD pathogenesis (Darvesh et al., 2003[10]), and small side effects of BuChE inhibitors, targeting BuChE might be a promising strategy for the control of disease progression. Therefore, recent attempts have been made to design selective BuChE inhibitors (Figure 2(Fig. 2)) (Li et al., 2017[19]). As shown in Figure 2(Fig. 2), compound **1** is a carbamate-based derivative and compound **2** is a non-cytotoxic benzamide with a high selectivity and inhibitory activity towards BuChE enzyme (Wajid et al., 2019[30]; Wu et al., 2019[31]). Compounds **3** and **4**, which were designed based on donepezil and tacrine, demonstrated a high selectivity against BuChE enzyme, respectively (de Andrade et al., 2019[11]; Joubert and Kapp, 2020[18]). Furthermore, previous oxadiazole derivatives synthesized as the selective BuChE showed no significant inhibitory activity and selectivity against this enzyme (Zhang et al., 2019[33]).

**Figure 3:**
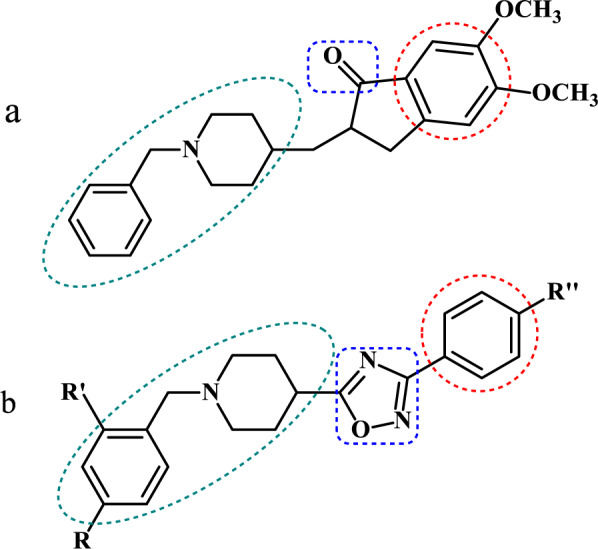
The corresponding parts of donepezil (a) to the designed compounds (b) are shown.

Donepezil, a selective AChE inhibitor with a low anti-BuChE effect, is currently administered as an anti-AD medication due to possessing fewer side effects and longer half-life. Moreover, it has been considered as an appealing lead compound for designing new agents (van Greunen et al., 2017[28]). However, this medicine could only relieve the symptoms of the disease, and it is incapable of being effective on moderate to severe AD (Coman and Nemeş, 2017[8]). Accordingly, the starting point of our investigation was donepezil in order to design a structure with the improved inhibitory effects on BuChE that might be promising in the latter phase of the disease. Therefore, we designed the novel donepezil-like compounds by maintaining the *N*-benzylpiperidine moiety and replacing the indanone segment with a 1,2,4-oxadiazole ring that was connected to an aromatic ring with different substituents (Figure 3(Fig. 3)). In the current study, synthesis, biological evaluation, docking study, and ADME prediction were reported. 

## 2 Results and Discussion

### 2.1 Chemistry

**Figure 4:**
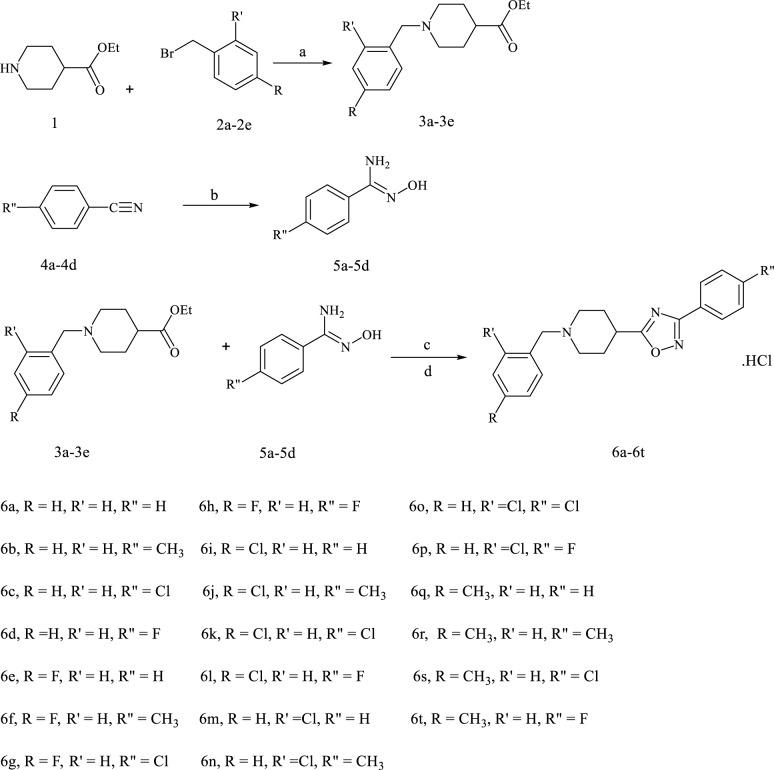
Reagents and conditions: (a) K_2_CO_3_, DMF, r.t, 48 h, stir, 59.7 %-76.6 %; (b) Hydroxylammonium chloride, Na_2_CO_3_, H_2_O, EtOH, 24 h, reflux, 68.2 %-96.5 %; (c) NaOEt, dry EtOH, 24 h, reflux, (d) HCl in diethyl ether, 20.9 %-56.3 %.

According to Figure 4(Fig. 4), the commercially available starting material **1** and para- or ortho-substituted benzyl bromides **2** were mixed for 48 hours in the presence of potassium carbonate in order to afford various N-benzylated intermediate **3**. A mixture containing benzonitrile **4**, hydroxylamine hydrochloride and sodium carbonate in ethanol/H_2_O was refluxed for 24 hours to achieve para-substituted amidoximes **5**. The 1, 2, 4-oxadiazole ring was closed by the reaction of intermediate **3** with several amidoximes **5** in the presence of sodium ethoxide. Finally, by the addition of hydrochloric acid in diethyl ether, compounds **6a-6t** were achieved (Zavareh et al., 2014[32]).

### 2.2 Inhibitory activity against AChE and BuChE enzymes

The inhibitory activity of the designed compounds against BuChE and AChE was evaluated using Ellman's method. During this process, donepezil was considered as a standard compound. As summarized in Table **1**(Tab. 1), some compounds in this series depicted proper inhibitory activities against BuChE with desirable IC_50_ values (5.07 µM to 81.16 µM), and also exhibited more selectivity towards BuChE, rather than AChE, in comparison with donepezil. Additionally, compounds **6n **and** 6b** with a methyl group in the Rʺ position were found to be the most potent ones for BuChE with IC_50_ values of 5.07 µM and 9.81 µM and high selectivity with SI ratios greater than 19.72 and 10.19, respectively. However, compound **6a** with no substituent in this position showed moderate potency against BuChE (IC_50_= 14.23 µM) and AChE (IC_50_= 35.46 µM).

Apparently, substitution in the R and Rʹ positions would enhance inhibitory potency in the order of 2-chloro > 4-chloro > 4-fluoro, respectively, indicating that the large lipophilic electron-withdrawing groups in these places would be in favor of the anti-BuChE activity.

### 2.3 Docking study

**Figure 5:**
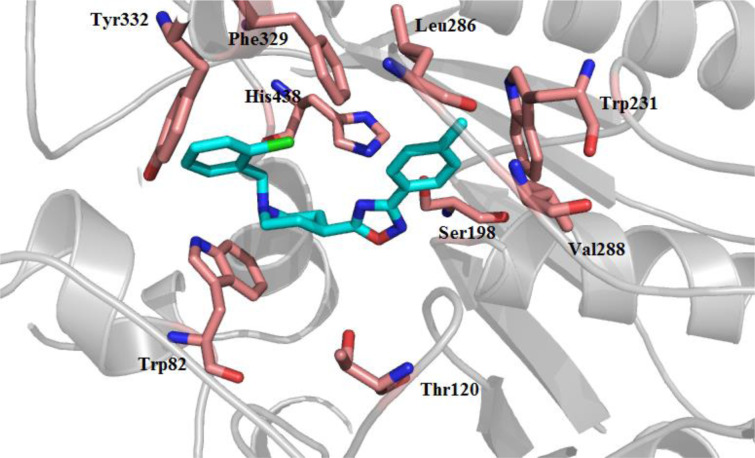
Compound 6n (light blue sticks) in the catalytic pocket of BuChE (PDB: 1P0I). A hydrogen bond was observed between nitrogen of the pyridine ring and Trp82. The 4-methyl benzene moiety of 6n was located in the acyl pocket of the enzyme via lipophilic interactions. Hydrogen bonds could form between oxadiazole ring with His438, Ser198 and Thr120. Moreover, the benzyl ring had hydrophobic interactions with Phe329 and Tyr332.

The molecular docking study was conducted using AutoDock Tools software version 1.5.6rc3. In this regard, the X-ray crystallographic structure of the human BuChE (PDB Code 1P0I) was utilized as an enzyme structure. The docked binding mode was analyzed for the interactions between compound **6n** and BuChE. As shown in Figure 5(Fig. 5)**,** the compound **6n** was well accommodated inside the gorge active site and adopted a U-shaped conformation. A hydrogen bond was also observed between the nitrogen of the pyridine ring and Trp82. The 4-methyl phenyl moiety of **6n** was located in the enzyme's acyl pocket via forming lipophilic interactions with residues of Leu286, Trp231, and Val288. Subsequently, the 1,2,4-oxadiazole ring could form hydrogen bonds with His438, Ser198, and Thr120. Moreover, the benzyl ring might have lipophilic interactions with Phe329, Trp 82 and Tyr332.

### 2.4 ADME properties

To predict the* in silico* ADME features of our proposed novel compounds, an online Swissadme calculator was used. According to Table **2**(Tab. 2), all the structures abided by the Lipinski's rule of five criteria (Ertl et al., 2000[13]), and the percentage absorption (%ABS) was predicted at 94.45 %. Accordingly, it seems that, these compounds might be orally bioavailable agents, which is known as a favorable route of drug administration.

See also the Supplementary information.

## 3 Conclusion

In this study, a series of 1,2,4-oxadiazole derivatives, as novel selective inhibitors of BuChE, were rationally designed and synthesized. Thereafter, the inhibitory activities of the compounds against AChE and BuChE enzymes were evaluated. Based on our findings, some compounds exhibited high selectivity towards BuChE and the most potent compound was found to be **6n** (IC_50_ = 5.07 µM, SI ratio > 19.72) with chlorine and methyl group in Rʹ and Rʺ positions, respectively. This compound possessed an appropriate lipophilicity as well as hydrophobic interactions with the active site of the BuChE. Indeed, it seems that the presence of the methyl group in the Rʺ position promoted this selectivity. Additionally, *in silico* ADME prediction showed the exemplary oral bioavailability of these structures. In summary, the designed structures have the potential to act as promising starting points in order to develop more selective BuChE inhibitors with the improved pharmacokinetic properties for the treatment of Alzheimer's disease.

## 4 Experimental Section

### 4.1 Chemistry

All the reagents used in this study were achieved from Aldrich or Merck Company with no further purification. ^1^H NMR and ^13^C NMR spectra were afforded by a Bruker Avance II spectrophotometer using CDCl_3_, as a solvent, and tetramethylsilane, as an internal standard, at 400.20 and 100.64 MHz, respectively. Chemical shifts were reported in parts per millions (ppm). All mass spectra were obtained using HPLC Agilent 1100 spectrometer. Melting points were also taken using an Electrothermal 9100 apparatus and were not corrected afterward. A Perkin Elmer 834 spectrometer was utilized to record infrared spectra and the absorptions were expressed on the wave number (cm^-1^) scale ranged from 400 to 4000 cm^-1^. 

#### 4.1.1 General procedure for the synthesis of ethyl 1-benzylpiperidine-4-carboxylate derivatives (3a-e)

Ethyl piperidine-4-carboxylate **1** (1 equiv) was dissolved in 15 ml DMF and the mixture was then placed in a round bottom flask followed by the addition of K_2_CO_3_ (2 equiv) under stirring condition. Suitable benzyl bromide **2a-d** (1 equiv) was added dropwise while the flask was cooled in an ice bath. Thereafter, the ice bath was removed, and the mixture was stirred for 48 h at room temperature. Afterward, the medium was filtered, and the filtrate was extracted with water and diethyl ether. Finally, the organic layer was dried over MgSO_4_ and evaporated in vacuum in order to afford yellow oily liquid.

##### 4.1.1.1 ethyl 1-benzylpiperidine-4-carboxylate (3a) 

Yellow oily liquid; yield: 59.7 %; IR (KBr, cm^-1^): 1733 (C=O); LC-MS [M + 1]^+^: m/z 248.

##### 4.1.1.2 ethyl 1-(4-fluorobenzyl)piperidine-4-carboxylate (3b)

Yellow oily liquid; yield: 73.5 %; IR (KBr, cm^-1^): 1732 (C=O); LC-MS [M + 1]^+^: m/z 265.9.

##### 4.1.1.3 ethyl 1-(4-chlorobenzyl)piperidine-4-carboxylate (3c)

Yellow oily liquid; yield: 68.9 %; IR (KBr, cm^-1^): 1738 (C=O); LC-MS [M + 1]^+^: m/z 281.9.

##### 4.1.1.4 ethyl 1-(2-chlorobenzyl)piperidine-4-carboxylate (3d)

Yellow oily liquid; yield: 60.6 %; IR (KBr, cm^-1^): 1738 (C=O); LC-MS [M + 1]^+^: m/z 282.

##### 4.1.1.5 ethyl 1-(4-methylbenzyl)piperidine-4-carboxylate (3e)

Yellow oily liquid; yield: 76.6%; IR (KBr, cm^-1^): 1733 (C=O); LC-MS [M + 1]^+^: m/z 262.

#### 4.1.2 General procedure for the synthesis of N'-hydroxybenzamidine derivatives (5a-d)

A solution of hydroxylamonium chloride (2 equiv) and sodium carbonate (1 equiv) dissolved in 15 ml H_2_O was added to a mixture of a nitrile **4 **(1 equiv) dissolved in 15 ml ethanol 96 %, which were then heated under reflux. After 24 h, the mixture was extracted with diethyl ether and concentrated in vacuum. Finally, the light yellow powder was achieved with no purification. 

##### 4.1.2.1 N'-hydroxybenzamidine (5a)

Light yellow powder; yield: 96.5 %; mp: 68-70 °C; IR (KBr, cm^-1^): 1657 (C=N), 3349, 3468 (NH_2_); LC-MS [M + 1]^+^: m/z 136.9.

##### 4.1.2.2 N'-hydroxy-4-methylbenzamidine (5b)

Light yellow powder; yield: 81.9 %; mp: 145.8-148 °C; IR (KBr, cm^-1^): 1661 (C=N), 3365, 3493 (NH_2_); LC-MS [M + 1]^+^: m/z 150.9.

##### 4.1.2.3 4-chloro-N′-hydroxy benzamidine (5c)

Light yellow powder; yield: 87.5 %; mp: 125.8-128 °C; IR (KBr, cm^-1^): 1655 (C=N), 3337, 3462 (NH_2_); LC-MS [M + 1]^+^: m/z 170.8.

##### 4.1.2.4 4-fluoro-N′-hydroxy benzamidine (5d) 

Light yellow powder; yield: 68.2 %; mp: 94.5-99.5 °C; IR (KBr, cm^-1^): 1653 (C=N), 3363, 3457 (NH_2_); LC-MS [M + 1]^+^: m/z 154.9.

#### 4.1.3 General procedure for the synthesis of 1,2,4-oxadiazole derivatives (6a-t)

A suitable *N*-benzylated ester **3a-e **(5 equiv) was dissolved in 15 ml super dry ethanol and stirred under reflux. Subsequently, proper amidoxime **5a-d** (1 equiv) and ethanolic solution of sodium ethoxide 20 % (5 equiv) were added to the obtained mixture. After one day, the mixture was concentrated in vacuum, and the precipitate was washed with *n*-hexane. The *n*-hexane layer was collected and then concentrated under the reduced pressure. Accordingly, the obtained powder was recrystallized from EtOH/H_2_O. The hydrochloride salt of the final product was achieved by the addition of 3 equiv HCl in diethyl ether.

##### 4.1.3.1 5-(1-benzylpiperidin-4-yl)-3-phenyl-1,2,4-oxadiazole (6a)

Light yellow powder; yield: 54.4 %; mp: 70.8-71.2 °C; IR (KBr, cm^-1^): 1587 (C=N), 1142 (C-O); LC-MS [M + 1]^+^: m/z 320; ^1^H NMR (CDCl_3_, 400 MHz) δ: 2.02-2.20 (m, 6H, H-piperidine), 2.94-3.03 (m, 3H, H-piperidine), 3.54 (s, 2H, CH_2_-benzyl), 7.25-7.34 (m, 5H, H_2_, H_3_, H_4, _H_5_, H_6_-benzyl), 7.47-7.48 (m, 3H, H_3_, H_4_, H_5_-phenyl), 8.07-8.08 (m, 2H, H_2_, H_6_-phenyl); ^13^C NMR (CDCl_3_, 100 MHz) δ: 29.56 (2CH_2_), 34.64 (CH), 52.75 (2CH_2_), 63.20 (CH_2_), 126.99 (C), 127.07 (CH), 127.42 (2CH), 128.25 (2CH), 128.80 (2CH), 129.04 (2CH), 131.05 (CH), 138.29 (C), 168.19 (C), 182.07 (C); Anal. calcd for C_20_H_21_N_3_O: C, 75.21; H, 6.63; N, 13.16, found: C, 75.43; H, 6.61; N, 13.13.

##### 4.1.3.2 5-(1-benzylpiperidin-4-yl)-3-(p-tolyl)-1,2,4-oxadiazole (6b)

Light yellow powder; yield: 32.9 %; mp: 88.5-89.8 °C; IR (KBr, cm^-1^): 1582 (C=N), 1145 (C-O), 1346,1440 (CH_3_); LC-MS [M + 1]^+^: m/z 333.9; ^1^H NMR (CDCl_3_, 400 MHz) δ: 2.00-2.18 (m, 6H, H-piperidine), 2.40 (s, 3H, CH_3_), 2.93-3.01 (m, 3H, H-piperidine), 3.53 (s, 2H, CH_2_-benzyl), 7.26-7.28 (m, 2H, H_3_, H_5_-phenyl), 7.32-7.33 (m, 5H, H_2_, H_3_, H_4, _H_5_, H_6_-benzyl), 7.95-7.97 (m, 2H, H_2_, H_6_-phenyl); ^13^C NMR (CDCl_3_, 100 MHz) δ: 21.55 (CH_3_), 29.54 (2CH_2_), 34.62 (CH), 52.74 (2CH_2_), 63.18 (CH_2_), 124.13 (C), 127.04 (2CH), 127.31 (CH), 128.23 (2CH), 129.02 (2CH), 129.49 (2CH), 138.28 (C), 141.31 (C), 168.15 (C), 181.86 (C); Anal. calcd for C_21_H_23_N_3_O: C, 75.65; H, 6.95; N, 12.60, found: C, 75.87; H, 6.92; N, 12.56.

##### 4.1.3.3 5-(1-benzylpiperidin-4-yl)-3-(4-chlorophenyl)-1,2,4-oxadiazole (6c)

Light yellow powder; yield: 32.1 %; mp: 106-108 °C; IR (KBr, cm^-1^): 1592 (C=N), 1139 (C-O); LC-MS [M + 1]^+^: m/z 354; ^1^H NMR (CDCl_3_, 400 MHz) δ: 2.00-2.19 (m, 6H, H-piperidine), 2.94-3.02 (m, 3H, H-piperidine), 3.54 (s, 2H, CH_2_-benzyl), 7.26-7.34 (m, 5H, H_2, _H_3_, H_4_, H_5_, H_6_-benzyl), 7.44-7.46 (m, 2H, H_3_, H_5_-phenyl), 8.00-8.02 (m, 2H, H_2_, H_6_-phenyl); ^13^C NMR (CDCl_3_, 100 MHz) δ: 29.53 (2CH_2_), 34.61 (CH), 52.71 (2CH_2_), 63.18 (CH_2_), 125.47 (C), 127.08 (2CH), 128.75 (CH), 128.72 (2CH), 129.04 (2CH), 129.12 (2CH), 137.17 (C), 138.23 (C), 167.39 (C), 182.30 (C); Anal. calcd for C_20_H_20_ClN_3_O: C, 67.89; H, 5.70; N, 11.88, found: C, 68.12; H, 5.69; N, 11.82. 

##### 4.1.3.4 5-(1-benzylpiperidin-4-yl)-3-(4-fluorophenyl)-1,2,4-oxadiazole (6d)

Light yellow powder; yield: 40.7 %; mp: 92.7-93.6 °C; IR (KBr, cm^-1^): 1571 (C=N), 1130 (C-O); LC-MS [M + 1]^+^: m/z 337.9; ^1^H NMR (CDCl_3_, 400 MHz) δ: 1.93-2.12 (m, 6H, H-piperidine), 2.87-2.93 (m, 3H, H-piperidine), 3.47 (s, 2H, CH_2_-benzyl), 7.06-7.10 (m, 2H, H_3_, H_5_-phenyl), 7.18-7.26 (m, 5H, H_2, _H_3_, H_4, _H_5,_ H_6_-benzyl), 7.98-8.01 (m, 2H, H_2_, H_6_-phenyl); ^13^C NMR (CDCl_3_, 100 MHz) δ: 29.55 (2CH_2_), 34.61 (CH), 52.73 (2CH_2_), 63.20 (CH_2_), 115.90 (C), 123.23 (2CH), 127.12 (CH), 128.28 (2CH), 129.08 (2CH), 129.54 (2CH), 129.62 (C), 138.24 (C), 165.67 (C), 182.21 (C); Anal. calcd for C_20_H_20_FN_3_O: C, 71.20; H, 5.97; N, 12.45, found: C, 71.41; H, 5.99; N, 12.40.

##### 4.1.3.5 5-(1-(4-fluorobenzyl)piperidin-4-yl)-3-phenyl-1,2,4-oxadiazole (6e)

Light yellow powder; yield: 20.9 %; mp: 93.4-94.5 °C; IR (KBr, cm^-1^): 1596 (C=N), 1214 (C-O); LC-MS [M + 1]^+^: m/z 338; ^1^H NMR (CDCl_3_, 400 MHz) δ: 1.97-2.07 (m, 4H, H-piperidine), 2.11-2.19 (m, 4H, H-piperidine), 2.92-3.05 (m, 1H, H-piperidine), 3.50 (s, 2H, CH_2_-benzyl), 7.01 (t, 2H, *J* = 8 Hz, H_3_, H_5_-benzyl), 7.26-7.32 (m, 2H, H_2_, H_6_-benzyl), 7.45-7.50 (m, 3H, H_3_, H_4_, H_5_-phenyl), 8.08 (d, 2H, *J* = 8 Hz, H_2_, H_6_-phenyl); ^13^C NMR (CDCl_3_, 100 MHz) δ: 29.53 (2CH_2_), 34.60 (CH), 52.65 (2CH_2_), 62.35 (CH_2_), 115.15 (2CH), 126.95 (C), 127.42 (2CH), 128.81 (2CH), 130.40 (2CH), 131.08 (CH), 133.98 (C), 160.79 (C), 168.20 (C), 182.00 (C); Anal. calcd for C_20_H_20_FN_3_O: C, 71.20; H, 5.97; N, 12.45, found: C, 71.40; H, 5.94; N, 12.48.

##### 4.1.3.6 5-(1-(4-fluorobenzyl)piperidin-4-yl)-3-(p-tolyl)-1,2,4-oxadiazole (6f)

White powder; yield: 56.3 %; mp: 90-90.8 °C; IR (KBr, cm^-1^): 1582 (C=N), 1223 (C-O), 1344,1440 (CH_3_); LC-MS [M + 1]^+^: m/z 351.9; ^1^H NMR (CDCl_3_, 400 MHz) δ: 2.02-2.17 (m, 6H, H-piperidine), 2.40 (s, 3H, CH_3_), 2.90-3.01 (m, 3H, H-piperidine), 3.49 (s, 2H, CH_2_-benzyl), 6.98-7.03 (m, 2H, H_3_, H_5_-benzyl), 7.26-7.31 (m, 4H, H_2_, H_6_-benzyl, H_3_, H_5_-phenyl), 7.95 (d, 2H, *J* = 8 Hz, H_2_, H_6_-phenyl); ^13^C NMR (CDCl_3_, 100 MHz) δ: 21.55 (CH_3_), 29.53 (2CH_2_), 34.59 (CH), 52.65 (2CH_2_), 62.34 (CH_2_), 115.13 (2CH), 124.13 (C), 127.33 (2CH), 129.51 (2CH), 130.46 (2CH), 134.04 (CH), 141.35 (C), 160.77 (C), 168.18 (C), 181.81 (C); Anal. calcd for C_21_H_22_FN_3_O: C, 71.77; H, 6.31; N, 11.96, found: C, 72.01; H, 6.32; N, 11.91.

##### 4.1.3.7 3-(4-chlorophenyl)-5-(1-(4-fluorobenzyl)piperidin-4-yl)-1,2,4-oxadiazole (6g)

Light yellow powder; yield: 36.0 %; mp: 106.7-107.7 °C; IR (KBr, cm^-1^): 1596 (C=N), 1153 (C-O); LC-MS [M + 1]^+^: m/z 372; ^1^H NMR (CDCl_3_, 400 MHz) δ: 1.96-2.06 (m, 4H, H-piperidine), 2.10-2.19 (m, 4H, H-piperidine), 2.91-3.02 (m, 1H, H-piperidine), 3.50 (s, 2H, CH_2_-benzyl), 7.01 (t, 2H, *J* = 8 Hz, H_3_, H_5_-benzyl), 7.26-7.31 (m, 2H, H_2_, H_6_-benzyl), 7.44 (d, 2H, *J* = 8 Hz, H_3_, H_5_-phenyl), 8.02 (d, 2H, *J* = 8 Hz, H_2_, H_6_-phenyl); ^13^C NMR (CDCl_3_, 100 MHz) δ: 29.52 (2CH_2_), 34.59 (CH), 52.62 (2CH_2_), 62.34 (CH_2_), 115.16 (2CH), 125.48 (C), 128.73 (2CH), 130.39 (2CH), 133.96 (2CH), 137.21 (C), 160.80 (C), 163.23 (C), 167.42 (C), 182.24 (C); Anal. calcd for C_20_H_19_ClFN_3_O: C, 64.60; H, 5.15; N, 11.30, found: C, 64.84; H, 5.14; N, 11.23.

##### 4.1.3.8 5-(1-(4-fluorobenzyl)piperidin-4-yl)-3-(4-fluorophenyl)-1,2,4-oxadiazole (6h)

Light yellow powder; yield: 31.6 %; mp: 115.7-117.7 °C; IR (KBr, cm^-1^): 1609 (C=N), 1230 (C-O); LC-MS [M + 1]^+^: m/z 355.8; ^1^H NMR (CDCl_3_, 400 MHz) δ: 1.90-2.11 (m, 6H, H-piperidine), 2.84-2.95 (m, 3H, H-piperidine), 3.43 (s, 2H, CH_2_-benzyl), 6.91-6.96 (m, 2H, H_3_, H_5_-benzyl), 7.08 (t, 2H, *J* = 8 Hz, H_3_, H_5_-phenyl), 7.19-7.24 (m, 2H, H_2_, H_6_-benzyl), 7.98-8.02 (m, 2H, H_2_, H_6_-phenyl); ^13^C NMR (CDCl_3_, 100 MHz) δ: 29.52 (2CH_2_), 34.57 (CH), 52.63 (2CH_2_), 62.35 (CH_2_), 115.90 (2CH), 123.20 (2CH), 129.53 (C), 130.43 (2CH), 133.97 (2CH), 160.81 (C), 163.27 (C), 165.77 (C), 167.41 (C), 182.13 (C); Anal. calcd for C_20_H_19_F_2_N_3_O: C, 67.59; H, 5.39; N, 11.82, found: C, 67.82; H, 5.36; N, 11.76.

##### 4.1.3.9 5-(1-(4-chlorobenzyl)piperidin-4-yl)-3-phenyl-1,2,4-oxadiazole (6i)

Light yellow powder; yield: 39.4 %; mp: 95.6-96.7 °C; IR (KBr, cm^-1^): 1586 (C=N), 1110 (C-O); LC-MS [M + 1]^+^: m/z 353.8; ^1^H NMR (CDCl_3_, 400 MHz) δ: 1.96-2.18 (m, 6H, H-piperidine), 2.89-3.02 (m, 3H, H-piperidine), 3.48 (s, 2H, CH_2_-benzyl), 7.25-7.30 (m, 4H, H_2_, H_3_, H_5_, H_6_-benzyl), 7.46-7.48 (m, 3H, H_3_, H_4_, H_5_-phenyl), 8.06-8.08 (m, 2H, H_2_, H_6_-phenyl); ^13^C NMR (CDCl_3_, 100 MHz) δ: 29.50 (2CH_2_), 34.51 (CH), 52.65 (2CH_2_), 62.33 (CH_2_), 126.93 (C), 127.33 (2CH), 127.42 (2CH), 128.37 (2CH), 130.22 (2CH), 131.05 (CH), 132.70 (C), 136.89 (C), 168.16 (C), 181.94 (C); Anal. calcd for C_20_H_20_ClN_3_O: C, 67.89; H, 5.70; N, 11.88, found: C, 68.12; H, 5.68; N, 11.93.

##### 4.1.3.10 5-(1-(4-chlorobenzyl)piperidin-4-yl)-3-(p-tolyl)-1,2,4-oxadiazole (6j)

Light yellow powder; yield: 40.3 %; mp: 103.5-104.7 °C; IR (KBr, cm^-1^): 1582 (C=N), 1213 (C-O), 1358,1486 (CH_3_); LC-MS [M + 1]^+^: m/z 367.8; ^1^H NMR (CDCl_3_, 400 MHz) δ: 1.89-2.11 (m, 6H, H-piperidine), 2.33 (s, 3H, CH_3_), 2.82-2.94 (m, 3H, H-piperidine), 3.42 (s, 2H, CH_2_-benzyl), 7.18-7.23 (m, 6H, H_2_, H_3_, H_5_, H_6_-benzyl, H_3_, H_5_-phenyl), 7.87 (d, 2H, *J* = 8 Hz, H_2_, H_6_-phenyl); ^13^C NMR (CDCl_3_, 100 MHz) δ: 21.59 (CH_3_), 29.52 (2CH_2_), 34.55 (CH), 52.70 (2CH_2_), 62.37 (CH_2_), 124.13 (C), 127.35 (2CH), 128.43 (2CH), 129.54 (2CH), 130.28 (2CH), 132.78 (C), 136.89 (C), 141.39 (C), 168.22 (C), 181.78 (C); Anal. calcd for C_21_H_22_ClN_3_O: C, 68.56; H, 6.03; N, 11.42, found: C, 68.79; H, 6.01; N, 11.37. 

##### 4.1.3.11 5-(1-(4-chlorobenzyl)piperidin-4-yl)-3-(4-chlorophenyl)-1,2,4-oxadiazole (6k)

Light yellow powder; yield: 51.7 %; mp: 110.7-113.7 °C; IR (KBr, cm^-1^): 1582 (C=N), 1213 (C-O); LC-MS [M + 1]^+^: m/z 387.8; ^1^H NMR (CDCl_3_, 400 MHz) δ: 1.88-2.11 (m, 6H, H-piperidine), 2.82-2.95 (m, 3H, H-piperidine), 3.41 (s, 2H, CH_2_-benzyl), 7.18-7.22 (m, 4H, H_2_, H_3_, H_5_, H_6_-benzyl), 7.35 (d, 2H, *J* = 8 Hz, H_3_, H_5_-phenyl), 7.92 (d, 2H, *J* = 8 Hz, H_2_, H_6_-phenyl); ^13^C NMR (CDCl_3_, 100 MHz) δ: 29.47 (2CH_2_), 34.50 (CH), 52.63 (2CH_2_), 62.36 (CH_2_), 125.45 (C), 128.75 (2CH), 129.16 (2CH), 129.46 (2CH), 130.32 (2CH), 132.82 (C), 136.73 (C), 137.23 (C), 167.41 (C), 182.19 (C); Anal. calcd for C_20_H_19_Cl_2_N_3_O: C, 61.86; H, 4.93; N, 10.82, found: C, 62.09; H, 4.91; N, 10.78.

##### 4.1.3.12 5-(1-(4-chlorobenzyl)piperidin-4-yl)-3-(4-fluorophenyl)-1,2,4-oxadiazole (6l)

Light yellow powder; yield: 40.7 %; mp: 89.9-90.4 °C; IR (KBr, cm^-1^): 1600 (C=N), 1221 (C-O); LC-MS [M + 1]^+^: m/z 371.8; ^1^H NMR (CDCl_3_, 400 MHz) δ: 1.99-2.19 (m, 6H, H-piperidine), 2.90-3.02 (m, 3H, H-piperidine), 3.49 (s, 2H, CH_2_-benzyl), 7.13-7.18 (m, 2H, H_3_, H_5_-phenyl), 7.26-7.29 (m, 4H, H_2_, H_3_, H_5_, H_6_-benzyl), 8.05-8.09 (m, 2H, H_2_, H_6_-phenyl); ^13^C NMR (CDCl_3_, 100 MHz) δ: 29.51 (2CH_2_), 34.51 (CH), 52.66 (2CH_2_), 62.35 (CH_2_), 115.88 (2CH), 123.16 (C), 129.51 (2CH), 130.23 (2CH), 132.74 (2CH), 136.86 (C), 163.24 (C), 165.73 (C), 167.37 (C), 182.09 (C); Anal. calcd for C_20_H_19_ClFN_3_O: C, 64.60; H, 5.15; N, 11.30, found: C, 64.81; H, 5.12; N, 11.37. 

##### 4.1.3.13 5-(1-(2-chlorobenzyl)piperidin-4-yl)-3-phenyl-1,2,4-oxadiazole (6m)

Light yellow powder; yield: 28.6 %; mp: 61.5-62.2 °C; IR (KBr, cm^-1^): 1583 (C=N), 1134 (C-O); LC-MS [M + 1]^+^: m/z 354; ^1^H NMR (CDCl_3_, 400 MHz) δ: 2.01-2.18 (m, 6H, H-piperidine), 2.93-3.02 (m, 3H, H-piperidine), 3.50 (s, 2H, CH_2_-benzyl), 7.13-7.15 (m, 3H, H_4_, H_5_, H_6_-benzyl), 7.21-7.25 (m, 3H, H_3_, H_4_, H_5_-phenyl), 7.44-7.49 (m, 1H, H_3_-benzyl), 8.06 (d, 2H, *J* = 8 Hz, H_2_, H_6_-phenyl); ^13^C NMR (CDCl_3_, 100 MHz) δ: 29.56 (2CH_2_), 34.67 (CH), 52.69 (2CH_2_), 62.92 (CH_2_), 126.89 (C), 127.00 (CH), 127.42 (2CH), 128.79 (2CH), 128.93 (2CH), 129.04 (CH), 131.04 (CH), 135.14 (C), 136.66 (C), 168.18 (C), 182.09 (C); Anal. calcd for C_20_H_20_ClN_3_O: C, 67.89; H, 5.70; N, 11.88, found: C, 68.13; H, 5.67; N, 11.80.

##### 4.1.3.14 5-(1-(2-chlorobenzyl)piperidin-4-yl)-3-(p-tolyl)-1,2,4-oxadiazole (6n)

White powder; yield: 33.8 %; mp: 121.8-124.7 °C; IR (KBr, cm^-1^): 1582 (C=N), 1146 (C-O), 1442,1350 (CH_3_); LC-MS [M + 1]^+^: m/z 367.9; ^1^H NMR (CDCl_3_, 400 MHz) δ: 2.09-2.14 (m, 4H, H-piperidine), 2.24-2.31 (m, 2H, H-piperidine), 2.40 (s, 3H, CH_3_), 2.97-3.04 (m, 3H, H-piperidine), 3.65 (s, 2H, CH_2_-benzyl), 7.17-7.28 (m, 3H, H_4_, H_5_, H_6_-benzyl), 7.34 (d, 2H, *J* = 8 Hz, H_3_, H_5_-phenyl), 7.50-7.51 (m, 1H, H_3_-benzyl), 7.95 (d, 2H, *J* = 8 Hz, H_2_, H_6_-phenyl); ^13^C NMR (CDCl_3_, 100 MHz) δ: 21.56 (CH_3_), 29.62 (2CH_2_), 34.56 (CH), 52.85 (2CH_2_), 59.34 (CH_2_), 124.14 (C), 126.62 (2CH), 127.33 (CH), 128.07 (2CH), 129.51 (2CH), 130.48 (CH), 134.19 (C), 136.05 (C), 141.33 (C), 168.18 (C), 181.85 (C); Anal. calcd for C_21_H_22_ClN_3_O: C, 68.56; H, 6.03; N, 11.42, found: C, 68.75; H, 5.99; N, 11.38.

##### 4.1.3.15 5-(1-(2-chlorobenzyl)piperidin-4-yl)-3-(4-chlorophenyl)-1,2,4-oxadiazole (6o)

White powder; yield: 33.8 %; mp: 85.5-87 °C; IR (KBr, cm^-1^): 1597 (C=N), 1141 (C-O); LC-MS [M + 1]^+^: m/z 387.8; ^1^H NMR (CDCl_3_, 400 MHz) δ: 1.95-2.07 (m, 4H, H-piperidine), 2.19-2.24 (m, 2H, H-piperidine), 2.90-2.97 (m, 3H, H-piperidine), 3.58 (s, 2H, CH_2_-benzyl), 7.14-7.19 (m, 2H, H_4_, H_5_-benzyl), 7.27 (d, 1H, *J* = 8 Hz ,H_3_-benzyl), 7.36 (d, 2H, *J* = 8 Hz, H_3_, H_5_-phenyl), 7.42 (d, 1H, *J* = 8 Hz ,H_6_-benzyl), 7.93 (d, 2H, *J* = 8 Hz, H_2_, H_6_-phenyl); ^13^C NMR (CDCl_3_, 100 MHz) δ: 29.60 (2CH_2_), 34.55 (CH), 52.81 (2CH_2_), 59.35 (CH_2_), 125.50 (C), 126.67 (CH), 128.16 (2CH), 129.16 (2CH), 129.45 (2CH), 130.54 (CH), 134.25 (C), 135.99 (C), 137.21 (C), 167.43 (C), 182.28 (C); Anal. calcd for C_20_H_19_Cl_2_N_3_O: C, 61.86; H, 4.93; N, 10.82, found: C, 62.09; H, 4.92; N, 10.75. 

##### 4.1.3.16 5-(1-(2-chlorobenzyl)piperidin-4-yl)-3-(4-fluorophenyl)-1,2,4-oxadiazole (6p)

Light yellow powder; yield: 30.5 %; mp: 69.5-70 °C; IR (KBr, cm^-1^): 1600 (C=N), 1212 (C-O); LC-MS [M + 1]^+^: m/z 371.8; ^1^H NMR (CDCl_3_, 400 MHz) δ: 2.05-2.17 (m, 4H, H-piperidine), 2.29-2.34 (m, 2H, H-piperidine), 3.00-3.03 (m, 3H, H-piperidine), 3.68 (s, 2H, CH_2_-benzyl), 7.20-7.29 (m, 3H, H_4_, H_5_, H_6_-benzyl), 7.37 (d, 2H, *J* = 8 Hz, H_3_, H_5_-phenyl), 7.53-7.54 (m, 1H, H_3_-benzyl), 8.08-8.12 (m, 2H, H_2_, H_6_-phenyl); ^13^C NMR (CDCl_3_, 100 MHz) δ: 29.61 (2CH_2_), 34.54 (CH), 52.82 (2CH_2_), 59.35 (CH_2_), 115.90 (C), 123.22 (CH), 126.66 (2CH), 128.15 (2CH), 129.45 (2CH), 130.54 (CH), 134.24 (C), 136.00 (C), 163.27 (C), 167.41 (C), 182.18 (C); Anal. calcd for C_20_H_19_ClFN_3_O: C, 64.60; H, 5.15; N, 11.30, found: C, 64.79; H, 5.13; N, 11.23. 

##### 4.1.3.17 5-(1-(4-methylbenzyl)piperidin-4-yl)-3-phenyl-1,2,4-oxadiazole (6q)

Light yellow powder; yield: 32.8 %; mp: 90.3-91.4 °C; IR (KBr, cm^-1^): 1589 (C=N), 1145 (C-O), 1363,1440 (CH_3_); LC-MS [M + 1]^+^: m/z 334; ^1^H NMR (CDCl_3_, 400 MHz) δ: 2.09-2.14 (m, 6H, H-piperidine), 2.26-2.31 (m, 3H, CH_3_), 2.98-3.05 (m, 3H, H-piperidine), 3.65 (s, 2H, CH_2_-benzyl), 7.17-7.27 (m, 2H, H_3_, H_5_-benzyl), 7.34 (d, 2H, *J* = 8 Hz, H_2_, H_6_-benzyl),7.47-7.52 (m, 3H, H_3_, H_4_, H_5_-phenyl), 8.07 (d, 2H, *J* = 8 Hz, H_2_, H_6_-phenyl); ^13^C NMR (CDCl_3_, 100 MHz) δ: 21.55 (CH_3_), 29.62 (2CH_2_), 34.56 (CH), 52.84 (2CH_2_), 59.35 (CH_2_), 126.63 (C), 127.42 (2CH), 128.80 (2CH), 129.41 (2CH), 130.50 (2CH), 131.06 (CH), 134.21 (C), 136.04 (C), 168.20 (C), 182.04 (C); Anal. calcd for C_21_H_23_N_3_O: C, 75.65; H, 6.95; N, 12.60, found: C, 75.86; H, 6.94; N, 12.55.

##### 4.1.3.18 5-(1-(4-methylbenzyl)piperidin-4-yl)-3-(p-tolyl)-1,2,4-oxadiazole (6r)

Light yellow powder; yield: 47.2 %; mp: 84.5-85.3 °C; IR (KBr, cm^-1^): 1579 (C=N), 1117 (C-O), 1358,1410 (CH_3_); LC-MS [M + 1]^+^: m/z 347.9; ^1^H NMR (CDCl_3_, 400 MHz) δ: 2.02-2.15 (m, 6H, H-piperidine), 2.33 (s , 3H , CH_3_-benzyl), 2.39 (s , 3H , CH_3_-phenyl), 2.92-2.96 (m, 3H, H-piperidine), 3.49 (s, 2H, CH_2_-benzyl), 7.12 (d, 2H, *J* = 8 Hz, H_3_, H_5_-benzyl), 7.20 (d, 2H, *J* = 8 Hz, H_2_, H_6_-benzyl), 7.25 (d, 2H, *J* = 8 Hz, H_3_, H_5_-phenyl), 7.95 (d, 2H, *J* = 8 Hz, H_2_, H_6_-phenyl); ^13^C NMR (CDCl_3_, 100 MHz) δ: 21.10 (CH_3_), 21.54 (CH_3_), 29.54 (2CH_2_), 34.64 (CH), 52.68 (2CH_2_), 62.90 (CH_2_), 124.15 (C), 127.31 (2CH), 128.91 (2CH), 129.00 (2CH), 129.48 (2CH), 135.15 (C), 136.60 (C), 141.28 (C), 168.14 (C), 181.88 (C); Anal. calcd for C_22_H_25_N_3_O: C, 76.05; H, 7.25; N, 12.09, found: C, 76.28; H, 7.23; N, 12.02.

##### 4.1.3.19 3-(4-chlorophenyl)-5-(1-(4-methylbenzyl)piperidin-4-yl)-1,2,4-oxadiazole (6s)

Light yellow powder; yield: 47.3 %; mp: 111.4-112.5 °C; IR (KBr, cm^-1^): 1585 (C=N), 1128 (C-O), 1361,1446 (CH_3_); LC-MS [M + 1]^+^: m/z 367.8; ^1^H NMR (CDCl_3_, 400 MHz) δ: 1.95-2.10 (m, 6H, H-piperidine), 2.26 (s, 3H, CH_3_), 2.86-2.91 (m, 3H, H-piperidine), 3.43 (s, 2H, CH_2_-benzyl), 7.05 (d, 2H, *J* = 8 Hz, H_3_, H_5_-benzyl), 7.15 (d, 2H, *J* = 8 Hz, H_2_, H_6_-benzyl), 7.35 (d, 2H, *J* = 8 Hz, H_3_, H_5_-phenyl), 7.92 (d, 2H, *J* = 8 Hz, H_2_, H_6_-phenyl); ^13^C NMR (CDCl_3_, 100 MHz) δ: 21.15 (CH_3_), 29.55 (2CH_2_), 34.66 (CH), 52.68 (2CH_2_), 62.93 (CH_2_), 125.53 (C), 128.76 (2CH), 128.97 (2CH), 129.07 (2CH), 129.15 (2CH), 135.09 (C), 136.72 (C), 137.19 (C), 167.42 (C), 182.35 (C); Anal. calcd for C_21_H_22_ClN_3_O: C, 68.56; H, 6.03; N, 11.42, found: C, 68.79; H, 6.01; N, 11.38.

##### 4.1.3.20 3-(4-fluorophenyl)-5-(1-(4-methylbenzyl)piperidin-4-yl)-1,2,4-oxadiazole (6t) 

Light yellow powder; yield: 48.2 %; mp: 96.5-97.2 °C; IR (KBr, cm^-1^): 1600 (C=N), 1223 (C-O), 1352,1444 (CH_3_); LC-MS [M + 1]^+^: m/z 351.7; ^1^H NMR (CDCl_3_, 400 MHz) δ: 1.99-2.17 (m, 6H, H-piperidine), 2.34 (s, 3H, CH_3_), 2.94-2.98 (m, 3H, H-piperidine), 3.50 (s, 2H, CH_2_-benzyl), 7.13-7.17 (m, 4H, H_2, _H_3_, H_5_, H_6_-benzyl), 7.23 (t, 2H, *J* = 8 Hz, H_3_, H_5_-phenyl), 8.05-8.09 (m, 2H, H_2_, H_6_-phenyl); ^13^C NMR (CDCl_3_, 100 MHz) δ: 21.11 (CH_3_), 29.54 (2CH_2_), 34.64 (CH), 52.67 (2CH_2_), 62.92 (CH_2_), 115.86 (2CH), 123.20 (C), 128.93 (2CH), 129.51 (2CH), 135.09 (2CH), 136.68 (C), 163.23 (C), 165.72 (C), 167.36 (C), 182.22 (C); Anal. calcd for C_21_H_22_FN_3_O: C, 71.77; H, 6.31; N, 11.96, found: C, 72.02; H, 6.30; N, 11.87. 

#### 4.2 Inhibitory activity against AChE and BuChE enzymes

Acetylcholinesterase inhibitory activity was determined using the 5,5-dithiobis-2-nitrobenzoic acid (DTNB) assay as described by Ellman et al. (1961[12]). AChE (E.C. 3.1.1.7, type V-S, lyophilized powder, from the electric eel, 1000 units) and BuChE (E.C. 3.1.1.8, from equine serum), acetylthiocholine iodide (ATCI), butyrylthiocholine iodide (BTCI), and 5,5-dithiobis-(2-nitrobenzoic acid) (DTNB) were all acquired from Sigma-Aldrich. Potassium dihydrogen phosphate, dipotassium hydrogen phosphate, potassium hydroxide, and sodium hydrogen carbonate were obtained from Fluka. Donepezil was used as a reference compound. Assay solutions were then prepared by the addition of compounds **6a-t **to a mixture containing DMSO (5 mL) and methanol (5mL) diluted in potassium phosphate buffer (0.1 M, pH=8.0). In this regard, each one of the wells included a 50 µL potassium phosphate buffer, 25 µL sample dissolved in 50 % methanol and 50 % DMSO, and 25 µL enzyme (the final concentration 0.22 U/mL in buffer). Thereafter, the wells were pre-incubated for 15 min at room temperature, and 125 µL DTNB (3 mM in buffer) was added to each plate. Followed by the addition of substrate (ATCI 3 mM in water), the absorbance change was measured using a 96-well plate reader (BioTek ELx808) at 405 nm after 15 min. As well, the IC_50_ values were expressed as mean ± SD. The percentage of enzyme's inhibition was calculated by comparing with a blank sample (100 % activity). The described method was also used for the BuChE inhibition assay.

#### 4.3 Docking study 

The AutoDock Tools version 1.5.6rc3 (http://mgltools.scripps.edu/) was applied for docking study of the compound **6n**. In the current study, the X-ray crystallographic structure of BuChE (PDB code 1P0I) was obtained from the Protein Data Bank. Subsequently, all water molecules in the PDB file were removed, hydrogen atoms were added to amino acid residues, and Gasteiger charges were assigned to all atoms of the enzyme. The structure of the compound **6n** was optimized by the MM+ force field using HyperChem8 (http://www.hyper.com) and then converted to pdbqt format file using AutoDock Tools. The grid size was set at 40 × 40 × 40 with a grid spacing of 0.375 Å, and the grid center was determined at dimensions (x, y, and z): 137.44, 114.33, and 39.22, respectively. Each docked system was performed by 100 runs of the AutoDock search using the Lamarckian genetic algorithm (LGA). Finally, the lowest energy conformations were selected for analyzing the interactions between the enzyme and inhibitor. Moreover, graphic manipulations and visualizations were done by Pymol software version 1.5.0.1 (http://pymol.findmysoft.com/). 

#### 4.4 ADME properties

The ADME properties of the synthesized compounds in this study were predicted using the SwissADME online property calculator (http://swissadme.ch/) (Daina et al., 2017[9]). Notably, topological polar surface area (TPSA), number of rotatable bonds (*n*-ROTB), molecular weight (MW), the logarithm of the partition coefficient (miLog *P*), number of hydrogen bond acceptors (*n*-ON), number of hydrogen bond donors (*n*-OHNH), and Lipinski's rule of five criteria were calculated as well (Lipinski et al., 2001[20]). Additionally, the following equation was utilized to calculate the intestinal absorption percent (% ABS): % ABS = 109 − (0.345 × TPSA) (Zhao et al., 2002[34]).

## Notes

Elham Rezaee and Sayyed Abbas Tabatabai (Department of Pharmaceutical Chemistry, School of Pharmacy, Shahid Beheshti University of Medical Sciences, Tehran, Iran, No. 2660, Vali-e-Asr., Tehran 1991953381, Iran; Tel: 00982188200093, Fax: 00982188665341, E-mail: sa_tabatabai@sbmu.ac.ir) contributed equally as corresponding author.

## Acknowledgements

This work was supported by a grant from the Research Council of Shahid Beheshti University of Medical Sciences (Grant No; 7763).

## Supplementary Material

Supplementary information

## Figures and Tables

**Table 1:**
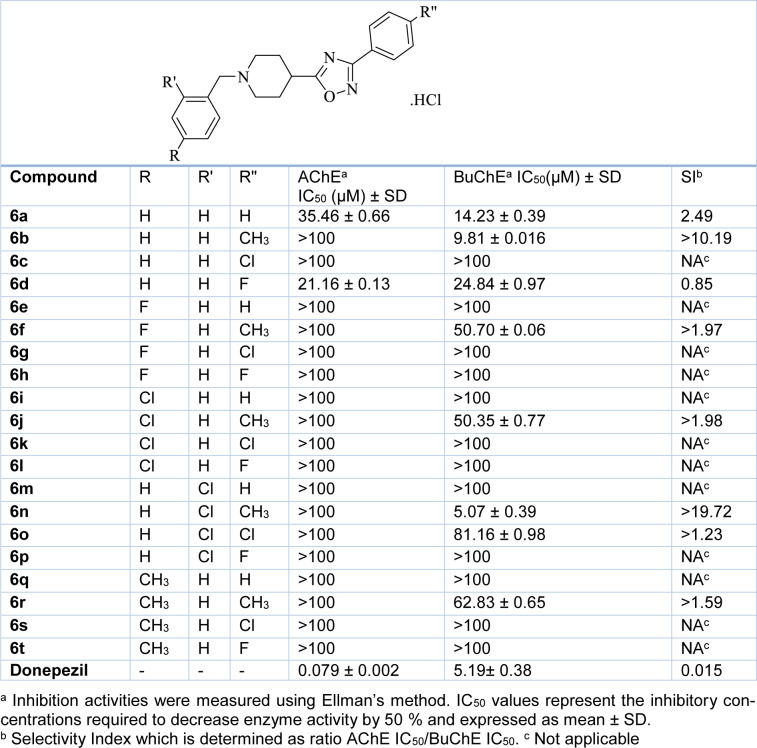
Inhibitory activities of the 1,2,4-oxadiazole derivatives (6a-t)

**Table 2:**
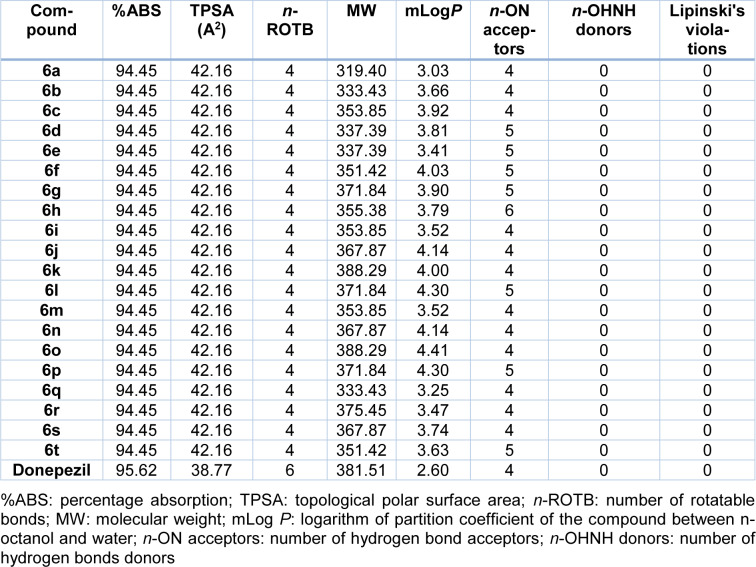
Pharmacokinetic parameters important for oral bioavailability of the synthesized compounds (6a-t)

**Figure 1:**
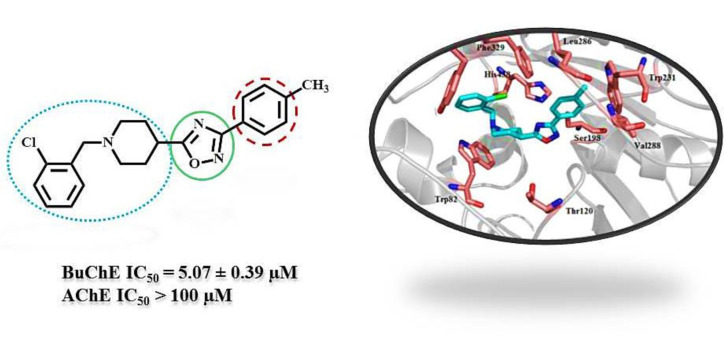
* N*-benzylpiperidine-based derivatives of 1,2,4-oxadiazole as novel selective inhibitors of butyrylcholinesterase enzyme
