# Supplementary material for: Novel 1,2,4-oxadiazole derivatives as selective butyrylcholinesterase inhibitors: Design, synthesis and biological evaluation
Source: EXCLI J. 2021 May 18;20:907–21. doi: 10.17179/excli2021-3569 (PMC8192883; doi:10.17179/excli2021-3569)

EXCLI Journal 2021;20:907-921 – ISSN 1611-2156
Received: February 27, 2021, accepted: May 10, 2021, published: May 18, 2021

Supplementary information to:

Original article:

NOVEL 1,2,4-OXADIAZOLE DERIVATIVES AS SELECTIVE BUTYRYLCHOLINESTERASE INHIBITORS: DESIGN, SYNTHESIS, AND BIOLOGICAL EVALUATION

Maryam Nazari[a], Elham Rezaee[a,*], Roshanak Hariri[b], Tahmineh Akbarzadeh[b], Sayyed Abbas Tabatabai[a,*]

[a] Department of Pharmaceutical Chemistry, School of Pharmacy, Shahid Beheshti University of Medical Sciences, Tehran, Iran
[b] Department of Medicinal Chemistry, Faculty of Pharmacy, Tehran University of Medical Sciences, Tehran, Iran

* **Corresponding authors:** Sayyed Abbas Tabatabai and Elham Rezaee, Department of Pharmaceutical Chemistry, School of Pharmacy, Shahid Beheshti University of Medical Sciences, Tehran, Iran. No. 2660, Vali-e-Asr., Tehran 1991953381, Iran, Tel: 00982188200093; Fax: 00982188665341;
E-mails: sa_tabatabai@sbmu.ac.ir (Sayyed Abbas Tabatabai); e.rezaee63@sbmu.ac.ir (Elham Rezaee)

http://dx.doi.org/10.17179/excli2021-3569

This is an Open Access article distributed under the terms of the Creative Commons Attribution License (http://creativecommons.org/licenses/by/4.0/).

Table of contents

Characterization of compound **3a-3e**
Characterization of compound **5a-5d**
Characterization of compounds **6a-6t**

EXCLI Journal 2021;20:907-921 – ISSN 1611-2156
Received: February 27, 2021, accepted: May 10, 2021, published: May 18, 2021

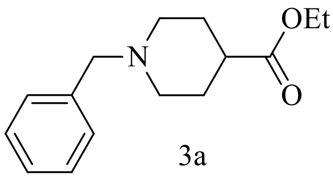

3a

Ethyl 1-benzylpiperidine-4-carboxylate (3a)
Yellow oily liquid (59.7 % yield); IR (KBr, cm^{-1}): 1733 (C=O); LC-MS $[M + 1]^+$: m/z 248.

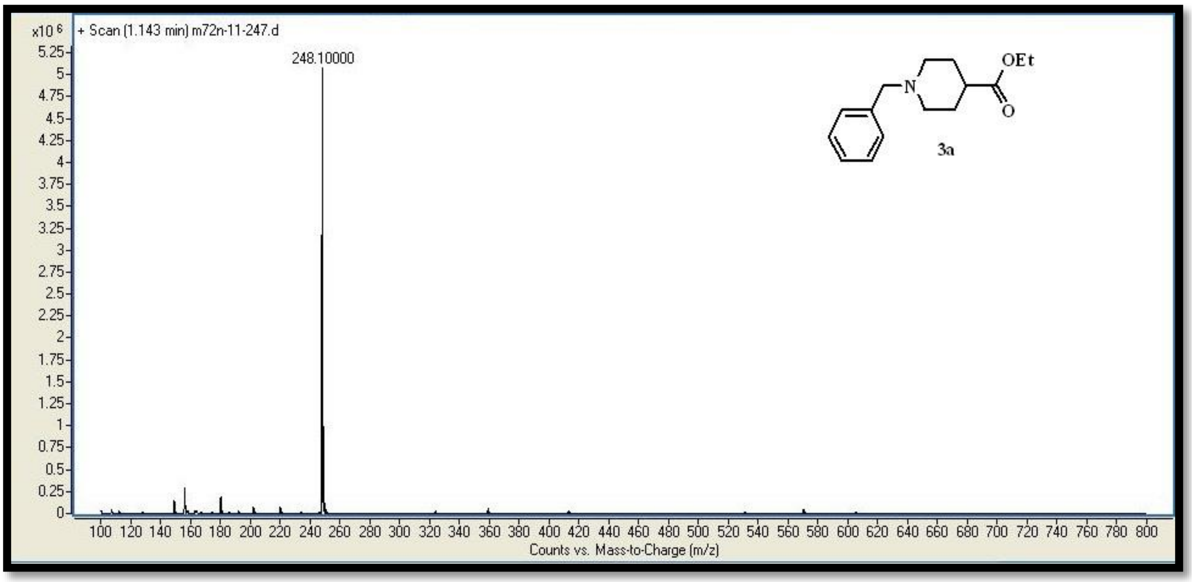

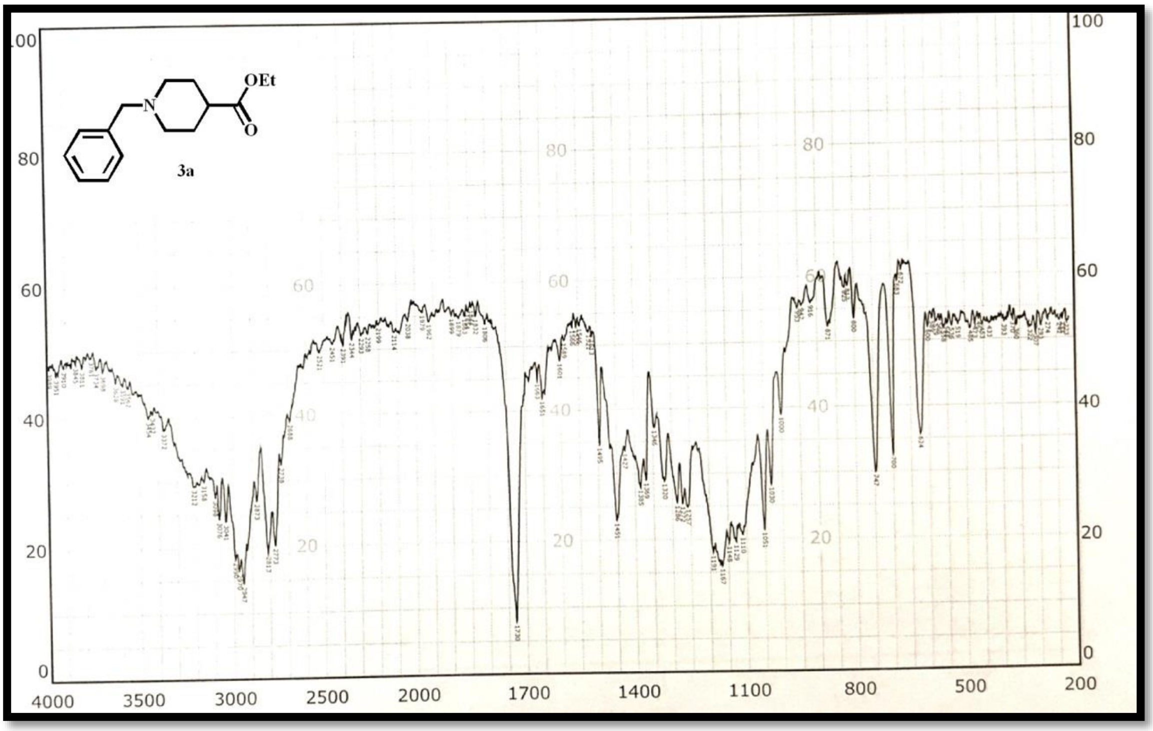

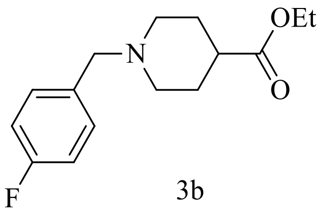

3b

Ethyl 1-(4-fluorobenzyl)piperidine-4-carboxylate (3b)

Yellow oily liquid (73.5 % yield); IR (KBr, cm^{-1}): 1732 (C=O); LC-MS $[M + 1]^+$: m/z 265.9.

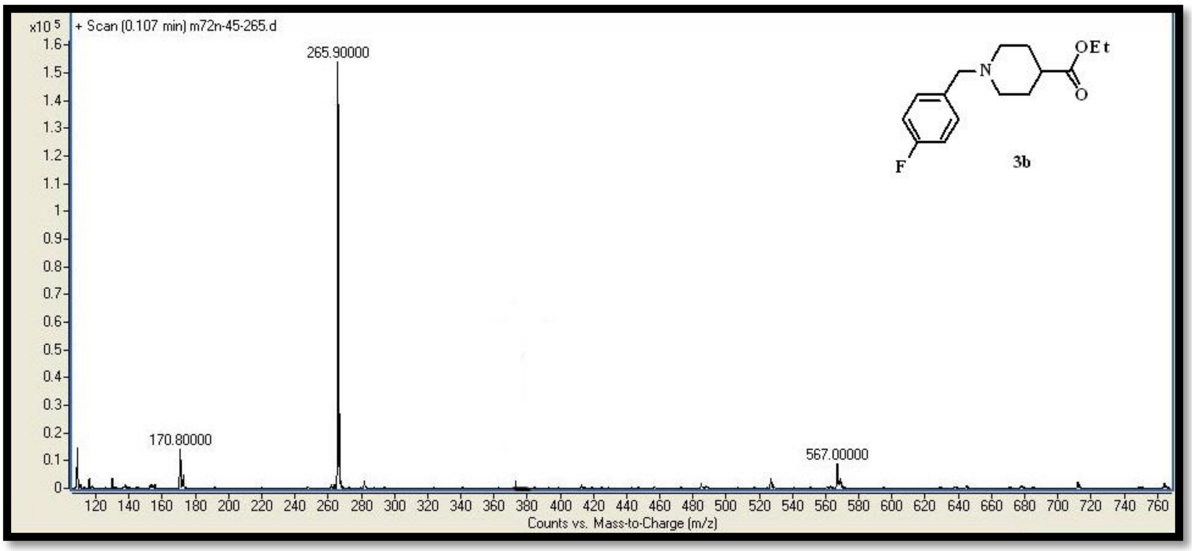

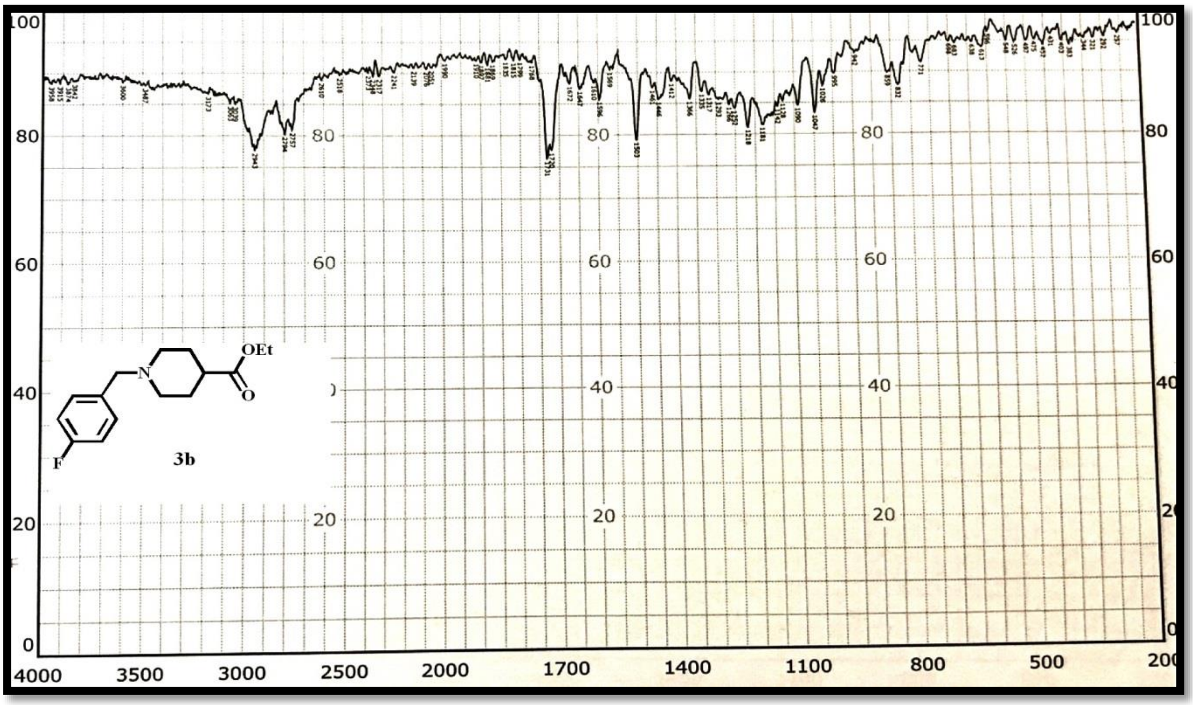

EXCLI Journal 2021;20:907-921 – ISSN 1611-2156
Received: February 27, 2021, accepted: May 10, 2021, published: May 18, 2021

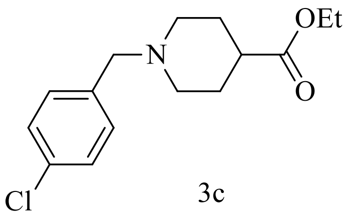

3c

Ethyl 1-(4-chlorobenzyl)piperidine-4-carboxylate (3c)
Yellow oily liquid (68.9 % yield); IR (KBr, cm^{-1}): 1738 (C=O); LC-MS [M + 1]$^{+}$: m/z 281.9.

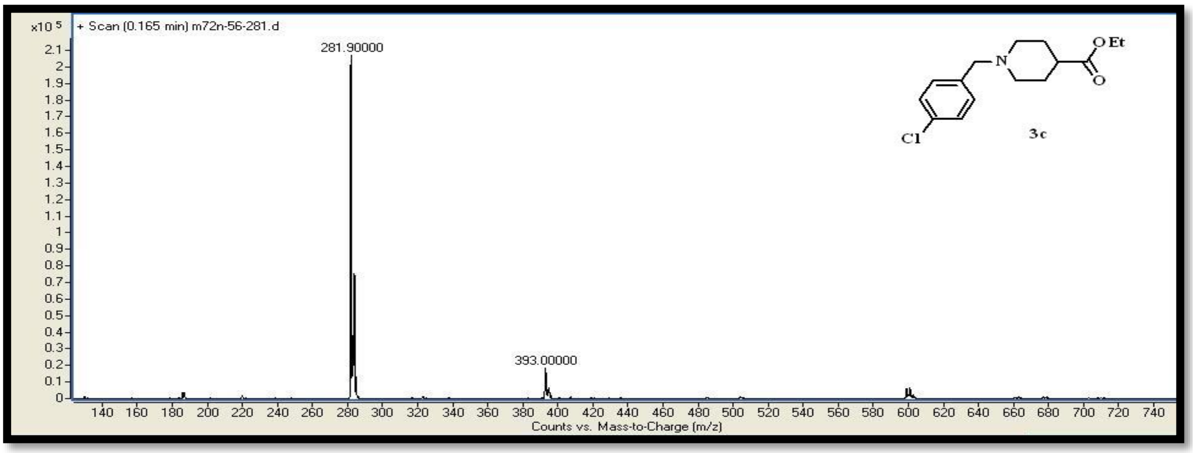

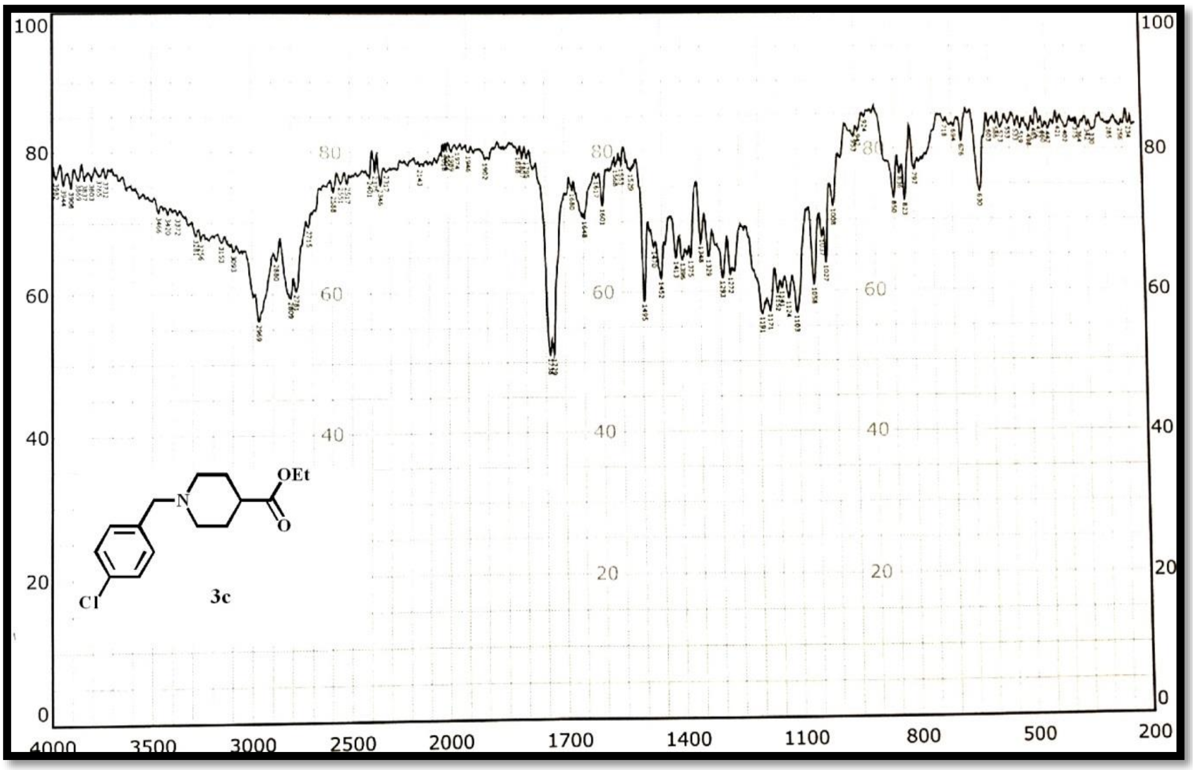

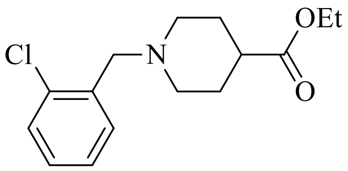

3d

Ethyl 1-(2-chlorobenzyl)piperidine-4-carboxylate (3d)
Yellow oily liquid (60.6 % yield); IR (KBr, cm^{-1}): 1738 (C=O); LC-MS $[M + 1]^+$: m/z 282.

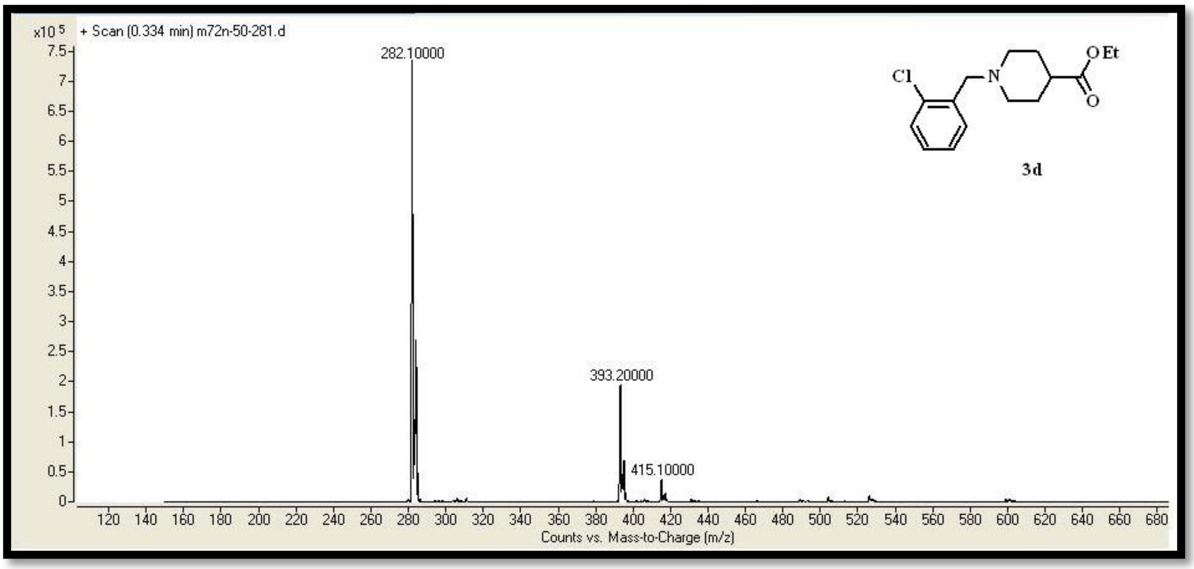

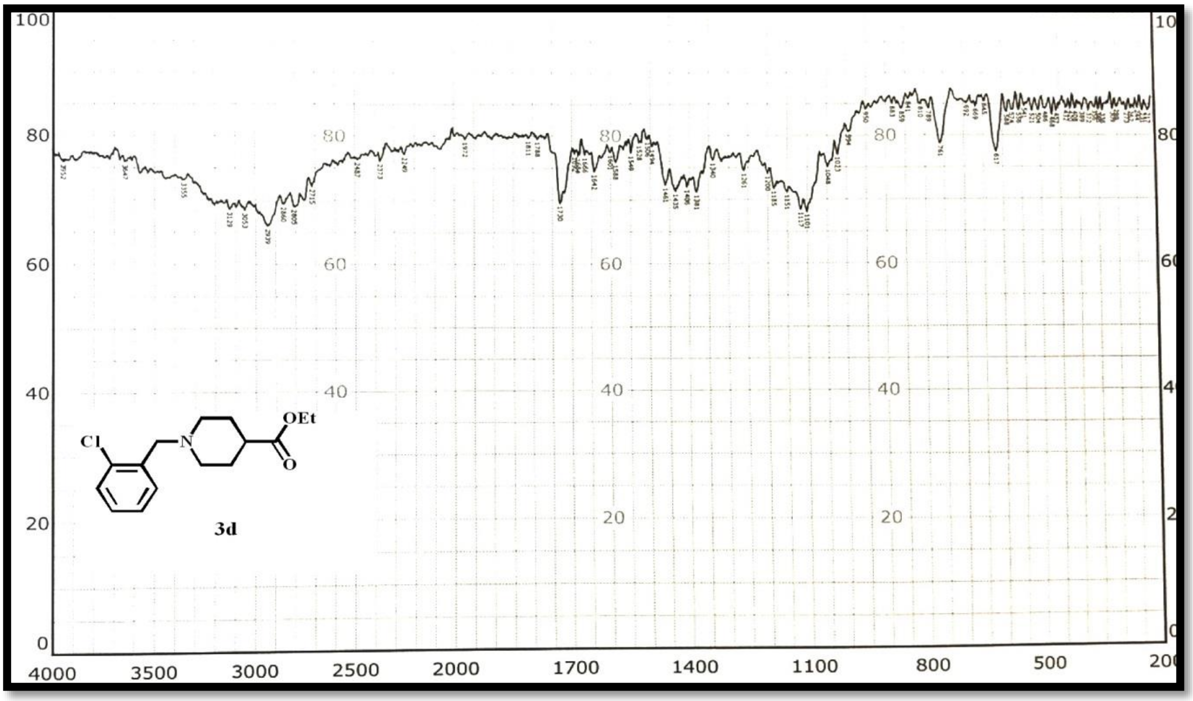

EXCLI Journal 2021;20:907-921 – ISSN 1611-2156
Received: February 27, 2021, accepted: May 10, 2021, published: May 18, 2021

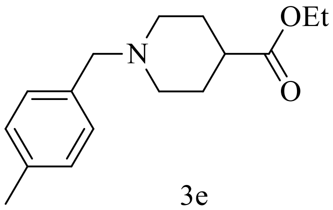

3e

Ethyl 1-(4-methylbenzyl)piperidine-4-carboxylate (3e)
Yellow oily liquid (76.6 % yield); IR (KBr, cm^{-1}): 1733 (C=O); LC-MS $[M + 1]^+$: m/z 262.

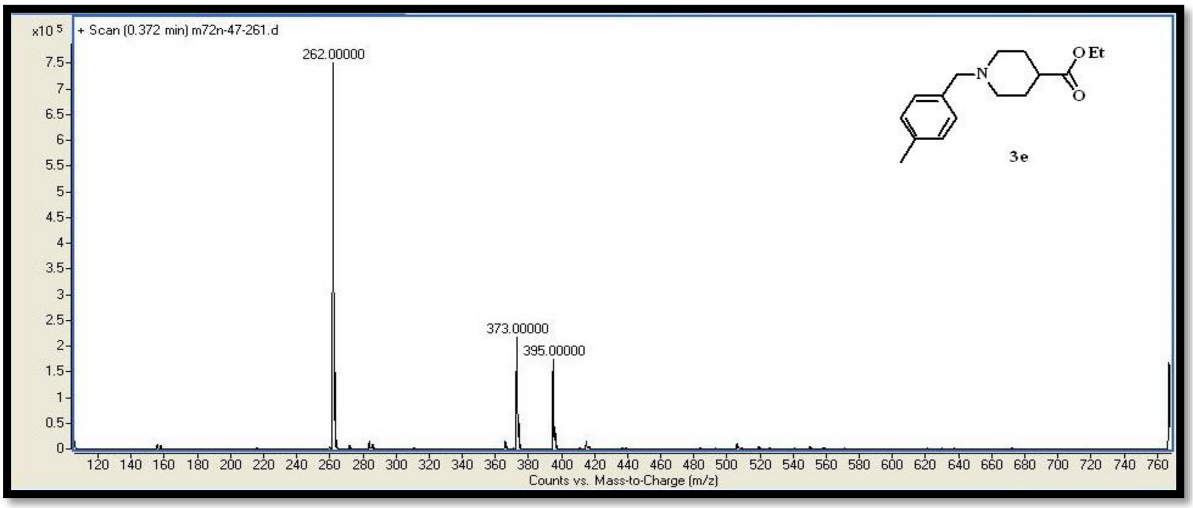

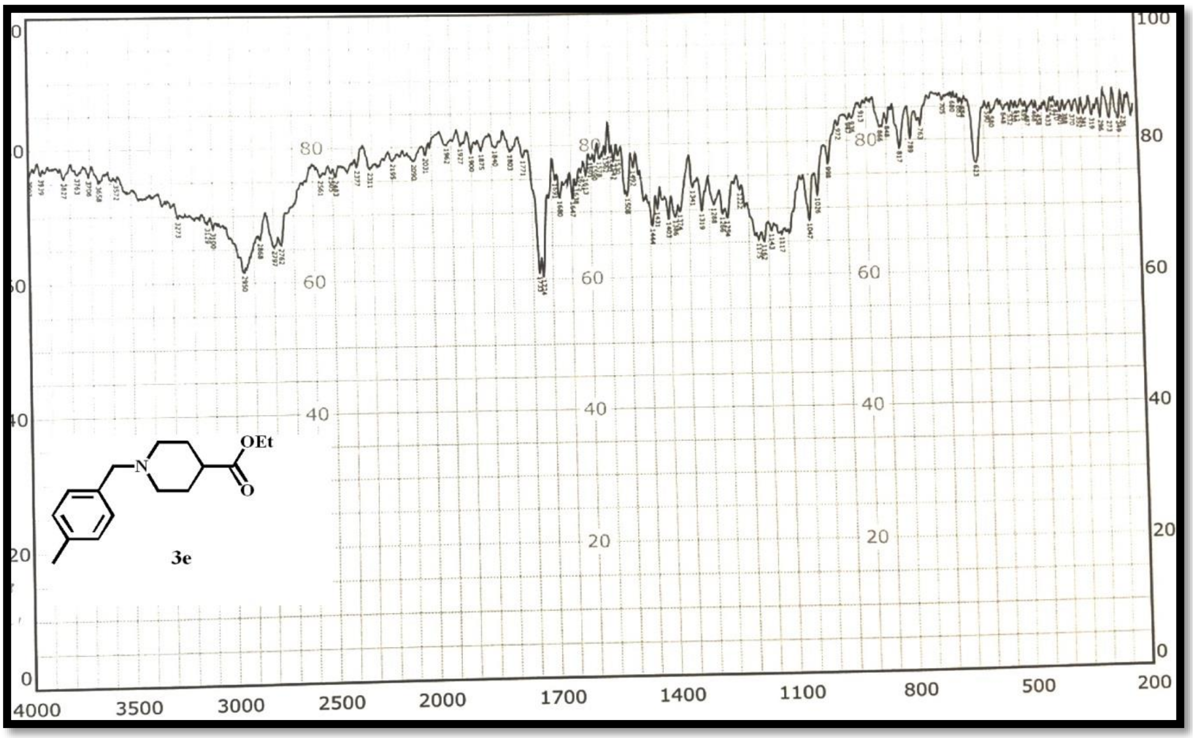

EXCLI Journal 2021;20:907-921 – ISSN 1611-2156
Received: February 27, 2021, accepted: May 10, 2021, published: May 18, 2021

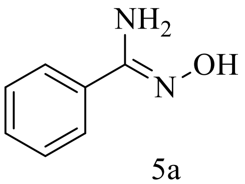

5a

N'-hydroxybenzamidine (5a)
Light yellow powder (96.5 % yield); mp: 68-70 °C; IR (KBr, cm^{-1}): 1657 (C=N), 3349, 3468 (NH_2); LC-MS $[M + 1]^+$: m/z 136.9.

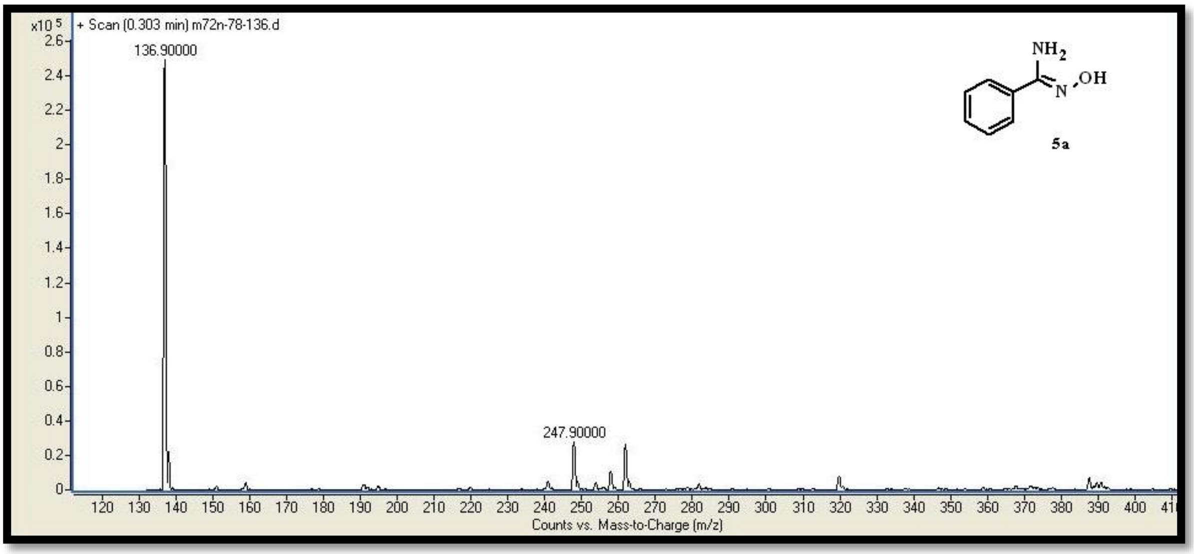

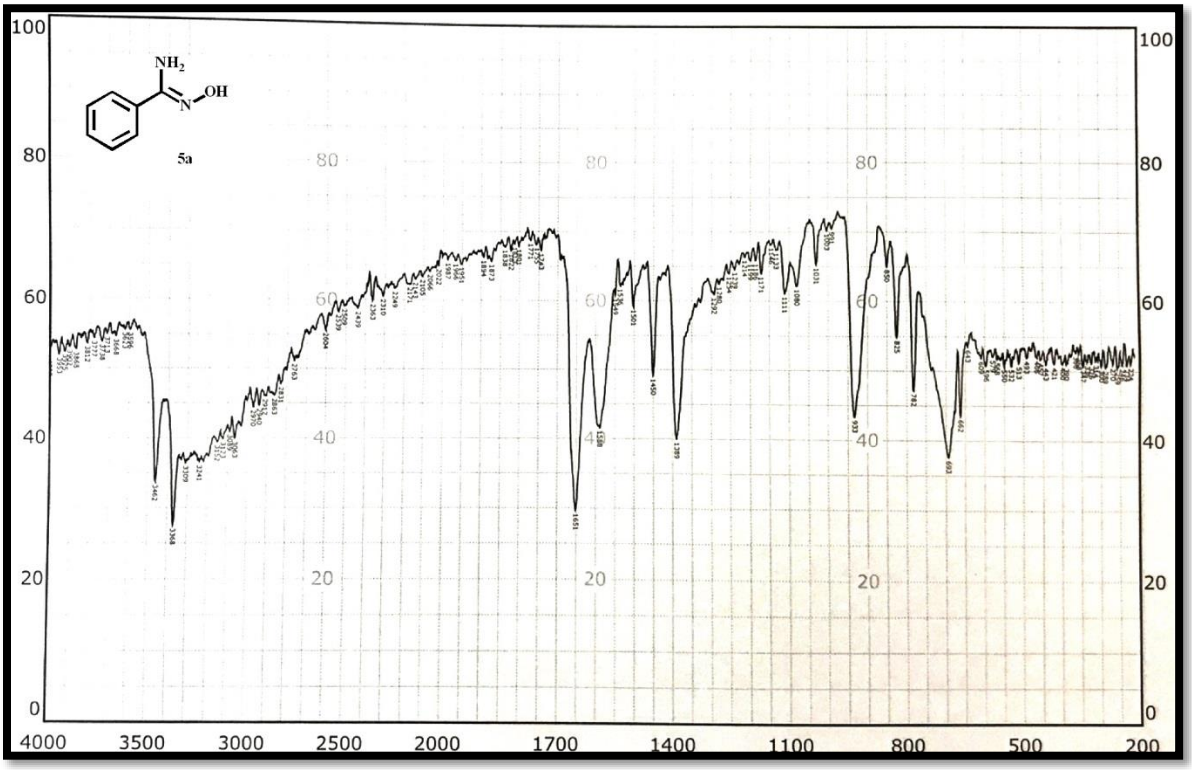

EXCLI Journal 2021;20:907-921 – ISSN 1611-2156
Received: February 27, 2021, accepted: May 10, 2021, published: May 18, 2021

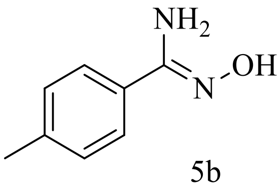

5b

N'-hydroxy-4-methylbenzamidine (5b)
Light yellow powder (81.9 % yield); mp: 145.8-148 °C; IR (KBr, cm^{-1}): 1661 (C=N), 3365, 3493 (NH_2); LC-MS [M + 1]$^+$: m/z 150.9.

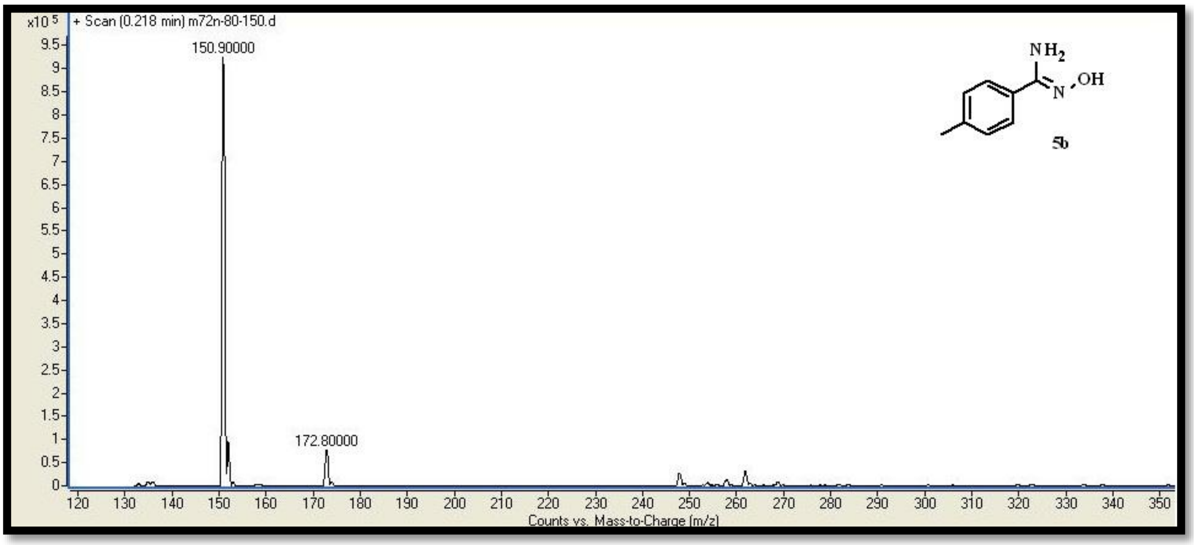

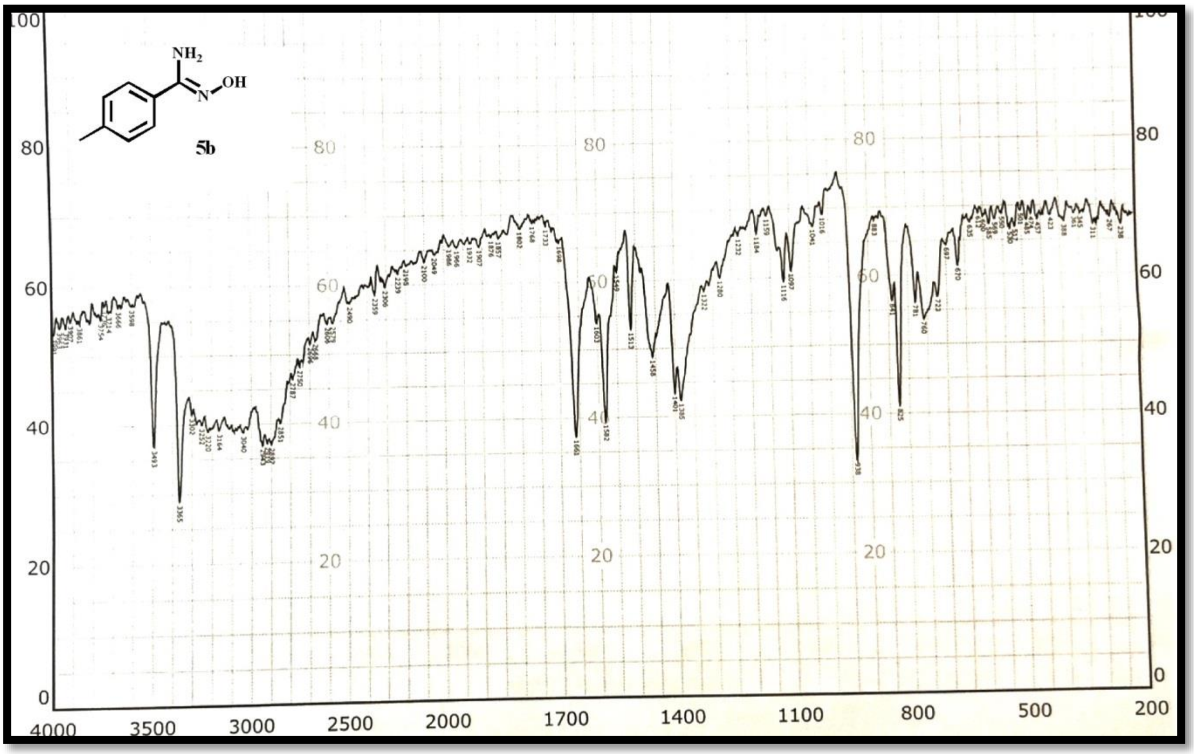

EXCLI Journal 2021;20:907-921 – ISSN 1611-2156
Received: February 27, 2021, accepted: May 10, 2021, published: May 18, 2021

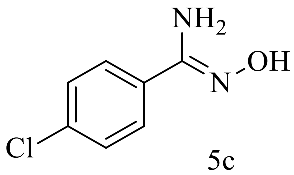

5c

4-Chloro-N′-hydroxy benzamidine (5c)
Light yellow powder (87.5 % yield); mp: 125.8-128 °C; IR (KBr, cm^{-1}): 1655 (C=N), 3337, 3462 (NH_2); LC-MS $[M + 1]^+$: m/z 170.8.

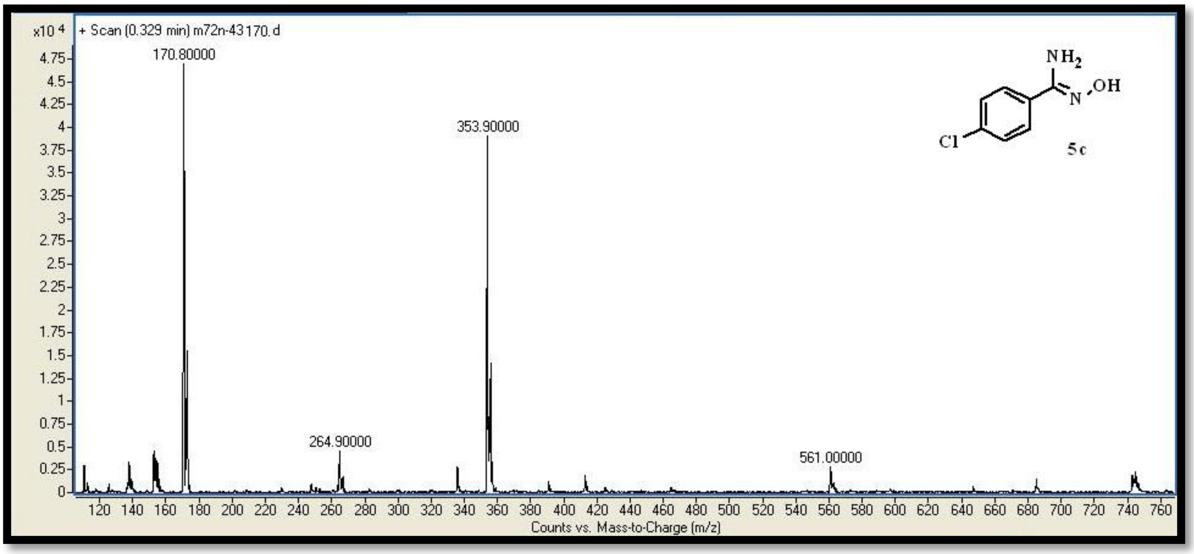

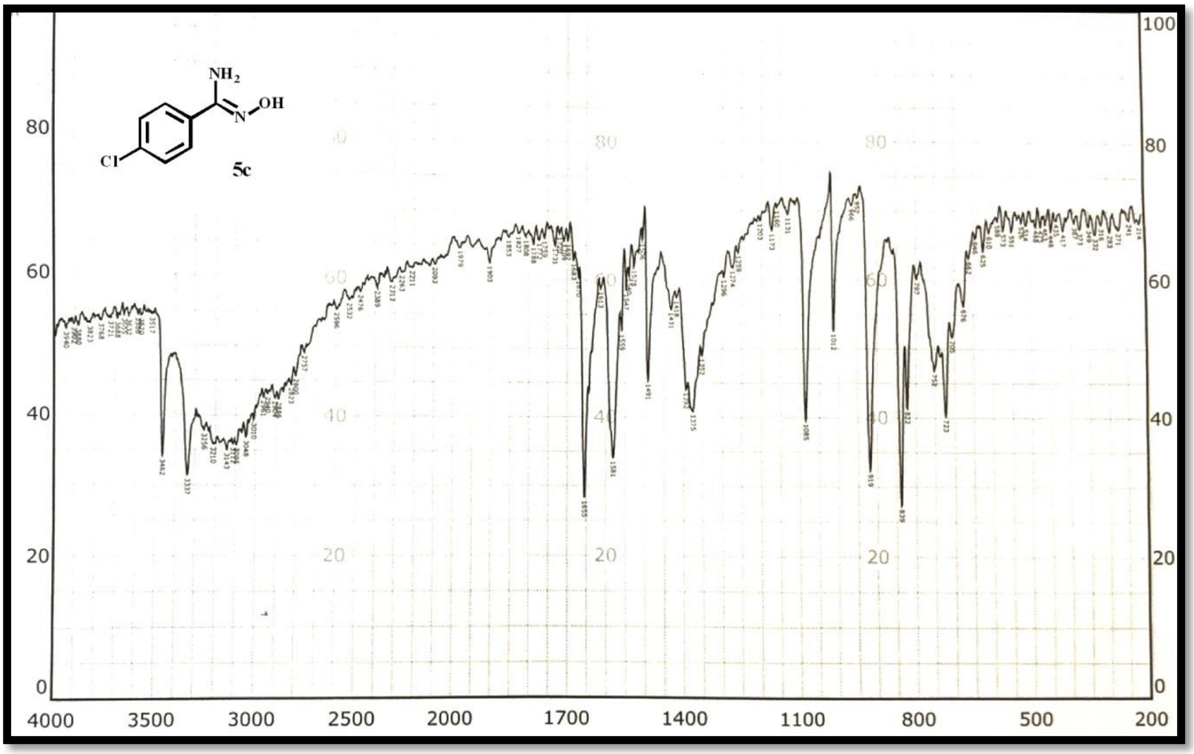

EXCLI Journal 2021;20:907-921 – ISSN 1611-2156
Received: February 27, 2021, accepted: May 10, 2021, published: May 18, 2021

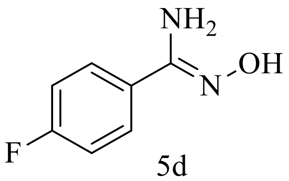

5d

4-Fluoro-N′-hydroxy benzamidine (5d)
Light yellow powder (68.2 % yield); mp: 94.5-99.5 °C; IR (KBr, cm[-1]): 1653 (C=N), 3363, 3457 (NH_2); LC-MS $[M + 1]^+$: m/z 154.9.

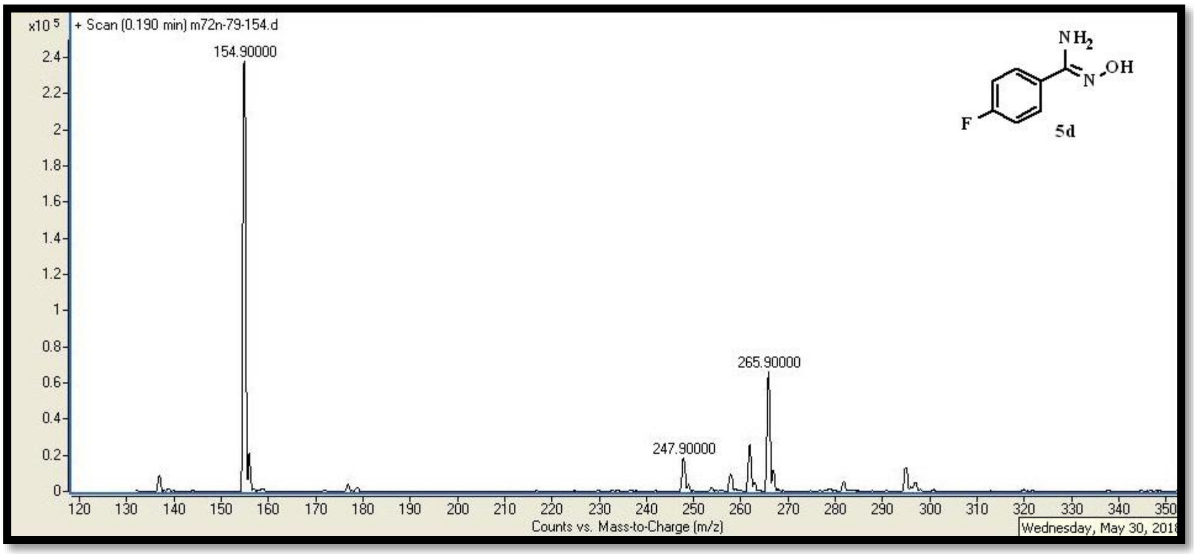

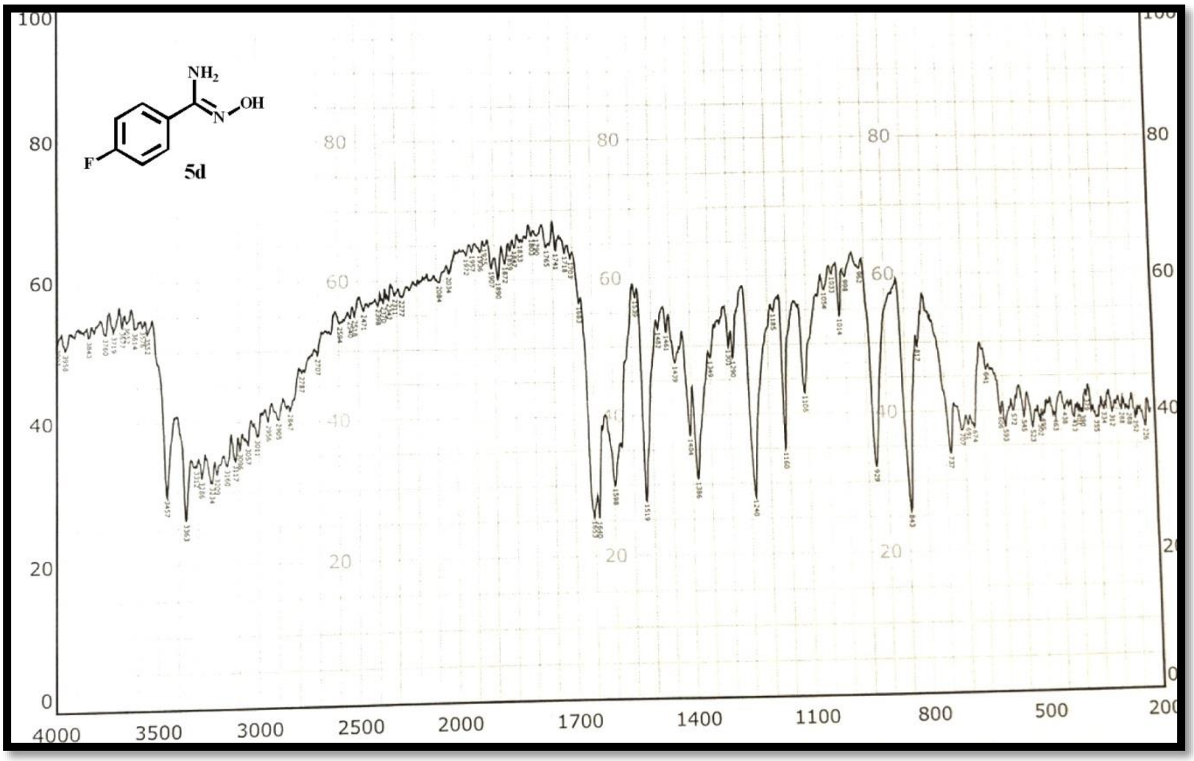

EXCLI Journal 2021;20:907-921 – ISSN 1611-2156
Received: February 27, 2021, accepted: May 10, 2021, published: May 18, 2021

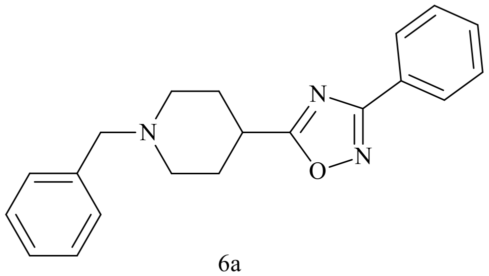

6a

5-(1-benzylpiperidin-4-yl)-3-phenyl-1,2,4-oxadiazole (6a)

Light yellow powder; yield: 54.4 %; mp: 70.8-71.2 °C; IR (KBr, cm^{-1}): 1587 (C=N), 1142 (C-O); LC-MS $[M + 1]^+$: m/z 320; ^1H NMR ($CDCl_3$, 400 MHz) δ: 2.02-2.20 (m, 6H, H-piperidine), 2.94-3.03 (m, 3H, H-piperidine), 3.54 (s, 2H, CH_2-benzyl), 7.25-7.34 (m, 5H, H_2, H_3, H_4, H_5, H_6-benzyl), 7.47-7.48 (m, 3H, H_3, H_4, H_5-phenyl), 8.07-8.08 (m, 2H, H_2, H_6-phenyl); ^{13}C NMR ($CDCl_3$, 100 MHz) δ: 29.56 ($2CH_2$), 34.64 (CH), 52.75 ($2CH_2$), 63.20 (CH_2), 126.99 (C), 127.07 (CH), 127.42 (2CH), 128.25 (2CH), 128.80 (2CH), 129.04 (2CH), 131.05 (CH), 138.29 (C), 168.19 (C), 182.07 (C); Anal. calcd for $C_{20}H_{21}N_3O$: C, 75.21; H, 6.63; N, 13.16, found: C, 75.43; H, 6.61; N, 13.13.

EXCLI Journal 2021;20:907-921 – ISSN 1611-2156
Received: February 27, 2021, accepted: May 10, 2021, published: May 18, 2021

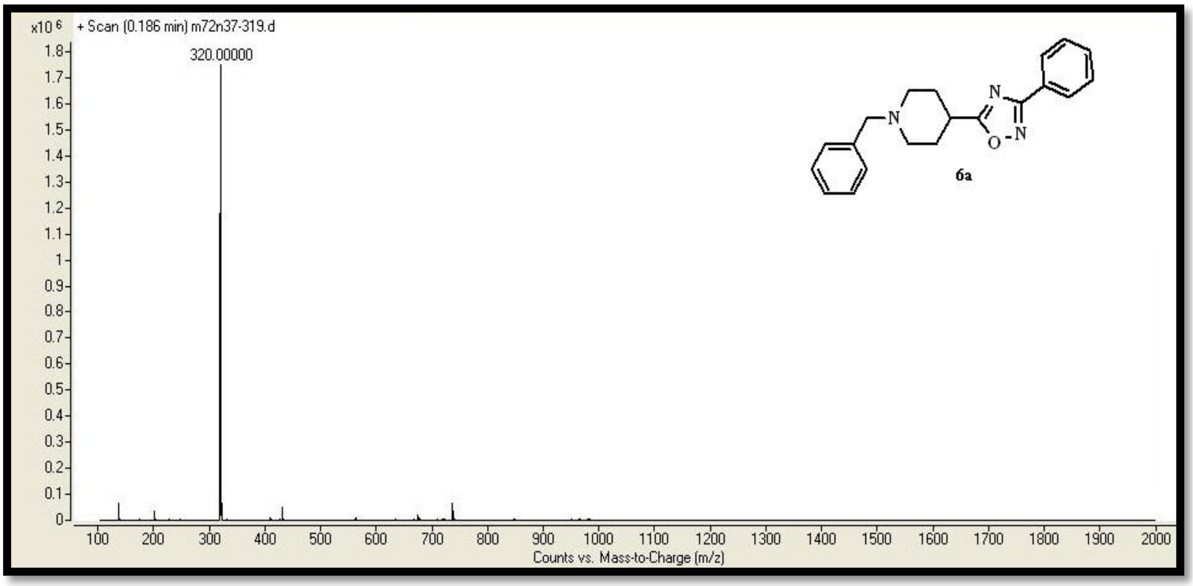

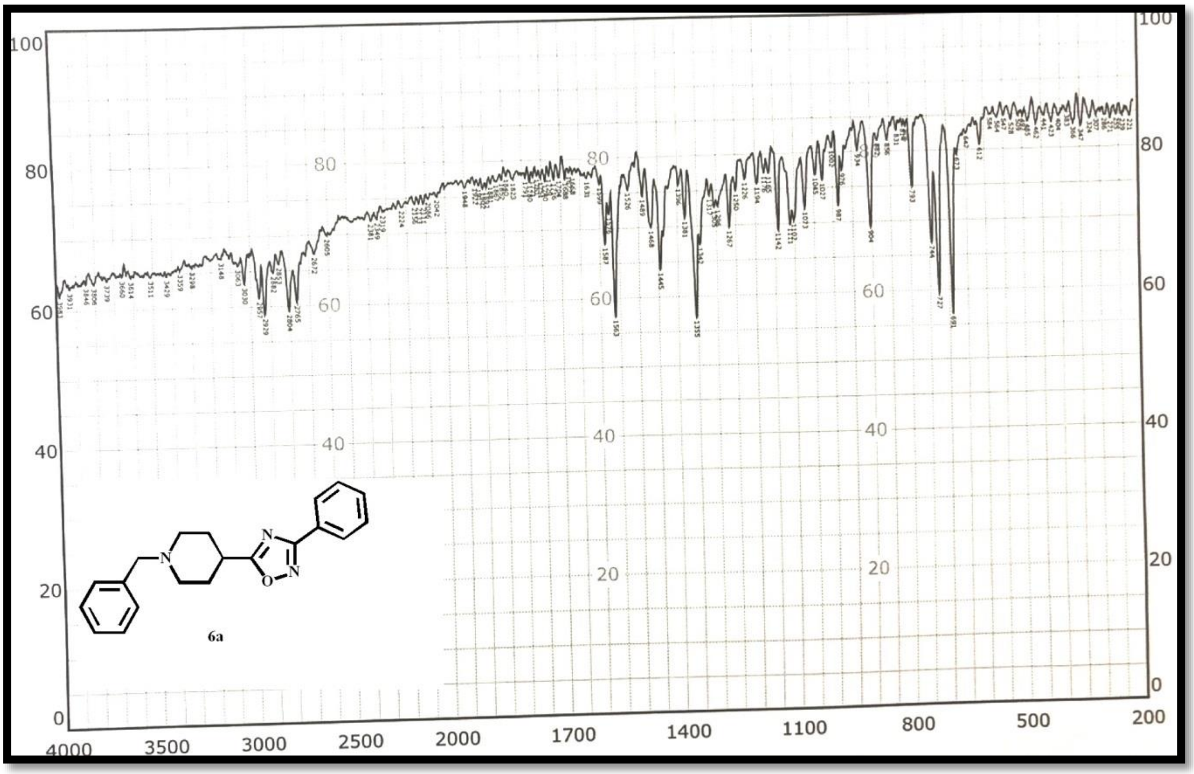

EXCLI Journal 2021;20:907-921 – ISSN 1611-2156
Received: February 27, 2021, accepted: May 10, 2021, published: May 18, 2021

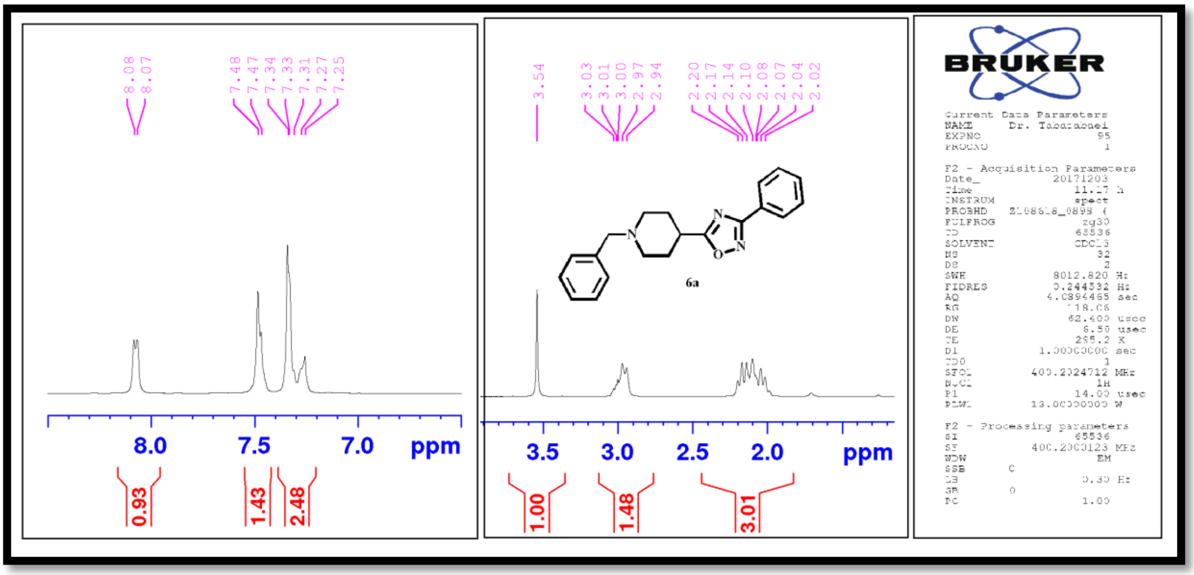

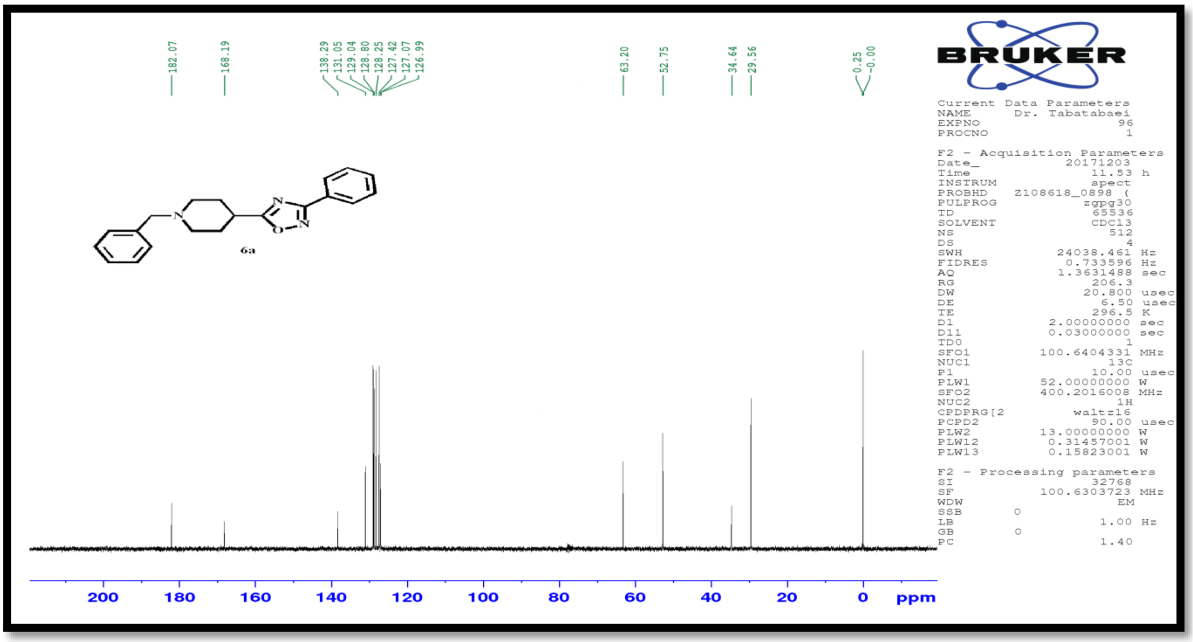

EXCLI Journal 2021;20:907-921 – ISSN 1611-2156
Received: February 27, 2021, accepted: May 10, 2021, published: May 18, 2021

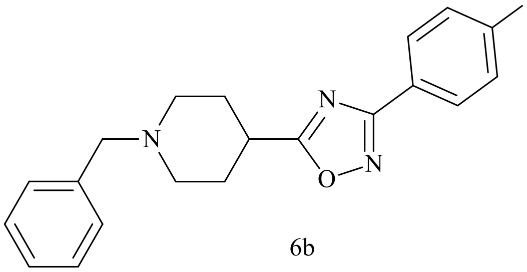

6b

5-(1-benzylpiperidin-4-yl)-3-(p-tolyl)-1,2,4-oxadiazole (6b)

Light yellow powder; yield: 32.9 %; mp: 88.5-89.8 °C; IR (KBr, cm^{-1}): 1582 (C=N), 1145 (C-O), 1346,1440 (CH_3); LC-MS $[M + 1]^+$: m/z 333.9; ^1H NMR ($CDCl_3$, 400 MHz) δ: 2.00-2.18 (m, 6H, H-piperidine), 2.40 (s, 3H, CH_3), 2.93-3.01 (m, 3H, H-piperidine), 3.53 (s, 2H, CH_2-benzyl), 7.26-7.28 (m, 2H, H_3, H_5-phenyl), 7.32-7.33 (m, 5H, H_2, H_3, H_4, H_5, H_6-benzyl), 7.95-7.97 (m, 2H, H_2, H_6-phenyl); ^{13}C NMR ($CDCl_3$, 100 MHz) δ: 21.55 (CH_3), 29.54 ($2CH_2$), 34.62 (CH), 52.74 ($2CH_2$), 63.18 (CH_2), 124.13 (C), 127.04 (2CH), 127.31 (CH), 128.23 (2CH), 129.02 (2CH), 129.49 (2CH), 138.28 (C), 141.31 (C), 168.15 (C), 181.86 (C); Anal. calcd for $C_{21}H_{23}N_3O$: C, 75.65; H, 6.95; N, 12.60, found: C, 75.87; H, 6.92; N, 12.56.

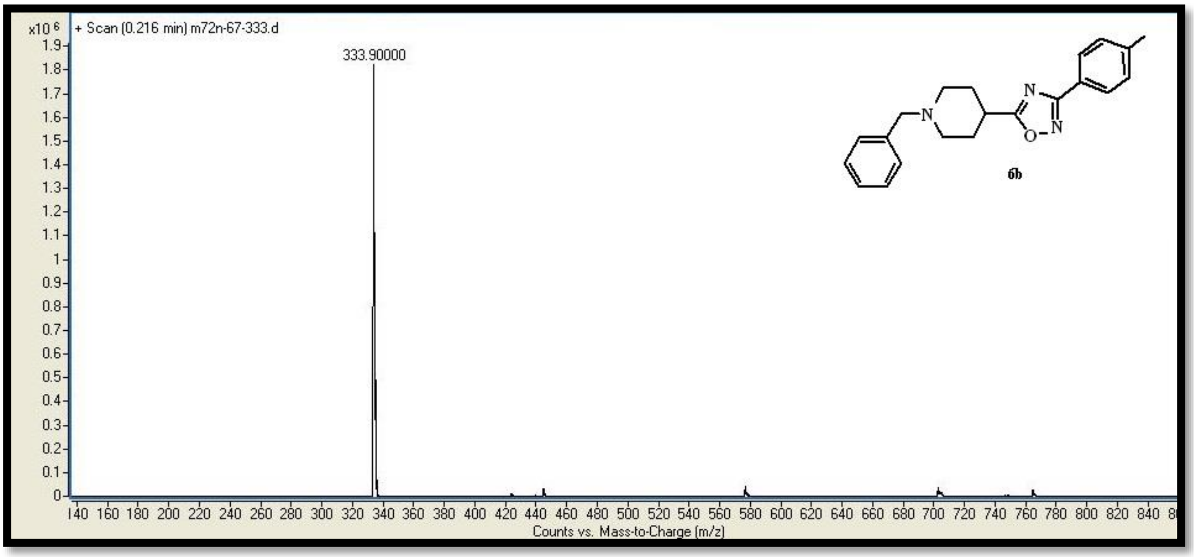
x10 6
+ Scan (0.216 min) m72n-67-333.d
333.90000
6b
Counts vs. Mass-to-Charge (m/z)

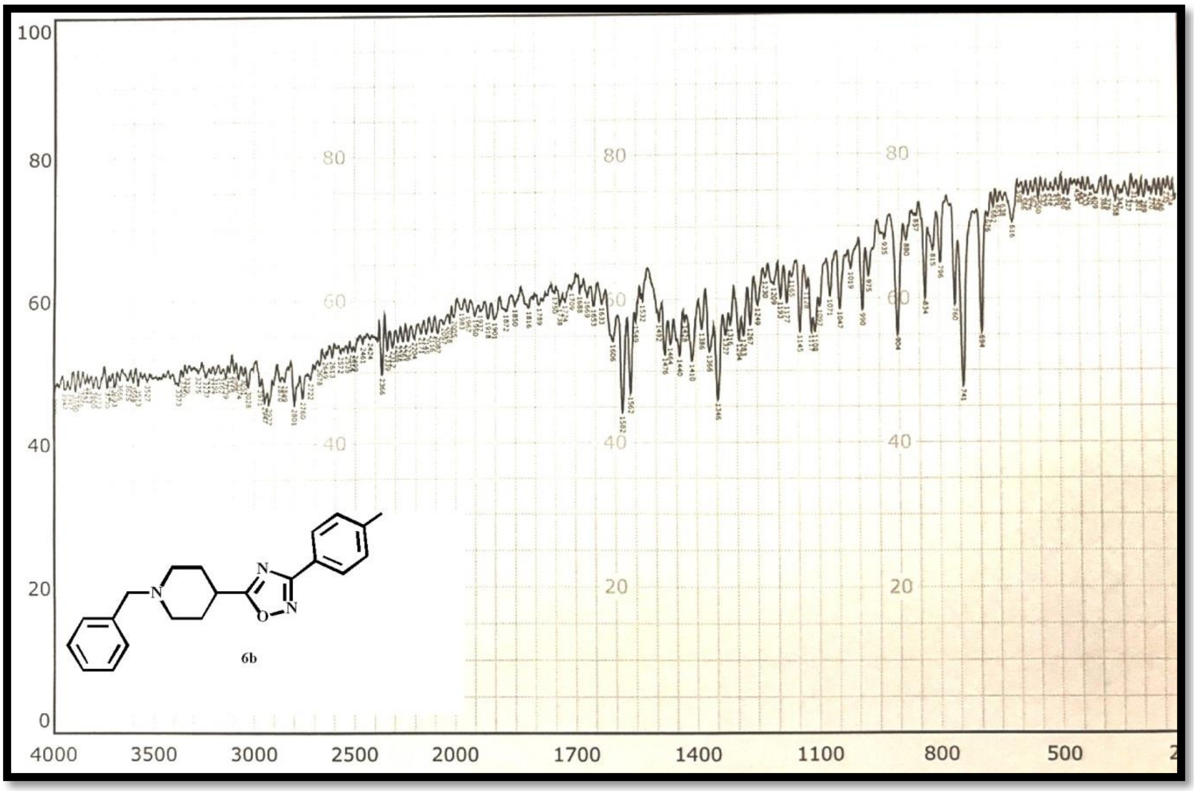
6b

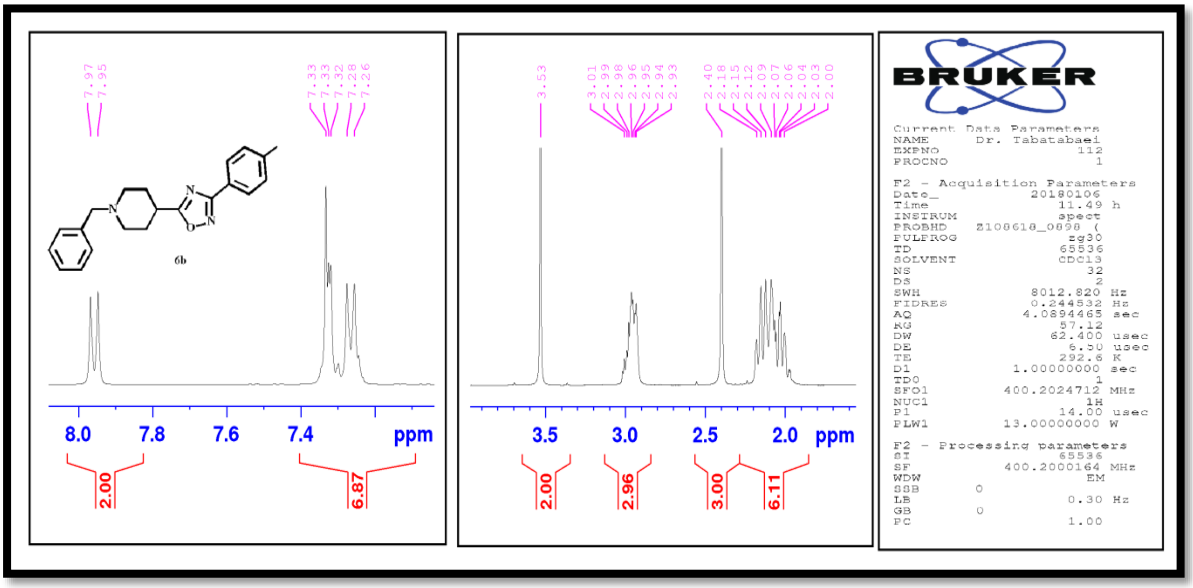

7.97
7.95
7.33
7.33
7.32
7.28
7.26
3.53
3.01
2.99
2.98
2.96
2.95
2.94
2.93
2.40
2.18
2.15
2.12
2.09
2.07
2.06
2.04
2.03
2.00
N
N
O
N
6b
8.0
7.8
7.6
7.4
ppm
3.5
3.0
2.5
2.0
ppm
2.00
6.87
2.00
2.96
3.00
6.11
BRUKER
Current Data Parameters
NAME Dr. Tabatabaei
EXPNO 112
PROCNO 1
F2 - Acquisition Parameters
Date_ 20180106
Time 11.49 h
INSTRUM spect
PROBHD Z108618_0898 (
PULPROG zg30
TD 65536
SOLVENT CDCl3
NS 32
DS 2
SWH 8012.820 Hz
FIDRES 0.244532 Hz
AQ 4.0894465 sec
RG 57.12
DW 62.400 usec
DE 6.50 usec
TE 292.6 K
D1 1.00000000 sec
TD0 1
SFO1 400.2024712 MHz
NUC1 1H
P1 14.00 usec
PLW1 13.00000000 W
F2 - Processing parameters
SI 65536
SF 400.2000164 MHz
WDW EM
SSB 0
LB 0.30 Hz
GB 0
PC 1.00

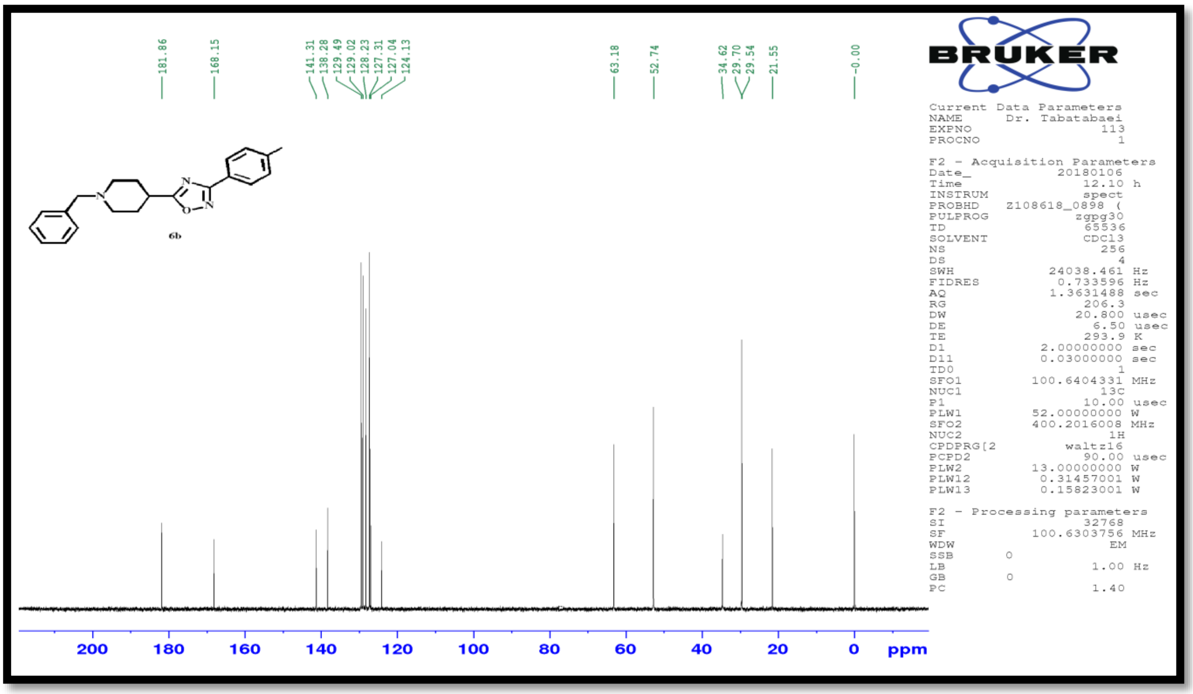

181.86
168.15
141.31
138.28
129.49
129.02
128.23
127.31
127.04
124.13
63.18
52.74
34.62
29.70
29.54
21.55
-0.00
N
N
O
N
6b
200
180
160
140
120
100
80
60
40
20
0
ppm
BRUKER
Current Data Parameters
NAME Dr. Tabatabaei
EXPNO 113
PROCNO 1
F2 - Acquisition Parameters
Date_ 20180106
Time 12.10 h
INSTRUM spect
PROBHD Z108618_0898 (
PULPROG zgpg30
TD 65536
SOLVENT CDCl3
NS 256
DS 4
SWH 24038.461 Hz
FIDRES 0.733596 Hz
AQ 1.3631488 sec
RG 206.3
DW 20.800 usec
DE 6.50 usec
TE 293.9 K
D1 2.00000000 sec
D11 0.03000000 sec
TD0 1
SFO1 100.6404331 MHz
NUC1 13C
P1 10.00 usec
PLW1 52.00000000 W
SFO2 400.2016008 MHz
NUC2 1H
CPDPRG[2 waltz16
PCPD2 90.00 usec
PLW2 13.00000000 W
PLW12 0.31457001 W
PLW13 0.15823001 W
F2 - Processing parameters
SI 32768
SF 100.6303756 MHz
WDW EM
SSB 0
LB 1.00 Hz
GB 0
PC 1.40

EXCLI Journal 2021;20:907-921 – ISSN 1611-2156
Received: February 27, 2021, accepted: May 10, 2021, published: May 18, 2021

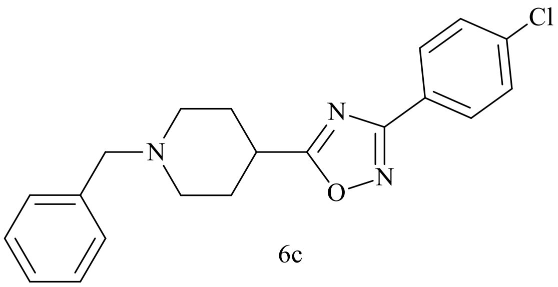

6c

5-(1-benzylpiperidin-4-yl)-3-(4-chlorophenyl)-1,2,4-oxadiazole (6c)

Light yellow powder; yield: 32.1 %; mp: 106-108 °C; IR (KBr, cm^{-1}): 1592 (C=N), 1139 (C-O); LC-MS $[M + 1]^+$: m/z 354; ^1H NMR ($CDCl_3$, 400 MHz) δ: 2.00-2.19 (m, 6H, H-piperidine), 2.94-3.02 (m, 3H, H-piperidine), 3.54 (s, 2H, CH_2-benzyl), 7.26-7.34 (m, 5H, H_2, H_3, H_4, H_5, H_6-benzyl), 7.44-7.46 (m, 2H, H_3, H_5-phenyl), 8.00-8.02 (m, 2H, H_2, H_6-phenyl); ^{13}C NMR ($CDCl_3$, 100 MHz) δ: 29.53 ($2CH_2$), 34.61 (CH), 52.71 ($2CH_2$), 63.18 (CH_2), 125.47 (C), 127.08 (2CH), 128.75 (CH), 128.72 (2CH), 129.04 (2CH), 129.12 (2CH), 137.17 (C), 138.23 (C), 167.39 (C), 182.30 (C); Anal. calcd for $C_{20}H_{20}ClN_3O$: C, 67.89; H, 5.70; N, 11.88, found: C, 68.12; H, 5.69; N, 11.82.

EXCLI Journal 2021;20:907-921 – ISSN 1611-2156
Received: February 27, 2021, accepted: May 10, 2021, published: May 18, 2021

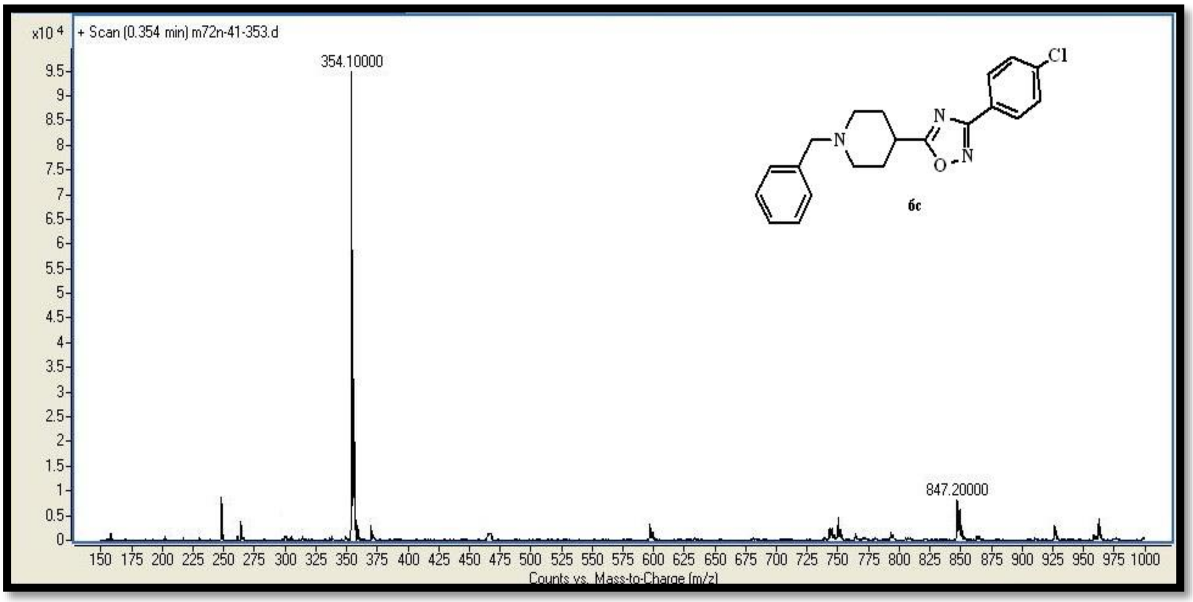

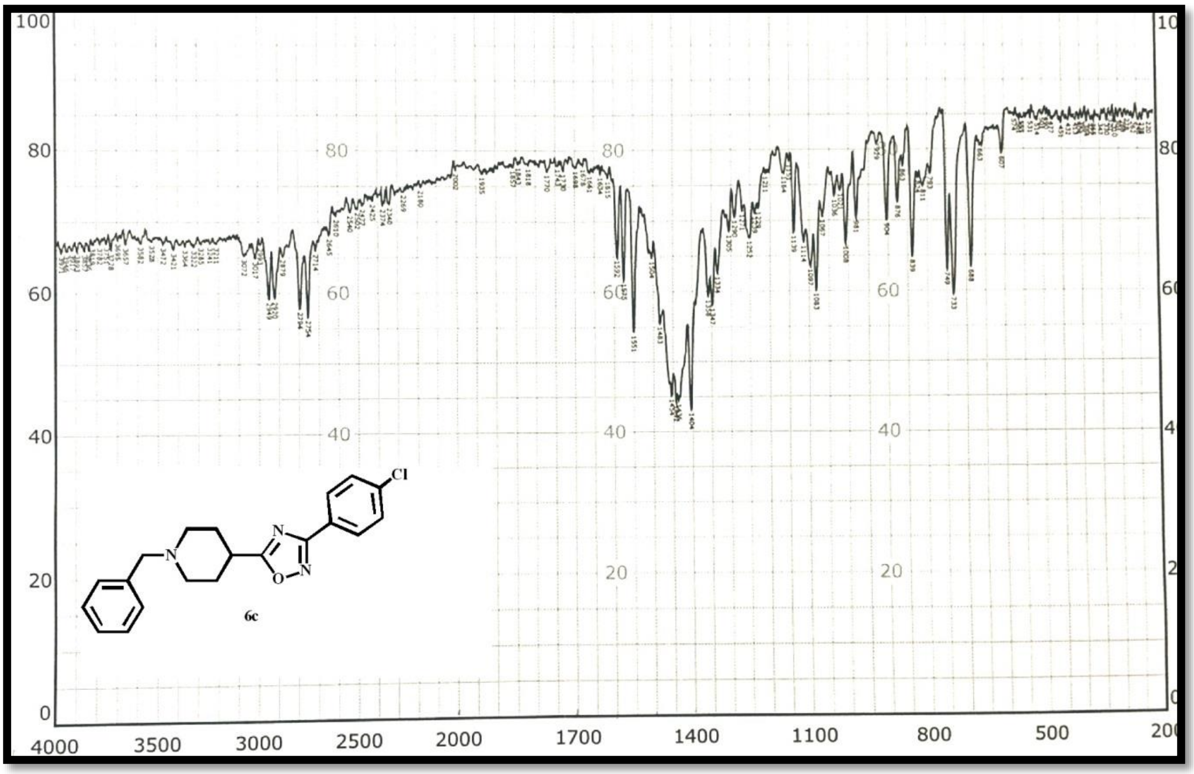

EXCLI Journal 2021;20:907-921 – ISSN 1611-2156
Received: February 27, 2021, accepted: May 10, 2021, published: May 18, 2021

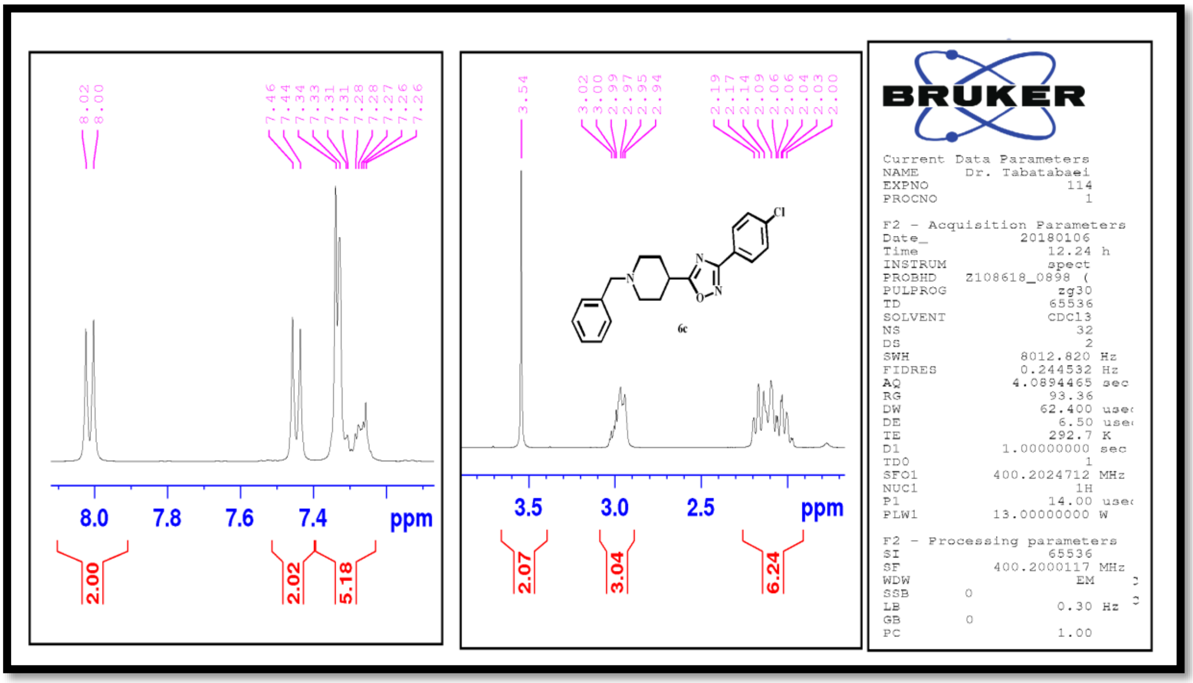

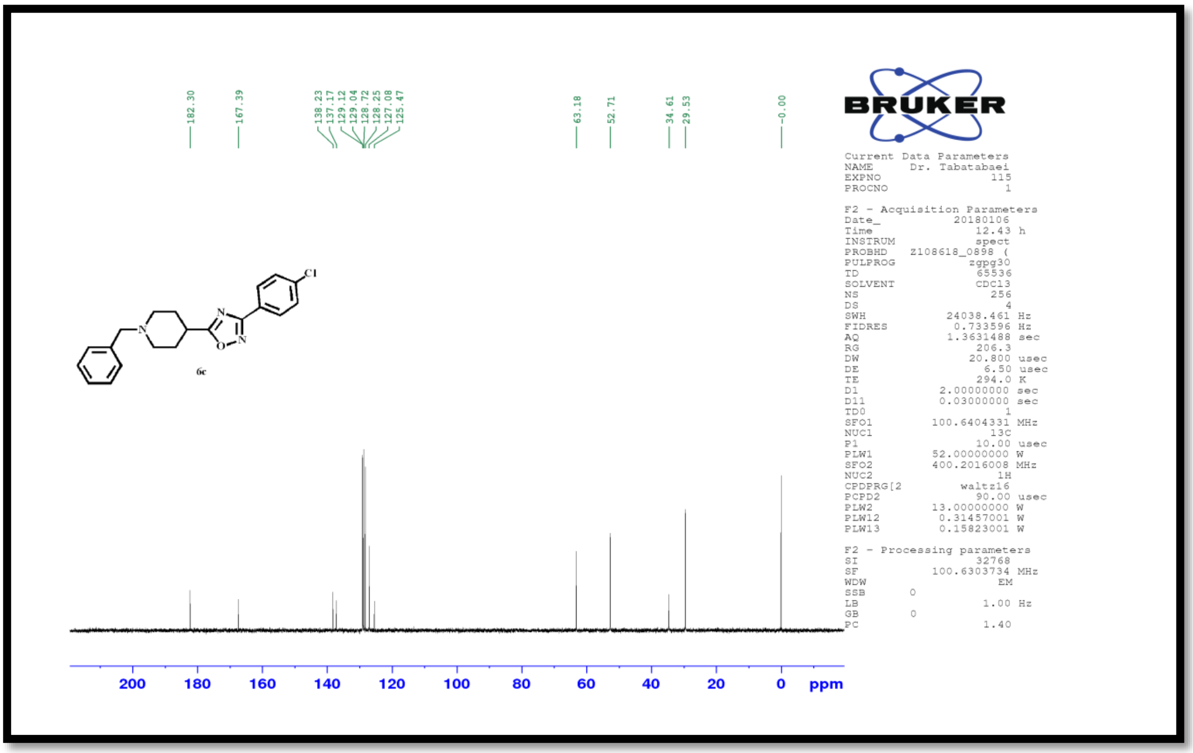

EXCLI Journal 2021;20:907-921 – ISSN 1611-2156
Received: February 27, 2021, accepted: May 10, 2021, published: May 18, 2021

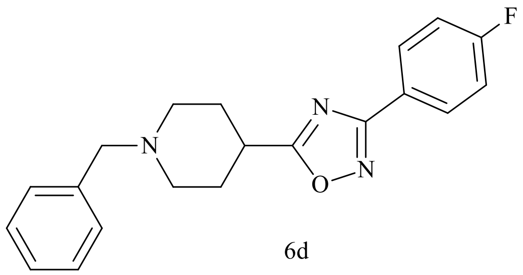

6d

5-(1-benzylpiperidin-4-yl)-3-(4-fluorophenyl)-1,2,4-oxadiazole (6d)

Light yellow powder; yield: 40.7 %; mp: 92.7-93.6 °C; IR (KBr, cm^{-1}): 1571 (C=N), 1130 (C-O); LC-MS $[M + 1]^+$: m/z 337.9; ^1H NMR ($CDCl_3$, 400 MHz) δ: 1.93-2.12 (m, 6H, H-piperidine), 2.87-2.93 (m, 3H, H-piperidine), 3.47 (s, 2H, CH_2-benzyl), 7.06-7.10 (m, 2H, H_3, H_5-phenyl), 7.18-7.26 (m, 5H, $H_{2,}$ H_3, $H_{4,}$ $H_{5,}$ H_6-benzyl), 7.98-8.01 (m, 2H, H_2, H_6-phenyl); ^{13}C NMR ($CDCl_3$, 100 MHz) δ: 29.55 ($2CH_2$), 34.61 (CH), 52.73 ($2CH_2$), 63.20 (CH_2), 115.90 (C), 123.23 (2CH), 127.12 (CH), 128.28 (2CH), 129.08 (2CH), 129.54 (2CH), 129.62 (C), 138.24 (C), 165.67 (C), 182.21 (C); Anal. calcd for $C_{20}H_{20}FN_3O$: C, 71.20; H, 5.97; N, 12.45, found: C, 71.41; H, 5.99; N, 12.40.

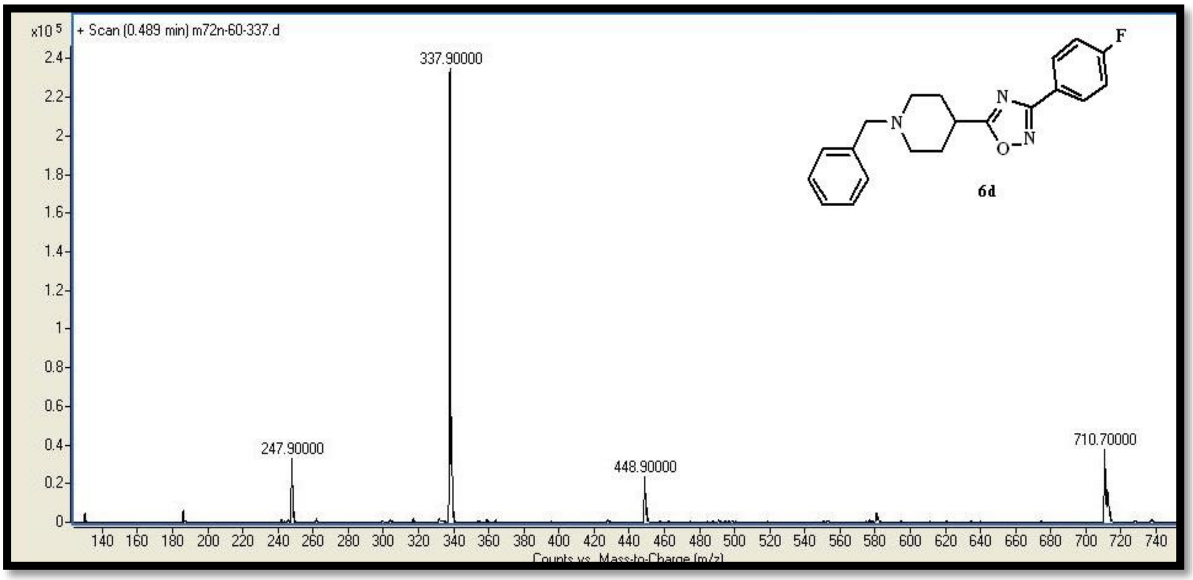
x10 5
+ Scan (0.489 min) m72n-60-337.d
337.90000
247.90000
448.90000
710.70000
6d
Counts vs. Mass-to-Charge (m/z)

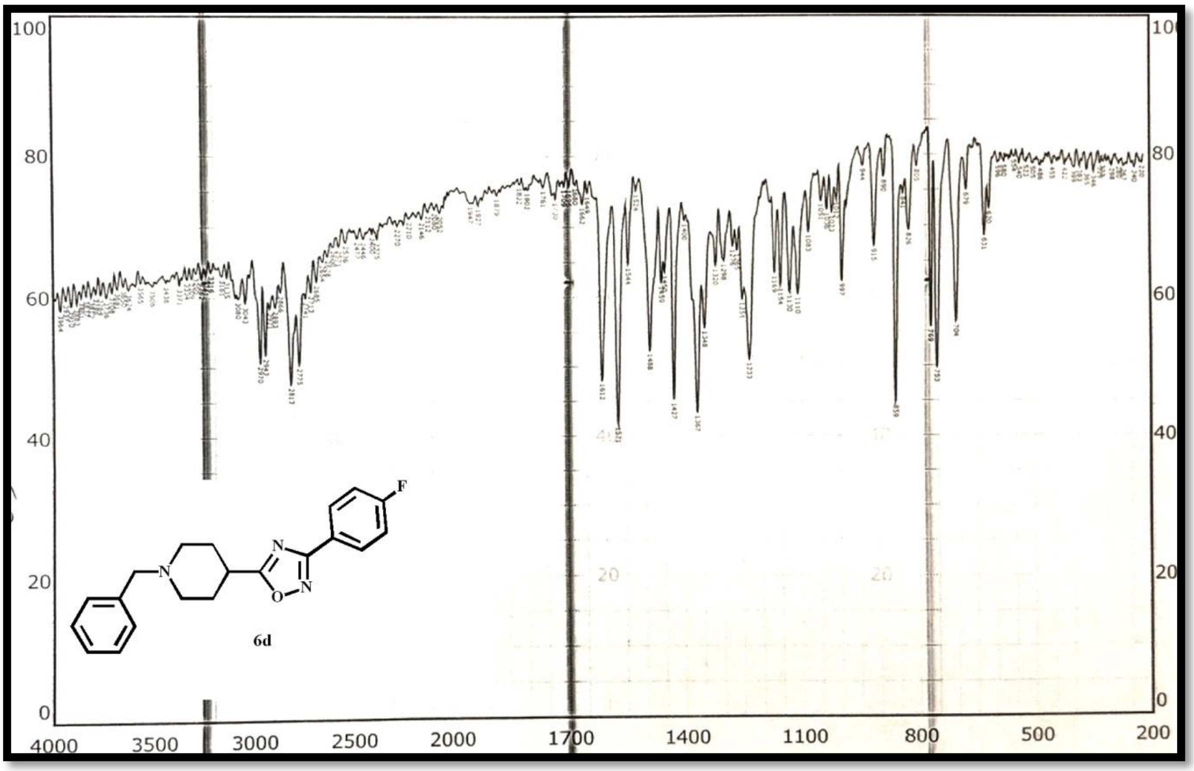
6d

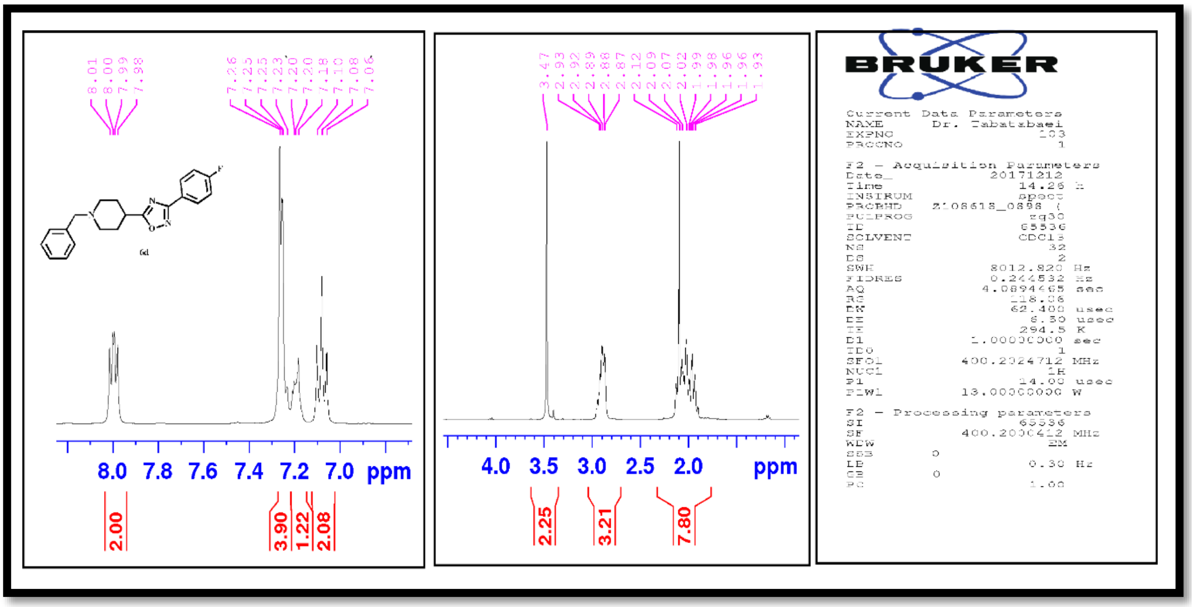
8.01
8.00
7.99
7.98
7.26
7.25
7.25
7.23
7.20
7.20
7.18
7.10
7.08
7.06
6d
8.0 7.8 7.6 7.4 7.2 7.0 ppm
2.00
3.90
1.22
2.08
3.47
2.93
2.92
2.89
2.88
2.87
2.12
2.09
2.07
2.02
1.99
1.98
1.96
1.96
1.93
4.0 3.5 3.0 2.5 2.0 ppm
2.25
3.21
7.80
BRUKER

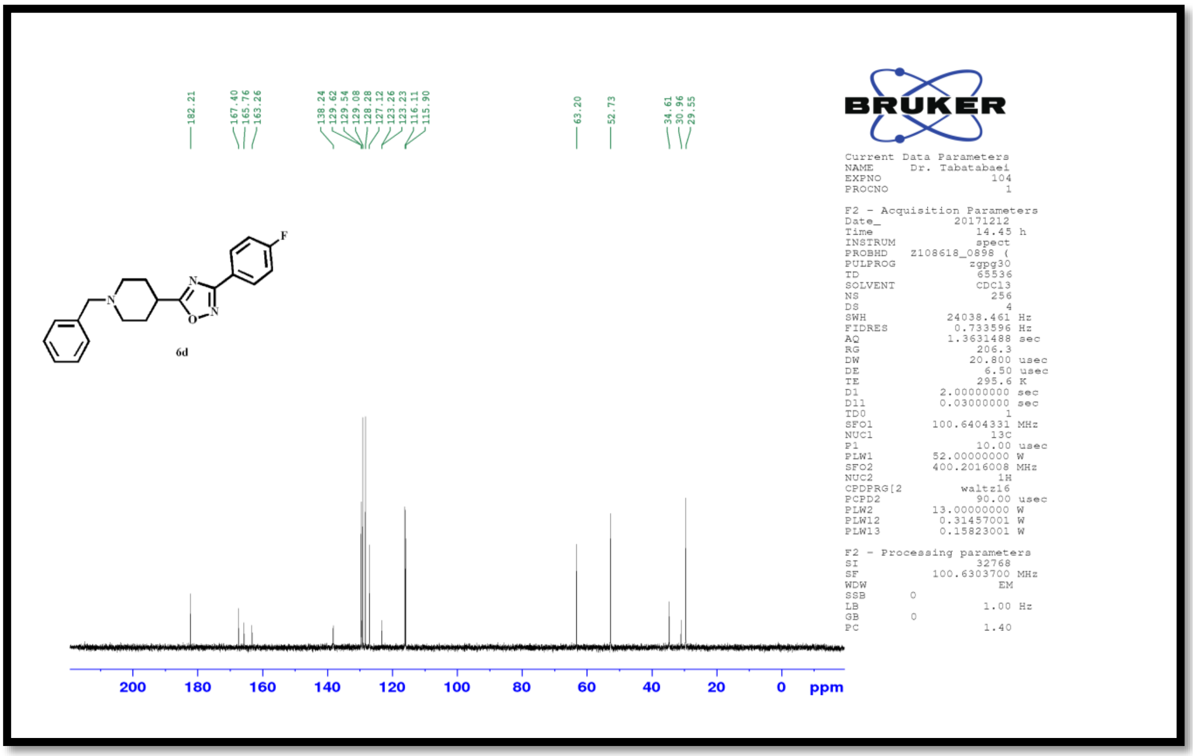
182.21
167.40
165.76
163.26
138.24
129.62
129.54
129.08
128.28
127.12
123.26
123.23
116.11
115.90
63.20
52.73
34.61
30.96
29.55
6d
200 180 160 140 120 100 80 60 40 20 0 ppm
BRUKER
Current Data Parameters
NAME Dr. Tabatabaei
EXPNO 104
PROCNO 1
F2 - Acquisition Parameters
Date_ 20171212
Time 14.45 h
INSTRUM spect
PROBHD Z108618_0898 (
PULPROG zgpg30
TD 65536
SOLVENT CDCl3
NS 256
DS 4
SWH 24038.461 Hz
FIDRES 0.733596 Hz
AQ 1.3631488 sec
RG 206.3
DW 20.800 usec
DE 6.50 usec
TE 295.6 K
D1 2.00000000 sec
D11 0.03000000 sec
TD0 1
SFO1 100.6404331 MHz
NUC1 13C
P1 10.00 usec
PLW1 52.00000000 W
SFO2 400.2016008 MHz
NUC2 1H
CPDPRG[2 waltz16
PCPD2 90.00 usec
PLW2 13.00000000 W
PLW12 0.31457001 W
PLW13 0.15823001 W
F2 - Processing parameters
SI 32768
SF 100.6303700 MHz
WDW EM
SSB 0
LB 1.00 Hz
GB 0
PC 1.40

EXCLI Journal 2021;20:907-921 – ISSN 1611-2156
Received: February 27, 2021, accepted: May 10, 2021, published: May 18, 2021

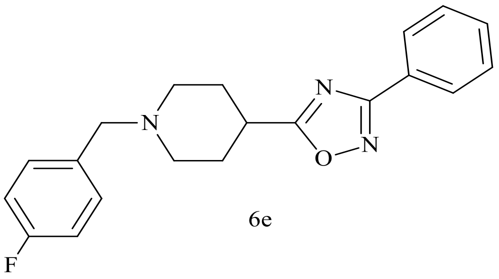

6e

5-(1-(4-fluorobenzyl)piperidin-4-yl)-3-phenyl-1,2,4-oxadiazole (6e)

Light yellow powder; yield: 20.9 %; mp: 93.4-94.5 °C; IR (KBr, cm^{-1}): 1596 (C=N), 1214 (C-O); LC-MS $[M + 1]^+$: m/z 338; ^1H NMR ($CDCl_3$, 400 MHz) δ: 1.97-2.07 (m, 4H, H-piperidine), 2.11-2.19 (m, 4H, H-piperidine), 2.92-3.05 (m, 1H, H-piperidine), 3.50 (s, 2H, CH_2-benzyl), 7.01 (t, 2H, J = 8 Hz, H_3, H_5-benzyl), 7.26-7.32 (m, 2H, H_2, H_6-benzyl), 7.45-7.50 (m, 3H, H_3, H_4, H_5-phenyl), 8.08 (d, 2H, J = 8 Hz, H_2, H_6-phenyl); ^{13}C NMR ($CDCl_3$, 100 MHz) δ: 29.53 ($2CH_2$), 34.60 (CH), 52.65 ($2CH_2$), 62.35 (CH_2), 115.15 (2CH), 126.95 (C), 127.42 (2CH), 128.81 (2CH), 130.40 (2CH), 131.08 (CH), 133.98 (C), 160.79 (C), 168.20 (C), 182.00 (C); Anal. calcd for $C_{20}H_{20}FN_3O$: C, 71.20; H, 5.97; N, 12.45, found: C, 71.40; H, 5.94; N, 12.48.

EXCLI Journal 2021;20:907-921 – ISSN 1611-2156
Received: February 27, 2021, accepted: May 10, 2021, published: May 18, 2021

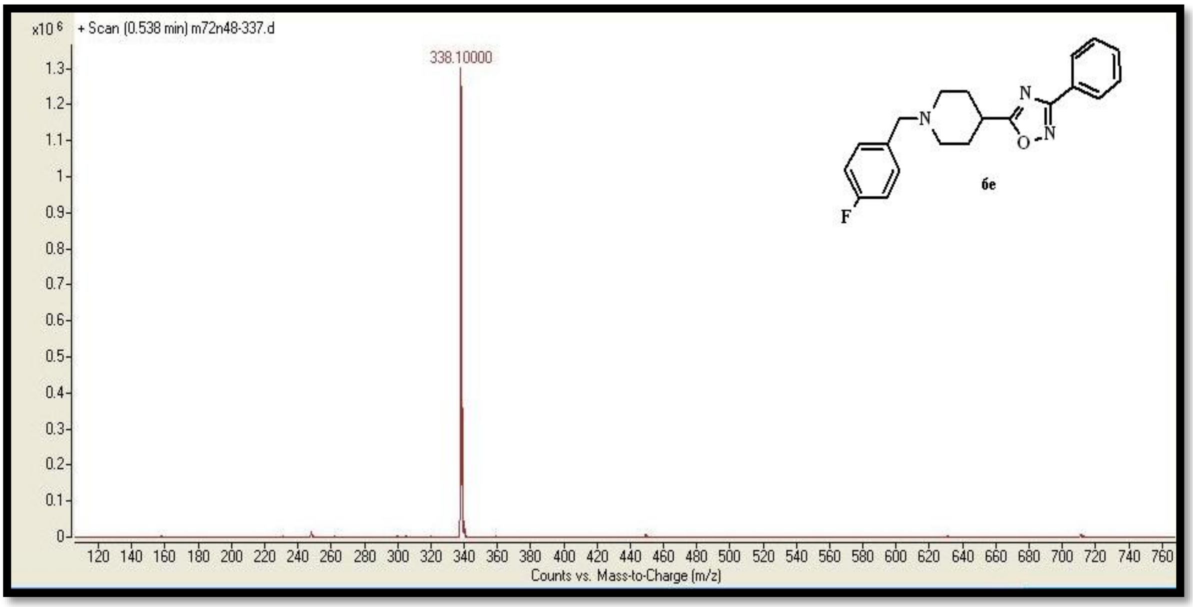

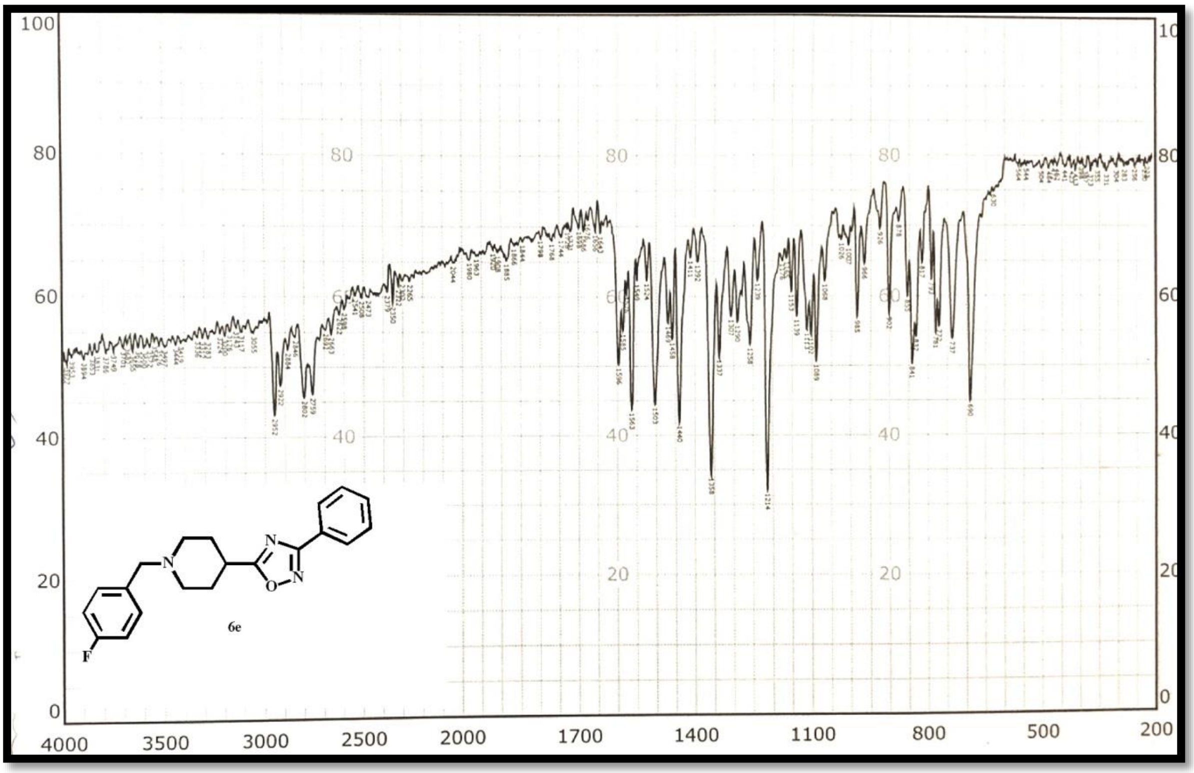

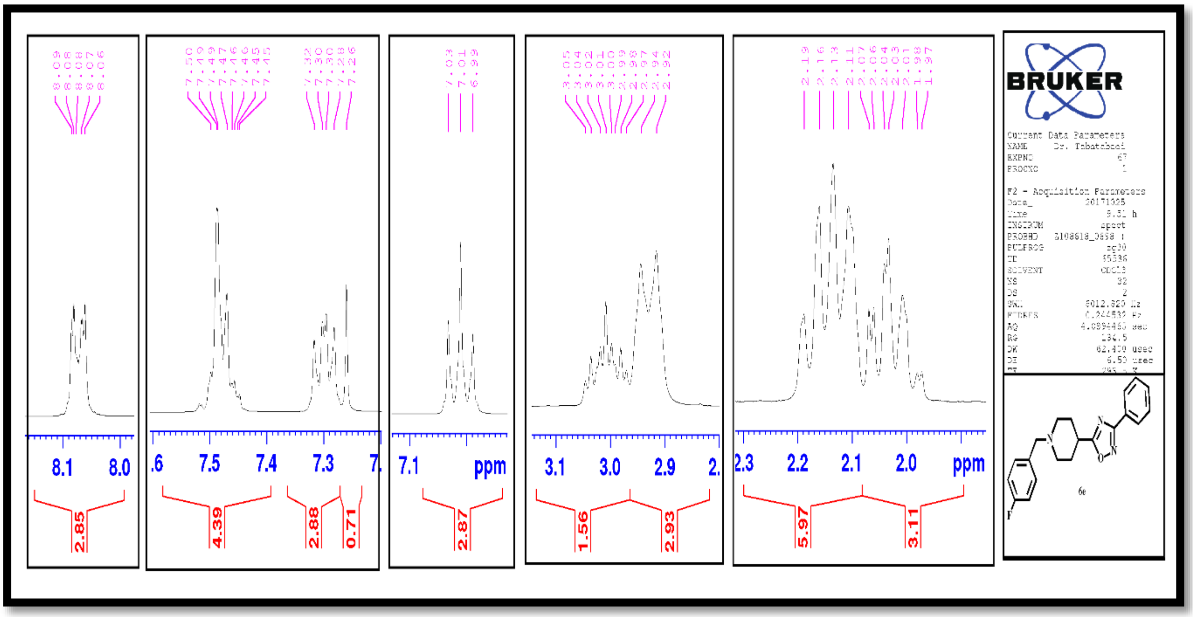
BRUKER
8.1
8.0
7.5
7.4
7.3
7.1
ppm
3.1
3.0
2.9
2.3
2.2
2.1
2.0
ppm
2.85
4.39
2.88
0.71
2.87
1.56
2.93
5.97
3.11
6e

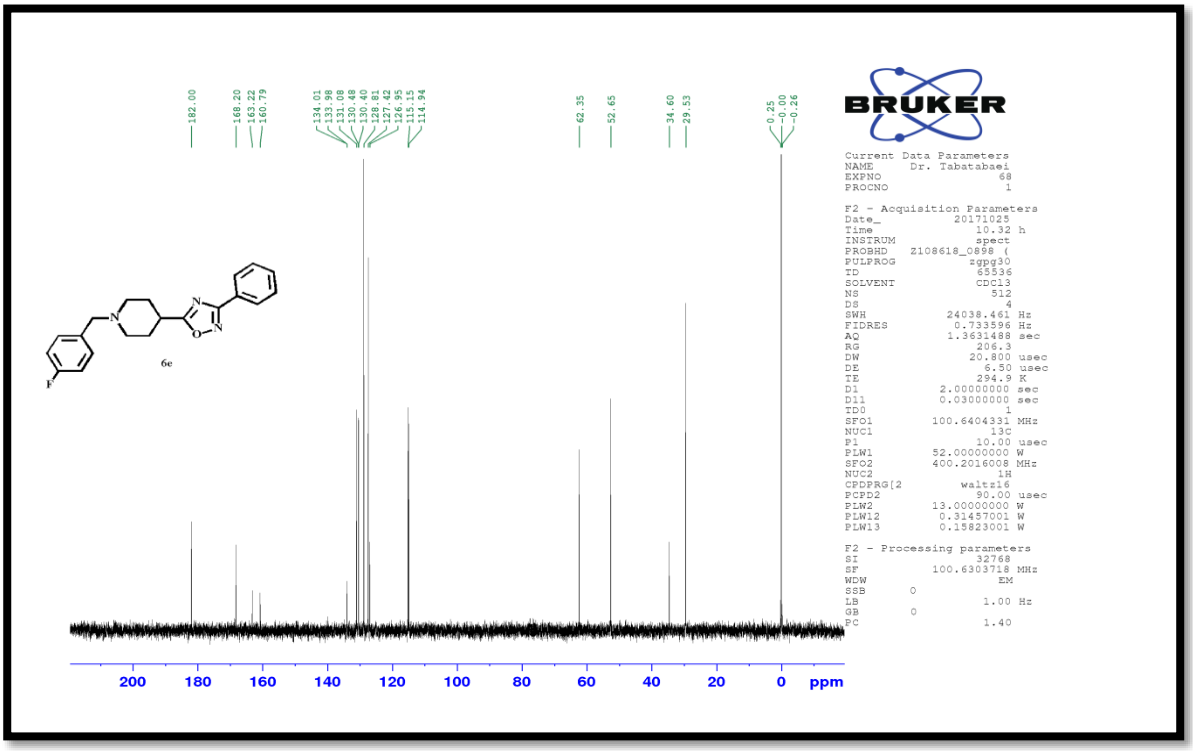
182.00
168.20
163.22
160.79
134.01
133.98
131.08
130.48
130.40
128.81
127.42
126.95
115.15
114.94
62.35
52.65
34.60
29.53
0.25
-0.00
-0.26
BRUKER
Current Data Parameters
NAME Dr. Tabatabaei
EXPNO 68
PROCNO 1
F2 - Acquisition Parameters
Date_ 20171025
Time 10.32 h
INSTRUM spect
PROBHD Z108618_0898 (
PULPROG zgpg30
TD 65536
SOLVENT CDCl3
NS 512
DS 4
SWH 24038.461 Hz
FIDRES 0.733596 Hz
AQ 1.3631488 sec
RG 206.3
DW 20.800 usec
DE 6.50 usec
TE 294.9 K
D1 2.00000000 sec
D11 0.03000000 sec
TD0 1
SFO1 100.6404331 MHz
NUC1 13C
P1 10.00 usec
PLW1 52.00000000 W
SFO2 400.2016008 MHz
NUC2 1H
CPDPRG[2 waltz16
PCPD2 90.00 usec
PLW2 13.00000000 W
PLW12 0.31457001 W
PLW13 0.15823001 W
F2 - Processing parameters
SI 32768
SF 100.6303718 MHz
WDW EM
SSB 0
LB 1.00 Hz
GB 0
PC 1.40
6e
200
180
160
140
120
100
80
60
40
20
0
ppm

EXCLI Journal 2021;20:907-921 – ISSN 1611-2156
Received: February 27, 2021, accepted: May 10, 2021, published: May 18, 2021

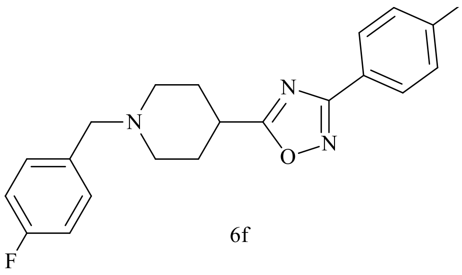

6f

5-(1-(4-fluorobenzyl)piperidin-4-yl)-3-(p-tolyl)-1,2,4-oxadiazole (6f)

White powder; yield: 56.3 %; mp: 90-90.8 °C; IR (KBr, cm^{-1}): 1582 (C=N), 1223 (C-O), 1344,1440 (CH_3); LC-MS $[M + 1]^+$: m/z 351.9; ^1H NMR ($CDCl_3$, 400 MHz) δ: 2.02-2.17 (m, 6H, H-piperidine), 2.40 (s, 3H, CH_3), 2.90-3.01 (m, 3H, H-piperidine), 3.49 (s, 2H, CH_2-benzyl), 6.98-7.03 (m, 2H, H_3, H_5-benzyl), 7.26-7.31 (m, 4H, H_2, H_6-benzyl, H_3, H_5-phenyl), 7.95 (d, 2H, J = 8 Hz, H_2, H_6-phenyl); ^{13}C NMR ($CDCl_3$, 100 MHz) δ: 21.55 (CH_3), 29.53 ($2CH_2$), 34.59 (CH), 52.65 ($2CH_2$), 62.34 (CH_2), 115.13 (2CH), 124.13 (C), 127.33 (2CH), 129.51 (2CH), 130.46 (2CH), 134.04 (CH), 141.35 (C), 160.77 (C), 168.18 (C), 181.81 (C); Anal. calcd for $C_{21}H_{22}FN_3O$: C, 71.77; H, 6.31; N, 11.96, found: C, 72.01; H, 6.32; N, 11.91.

EXCLI Journal 2021;20:907-921 – ISSN 1611-2156
Received: February 27, 2021, accepted: May 10, 2021, published: May 18, 2021

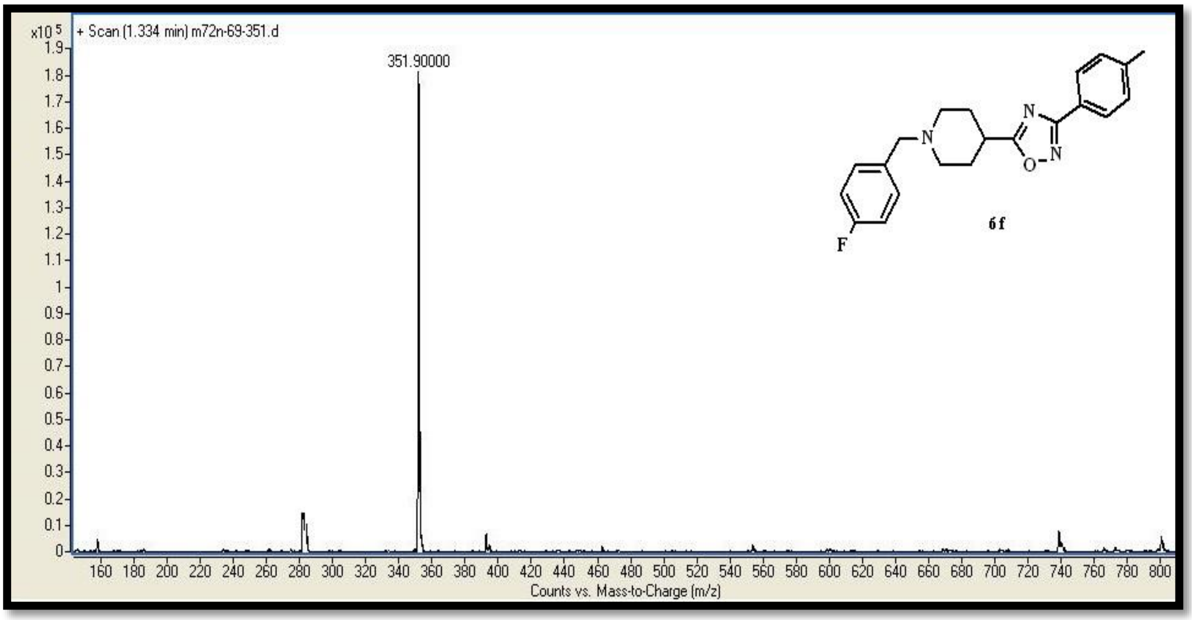

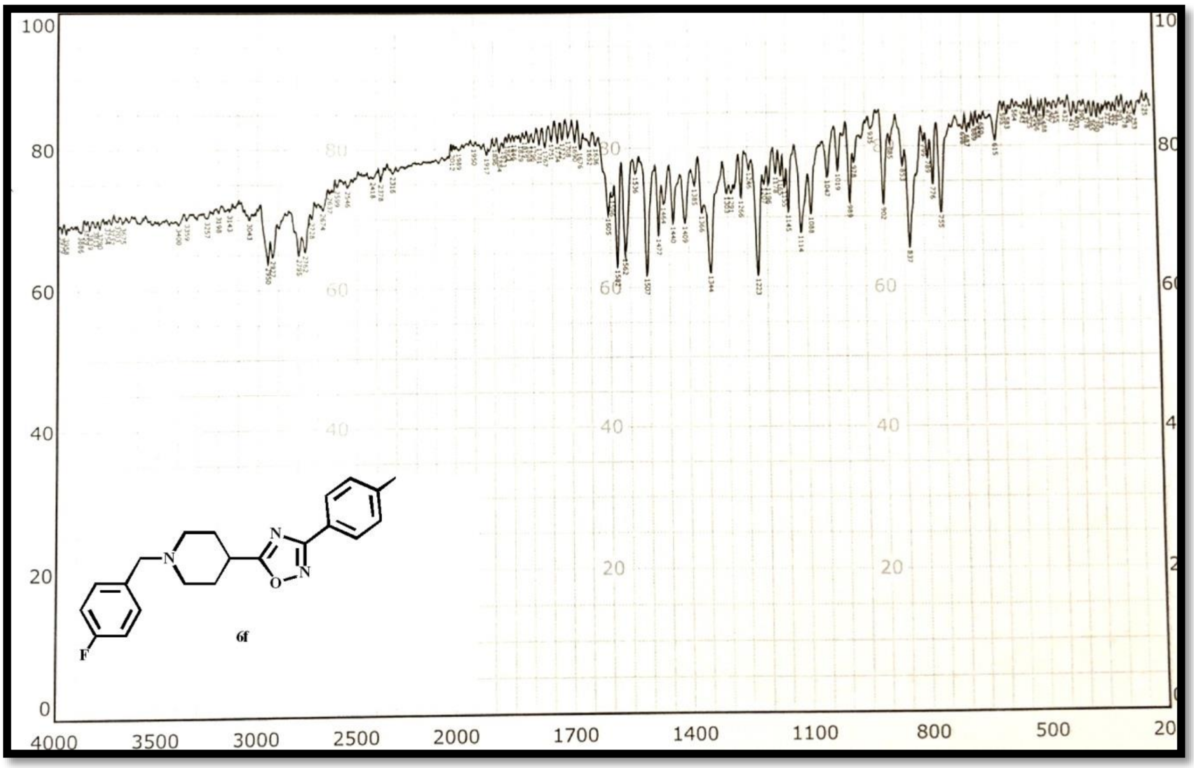

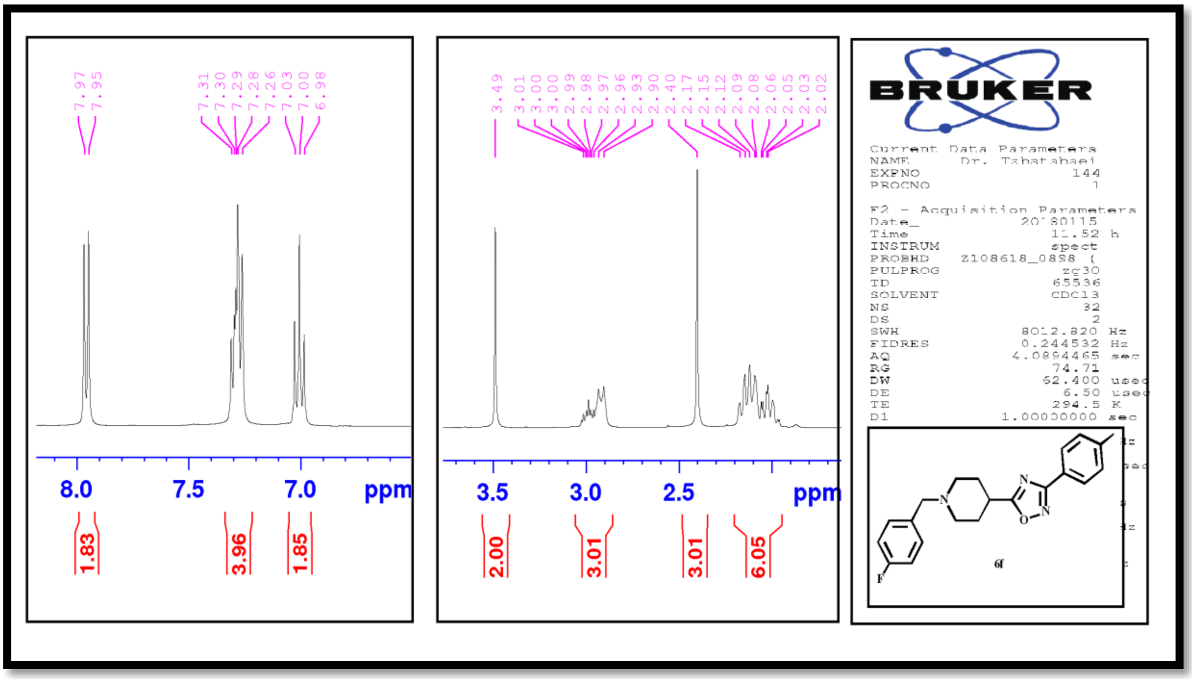
7.97
7.95
7.31
7.30
7.29
7.28
7.26
7.03
7.00
6.98
8.0
7.5
7.0
ppm
1.83
3.96
1.85
3.49
3.01
3.00
3.00
2.99
2.98
2.97
2.96
2.93
2.90
2.40
2.17
2.15
2.12
2.09
2.08
2.06
2.05
2.03
2.02
3.5
3.0
2.5
ppm
2.00
3.01
3.01
6.05
BRUKER
Current Data Parameters
NAME Dr. Tabatabaei
EXPNO 144
PROCNO 1
F2 - Acquisition Parameters
Time 11.52 h
INSTRUM spect
PULPROG zg30
TD 65536
SOLVENT CDCl3
NS 32
DS 2
SWH 8012.820 Hz
FIDRES 0.244532 Hz
AQ 4.0894465 sec
RG 74.71
DW 62.400 usec
DE 6.50 usec
TE 294.5 K
D1 1.00000000 sec
N
N
O
N
F
6f

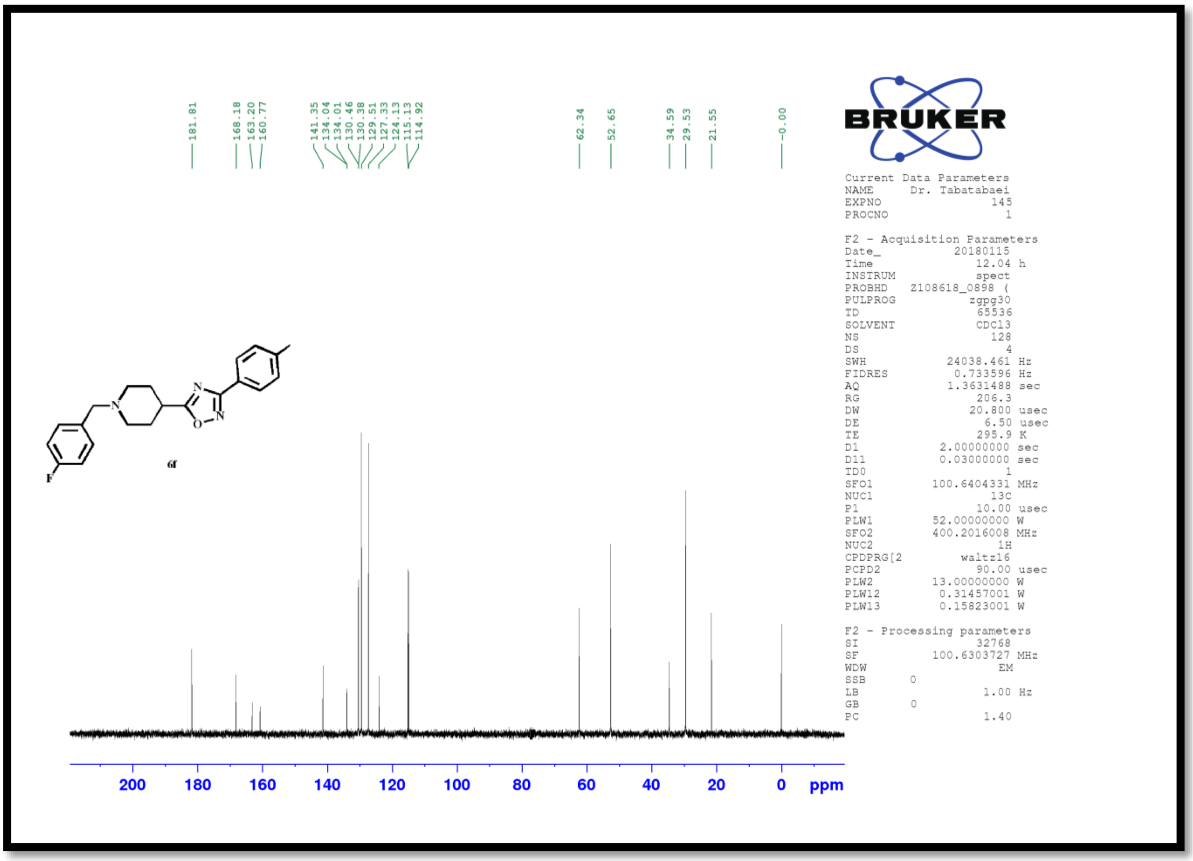
181.81
168.18
163.20
160.77
141.35
134.04
134.01
130.46
130.38
129.51
127.33
124.13
115.13
114.92
62.34
52.65
34.59
29.53
21.55
-0.00
BRUKER
Current Data Parameters
NAME Dr. Tabatabaei
EXPNO 145
PROCNO 1
F2 - Acquisition Parameters
Date_ 20180115
Time 12.04 h
INSTRUM spect
PROBHD Z108618_0898 (
PULPROG zgpg30
TD 65536
SOLVENT CDCl3
NS 128
DS 4
SWH 24038.461 Hz
FIDRES 0.733596 Hz
AQ 1.3631488 sec
RG 206.3
DW 20.800 usec
DE 6.50 usec
TE 295.9 K
D1 2.00000000 sec
D11 0.03000000 sec
TD0 1
SFO1 100.6404331 MHz
NUC1 13C
P1 10.00 usec
PLW1 52.00000000 W
SFO2 400.2016008 MHz
NUC2 1H
CPDPRG[2 waltz16
PCPD2 90.00 usec
PLW2 13.00000000 W
PLW12 0.31457001 W
PLW13 0.15823001 W
F2 - Processing parameters
SI 32768
SF 100.6303727 MHz
WDW EM
SSB 0
LB 1.00 Hz
GB 0
PC 1.40
N
N
O
N
F
6f
200
180
160
140
120
100
80
60
40
20
0
ppm

EXCLI Journal 2021;20:907-921 – ISSN 1611-2156
Received: February 27, 2021, accepted: May 10, 2021, published: May 18, 2021

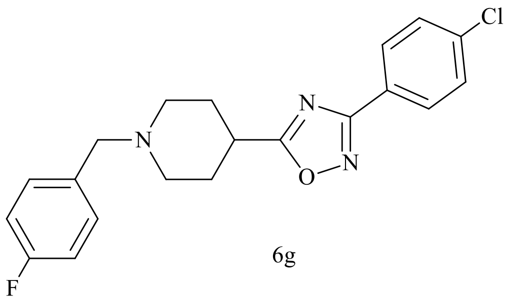

6g

3-(4-chlorophenyl)-5-(1-(4-fluorobenzyl)piperidin-4-yl)-1,2,4-oxadiazole (6g)

Light yellow powder; yield: 36.0 %; mp: 106.7-107.7 °C; IR (KBr, cm^{-1}): 1596 (C=N), 1153 (C-O); LC-MS $[M + 1]^{+}$: m/z 372; ^{1}H NMR ($CDCl_3$, 400 MHz) δ: 1.96-2.06 (m, 4H, H-piperidine), 2.10-2.19 (m, 4H, H-piperidine), 2.91-3.02 (m, 1H, H-piperidine), 3.50 (s, 2H, CH_2-benzyl), 7.01 (t, 2H, *J* = 8 Hz, H_3, H_5-benzyl), 7.26-7.31 (m, 2H, H_2, H_6-benzyl), 7.44 (d, 2H, *J* = 8 Hz, H_3, H_5-phenyl), 8.02 (d, 2H, *J* = 8 Hz, H_2, H_6-phenyl); ^{13}C NMR ($CDCl_3$, 100 MHz) δ: 29.52 ($2CH_2$), 34.59 (CH), 52.62 ($2CH_2$), 62.34 (CH_2), 115.16 (2CH), 125.48 (C), 128.73 (2CH), 130.39 (2CH), 133.96 (2CH), 137.21 (C), 160.80 (C), 163.23 (C), 167.42 (C), 182.24 (C); Anal. calcd for $C_{20}H_{19}ClFN_3O$: C, 64.60; H, 5.15; N, 11.30, found: C, 64.84; H, 5.14; N, 11.23.

EXCLI Journal 2021;20:907-921 – ISSN 1611-2156
Received: February 27, 2021, accepted: May 10, 2021, published: May 18, 2021

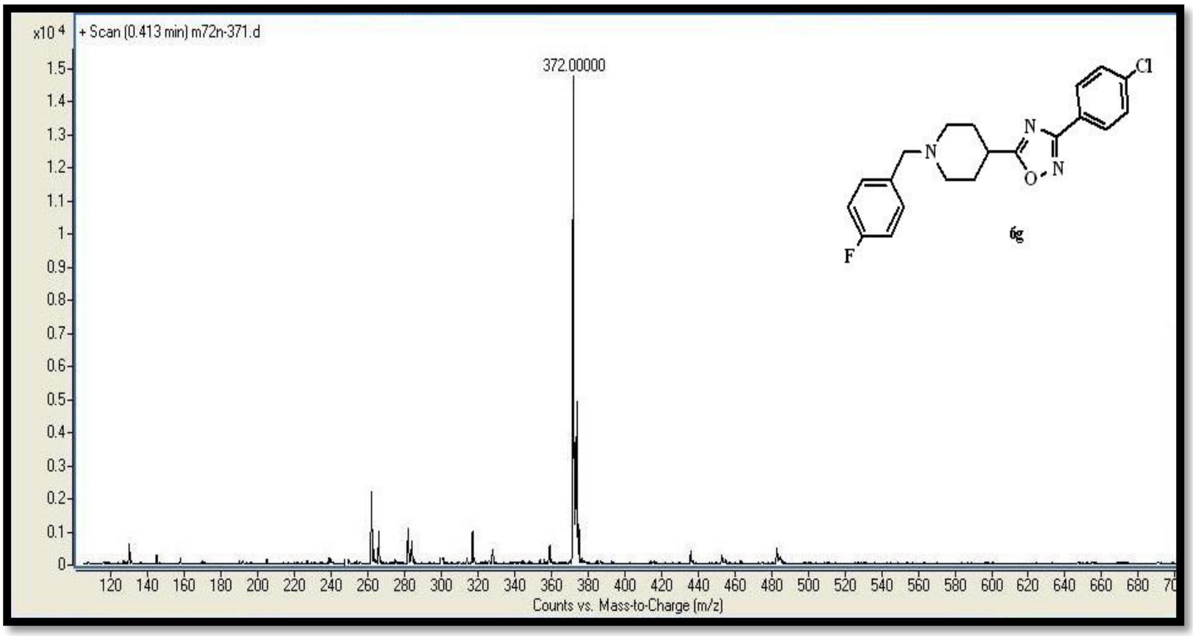

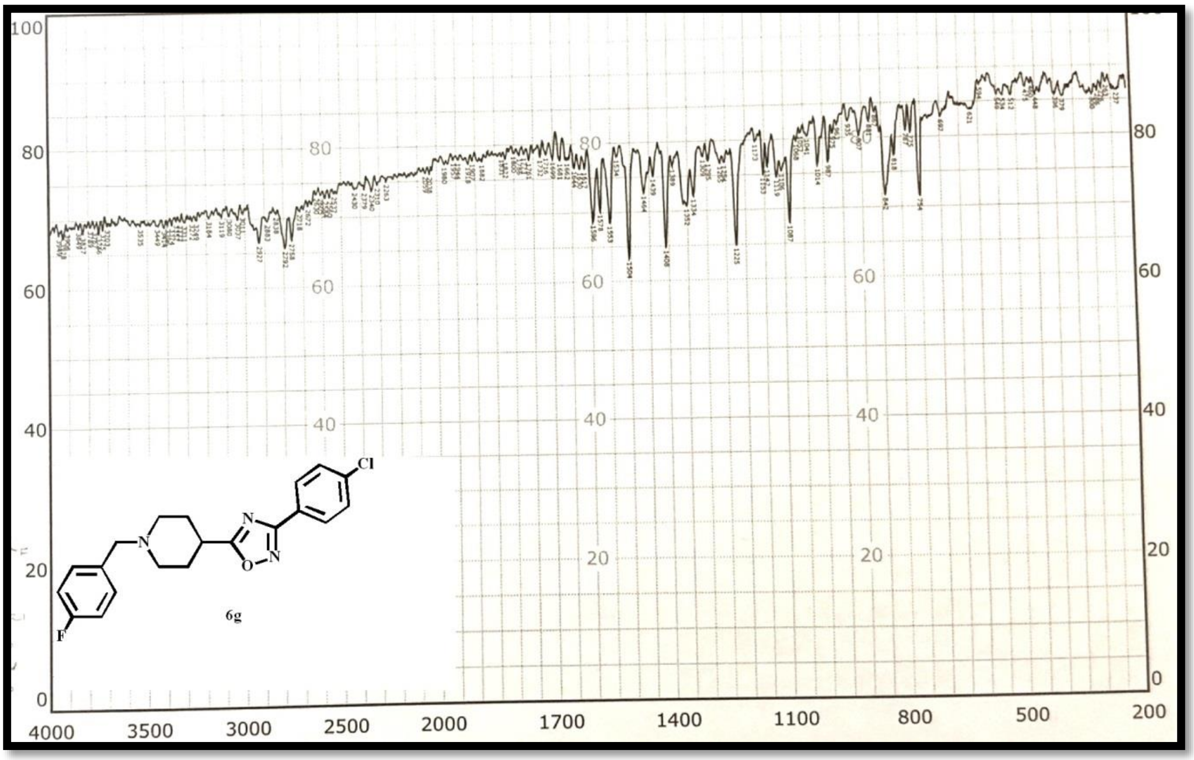

EXCLI Journal 2021;20:907-921 – ISSN 1611-2156
Received: February 27, 2021, accepted: May 10, 2021, published: May 18, 2021

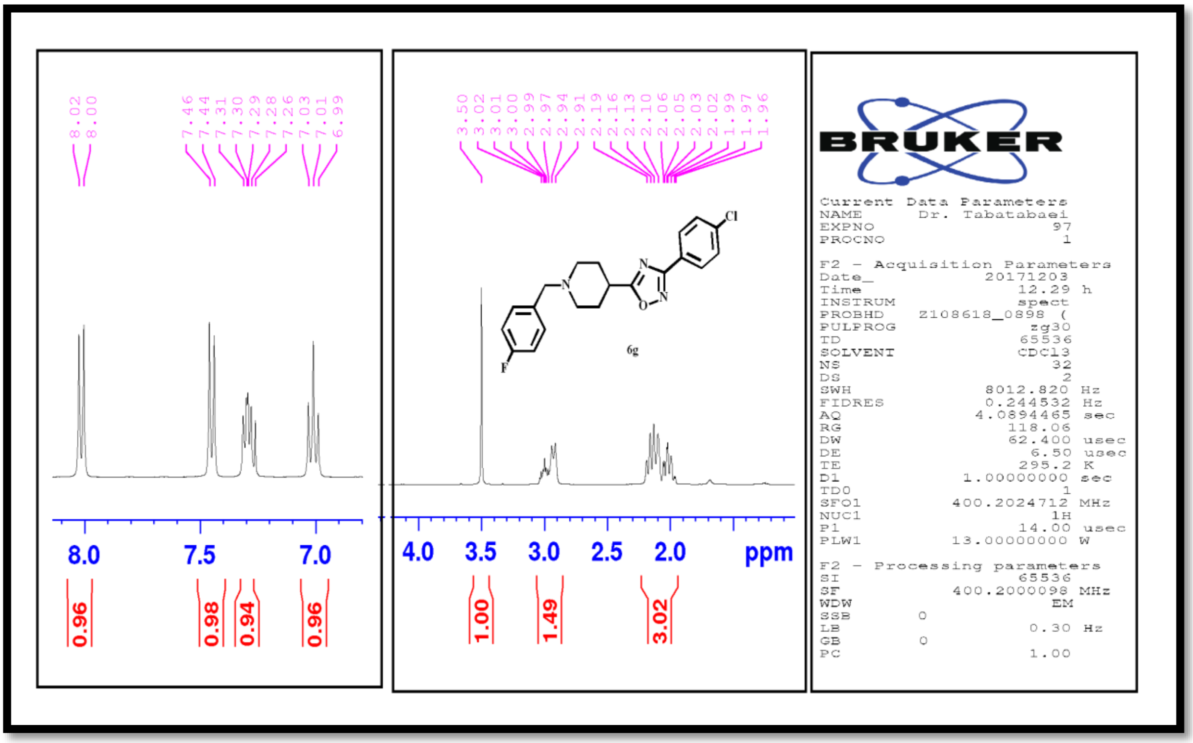

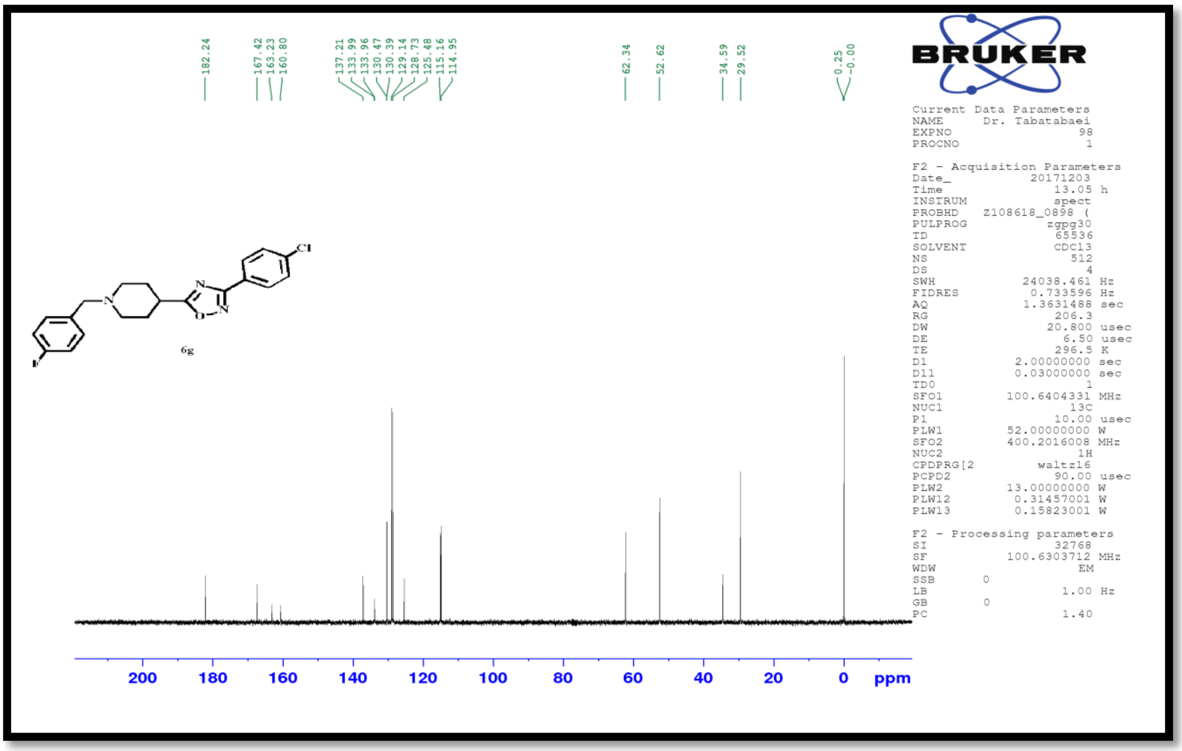

EXCLI Journal 2021;20:907-921 – ISSN 1611-2156
Received: February 27, 2021, accepted: May 10, 2021, published: May 18, 2021

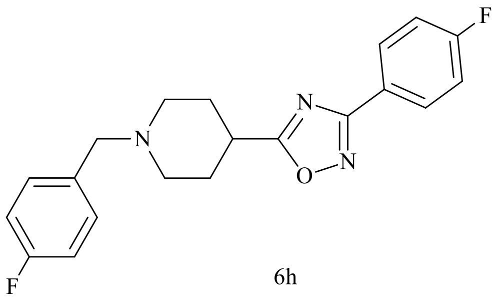

6h

5-(1-(4-fluorobenzyl)piperidin-4-yl)-3-(4-fluorophenyl)-1,2,4-oxadiazole (6h)

Light yellow powder; yield: 31.6 %; mp: 115.7-117.7 °C; IR (KBr, cm^{-1}): 1609 (C=N), 1230 (C-O); LC-MS $[M + 1]^+$: m/z 355.8; ^1H NMR ($CDCl_3$, 400 MHz) δ: 1.90-2.11 (m, 6H, H-piperidine), 2.84-2.95 (m, 3H, H-piperidine), 3.43 (s, 2H, CH_2-benzyl), 6.91-6.96 (m, 2H, H_3, H_5-benzyl), 7.08 (t, 2H, J = 8 Hz, H_3, H_5-phenyl), 7.19-7.24 (m, 2H, H_2, H_6-benzyl), 7.98-8.02 (m, 2H, H_2, H_6-phenyl); ^{13}C NMR ($CDCl_3$, 100 MHz) δ: 29.52 ($2CH_2$), 34.57 (CH), 52.63 ($2CH_2$), 62.35 (CH_2), 115.90 (2CH), 123.20 (2CH), 129.53 (C), 130.43 (2CH), 133.97 (2CH), 160.81 (C), 163.27 (C), 165.77 (C), 167.41 (C), 182.13 (C); Anal. calcd for $C_{20}H_{19}F_2N_3O$: C, 67.59; H, 5.39; N, 11.82, found: C, 67.82; H, 5.36; N, 11.76.

EXCLI Journal 2021;20:907-921 – ISSN 1611-2156
Received: February 27, 2021, accepted: May 10, 2021, published: May 18, 2021

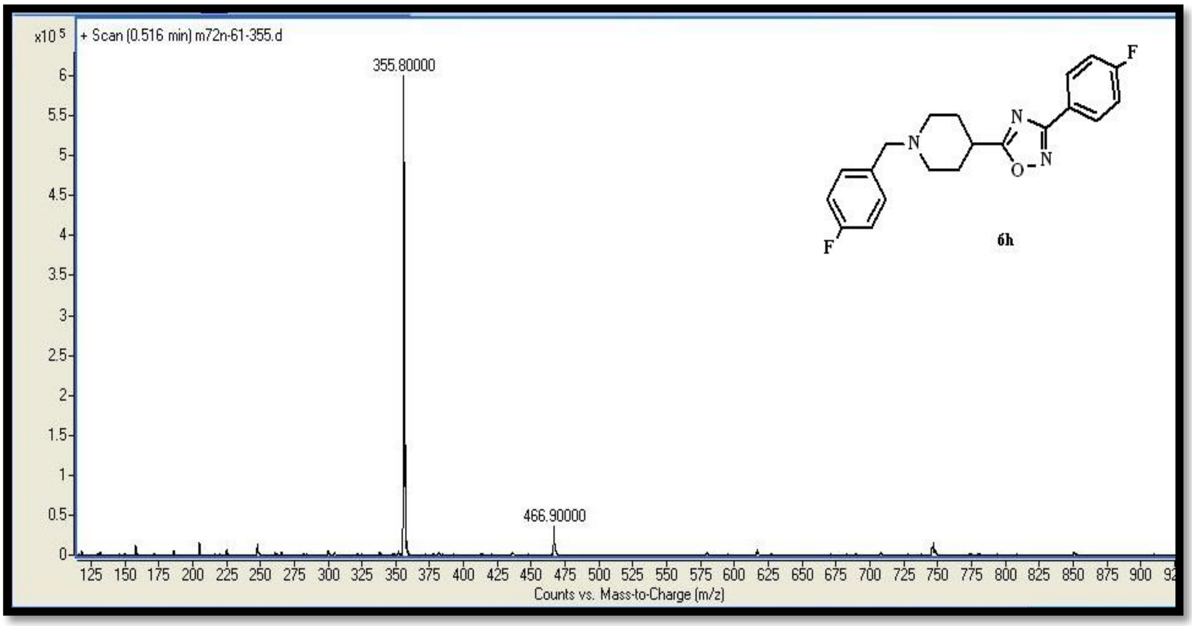

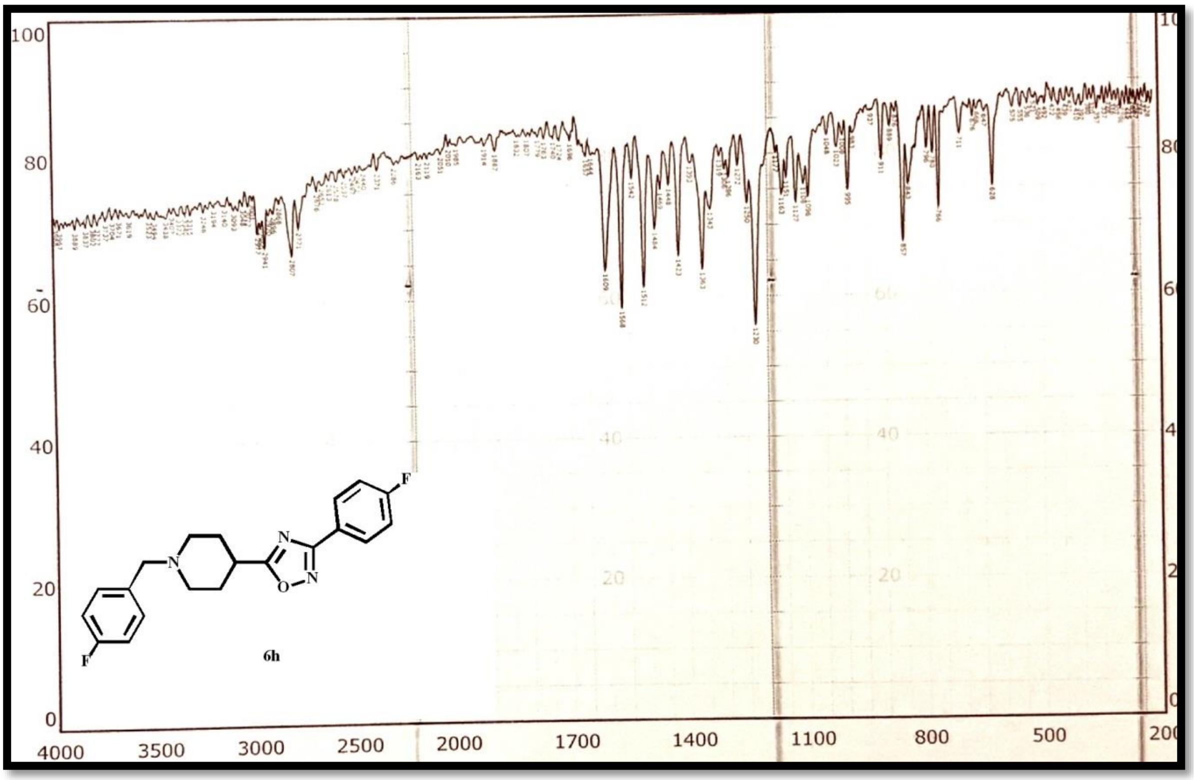

EXCLI Journal 2021;20:907-921 – ISSN 1611-2156
Received: February 27, 2021, accepted: May 10, 2021, published: May 18, 2021

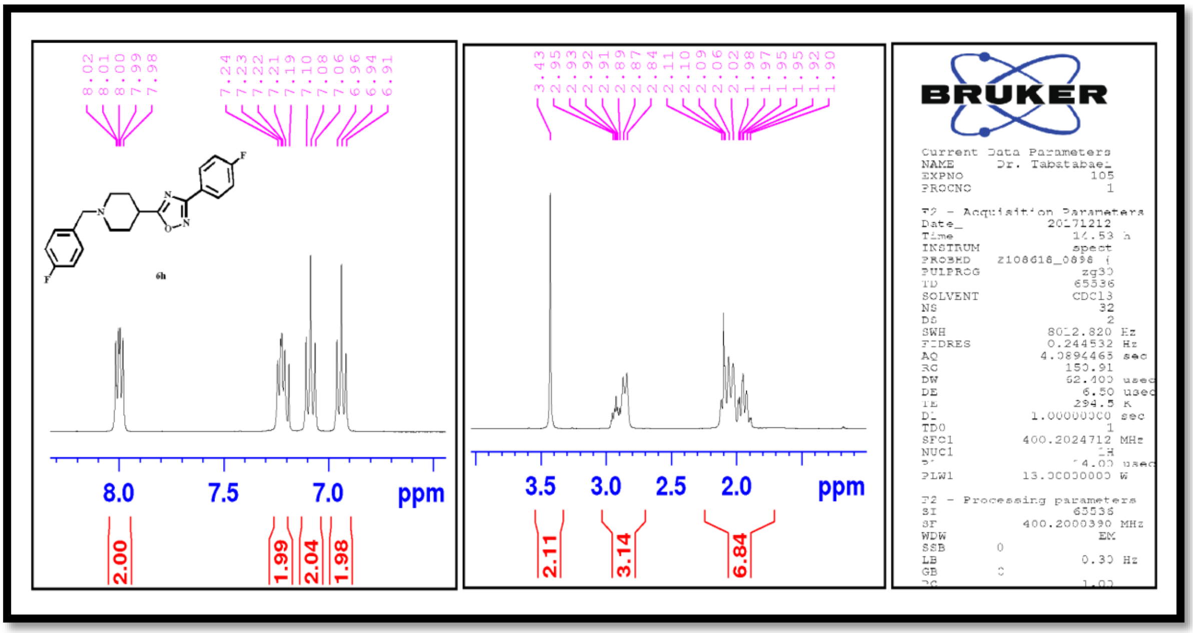

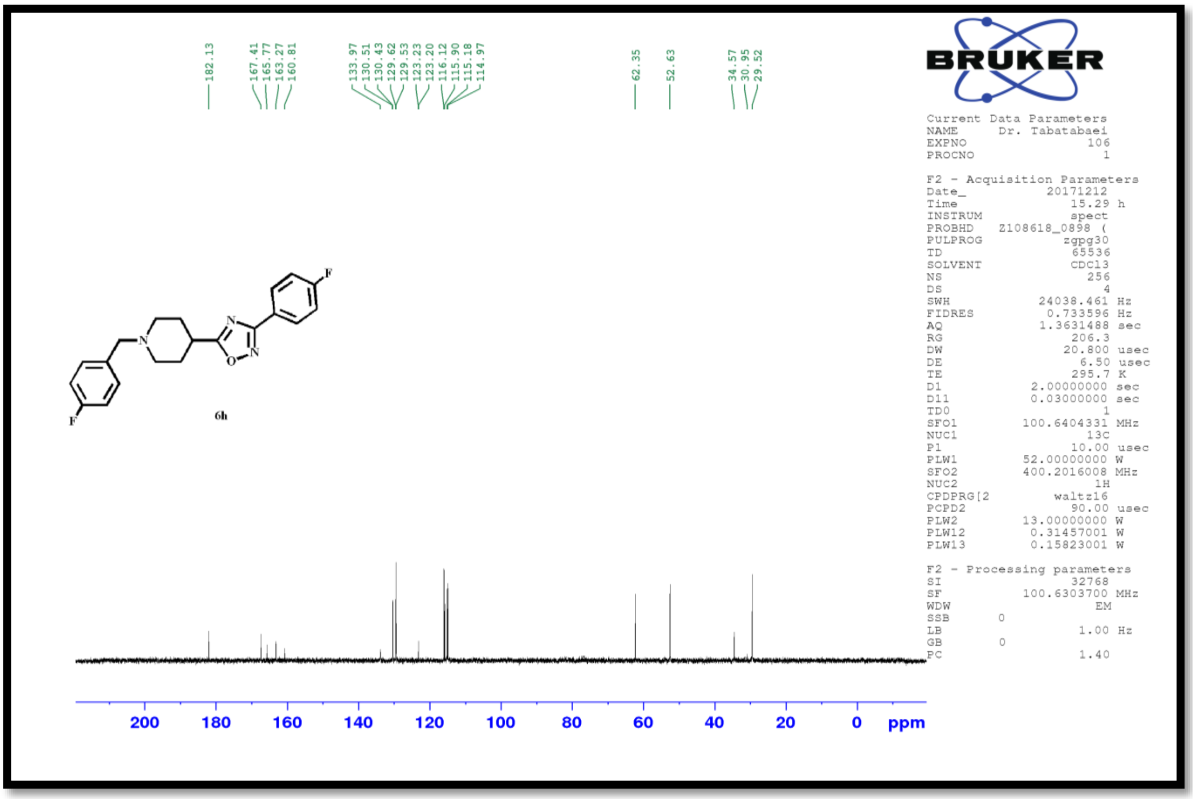

EXCLI Journal 2021;20:907-921 – ISSN 1611-2156
Received: February 27, 2021, accepted: May 10, 2021, published: May 18, 2021

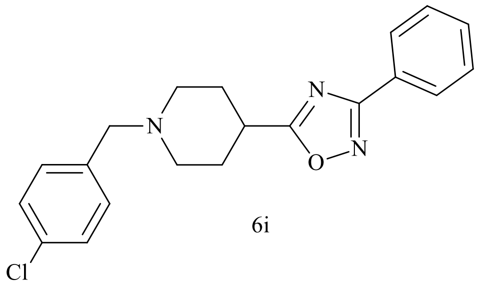

6i

5-(1-(4-chlorobenzyl)piperidin-4-yl)-3-phenyl-1,2,4-oxadiazole (6i)

Light yellow powder; yield: 39.4 %; mp: 95.6-96.7 °C; IR (KBr, cm^{-1}): 1586 (C=N), 1110 (C-O); LC-MS $[M + 1]^+$: m/z 353.8; ^1H NMR ($CDCl_3$, 400 MHz) δ: 1.96-2.18 (m, 6H, H-piperidine), 2.89-3.02 (m, 3H, H-piperidine), 3.48 (s, 2H, CH_2-benzyl), 7.25-7.30 (m, 4H, H_2, H_3, H_5, H_6-benzyl), 7.46-7.48 (m, 3H, H_3, H_4, H_5-phenyl), 8.06-8.08 (m, 2H, H_2, H_6-phenyl); ^{13}C NMR ($CDCl_3$, 100 MHz) δ: 29.50 ($2CH_2$), 34.51 (CH), 52.65 ($2CH_2$), 62.33 (CH_2), 126.93 (C), 127.33 (2CH), 127.42 (2CH), 128.37 (2CH), 130.22 (2CH), 131.05 (CH), 132.70 (C), 136.89 (C), 168.16 (C), 181.94 (C); Anal. calcd for $C_{20}H_{20}ClN_3O$: C, 67.89; H, 5.70; N, 11.88, found: C, 68.12; H, 5.68; N, 11.93.

EXCLI Journal 2021;20:907-921 – ISSN 1611-2156
Received: February 27, 2021, accepted: May 10, 2021, published: May 18, 2021

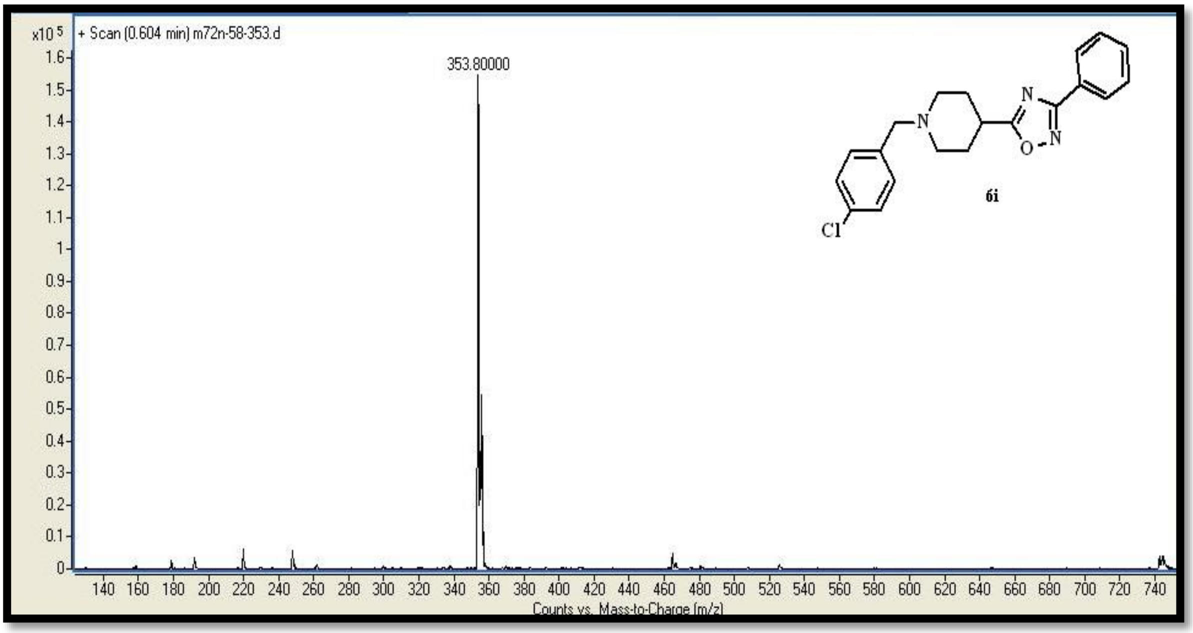

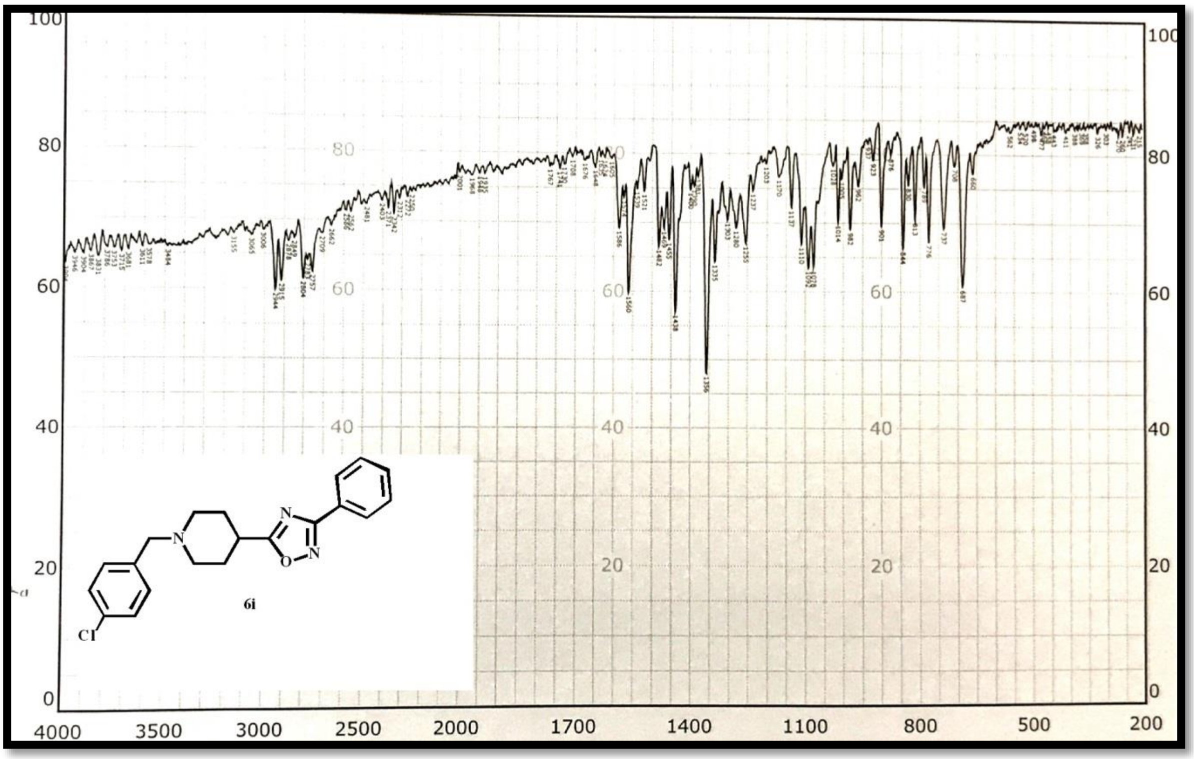

EXCLI Journal 2021;20:907-921 – ISSN 1611-2156
Received: February 27, 2021, accepted: May 10, 2021, published: May 18, 2021

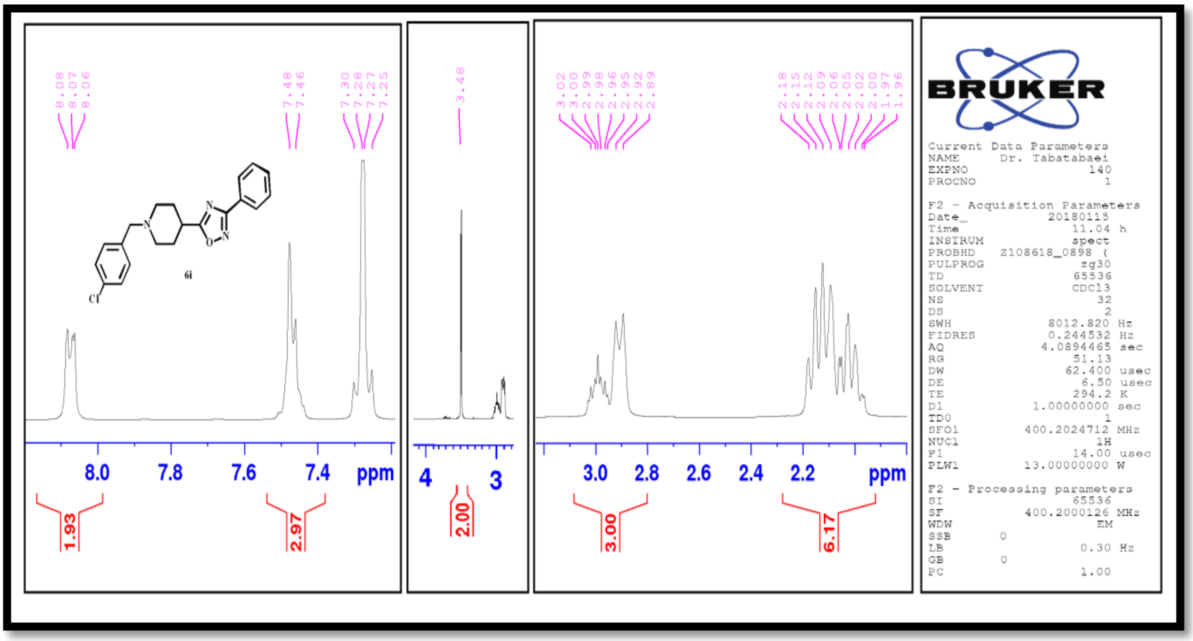

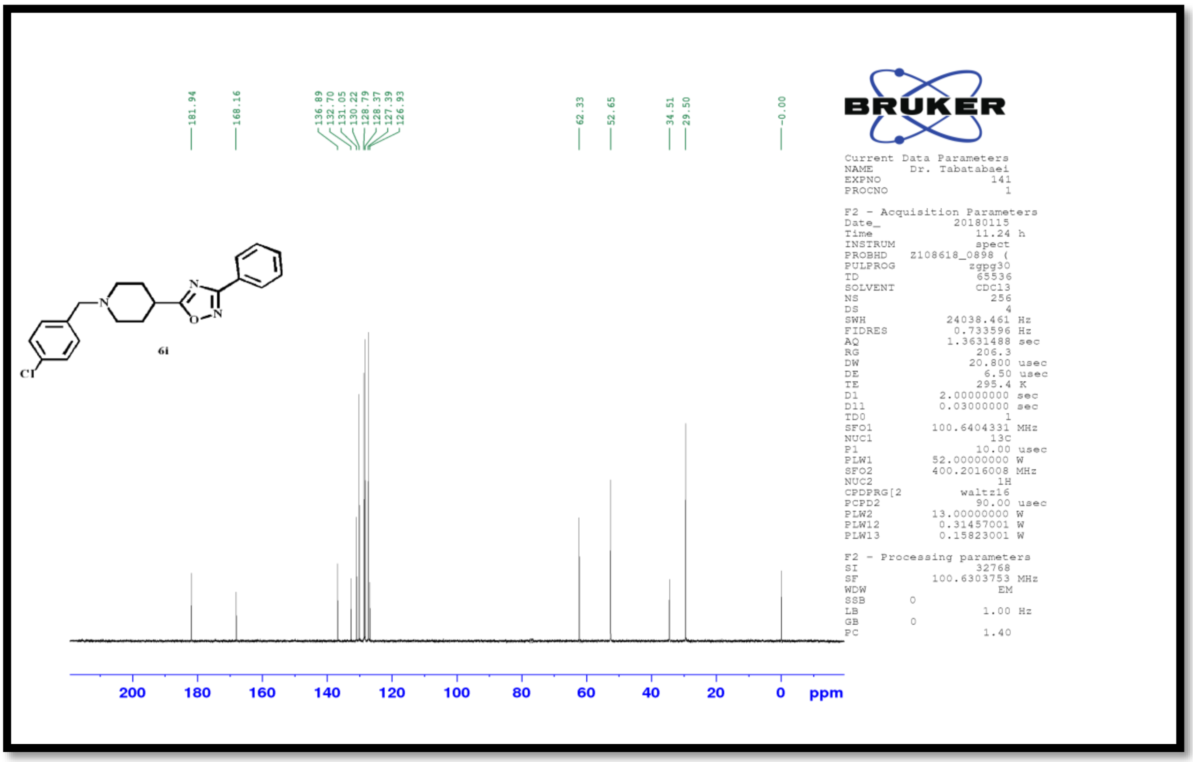

EXCLI Journal 2021;20:907-921 – ISSN 1611-2156
Received: February 27, 2021, accepted: May 10, 2021, published: May 18, 2021

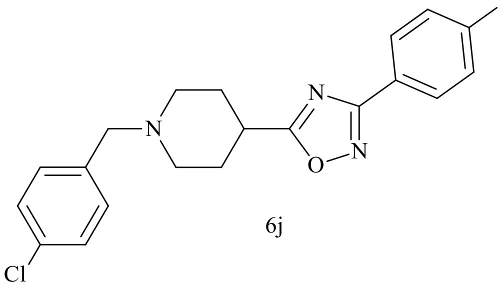

6j

5-(1-(4-chlorobenzyl)piperidin-4-yl)-3-(p-tolyl)-1,2,4-oxadiazole (6j)

Light yellow powder; yield: 40.3 %; mp: 103.5-104.7 °C; IR (KBr, cm^{-1}): 1582 (C=N), 1213 (C-O), 1358,1486 (CH_3); LC-MS $[M + 1]^+$: m/z 367.8; ^1H NMR ($CDCl_3$, 400 MHz) δ: 1.89-2.11 (m, 6H, H-piperidine), 2.33 (s, 3H, CH_3), 2.82-2.94 (m, 3H, H-piperidine), 3.42 (s, 2H, CH_2-benzyl), 7.18-7.23 (m, 6H, H_2, H_3, H_5, H_6-benzyl, H_3, H_5-phenyl), 7.87 (d, 2H, J = 8 Hz, H_2, H_6-phenyl); ^{13}C NMR ($CDCl_3$, 100 MHz) δ: 21.59 (CH_3), 29.52 ($2CH_2$), 34.55 (CH), 52.70 ($2CH_2$), 62.37 (CH_2), 124.13 (C), 127.35 (2CH), 128.43 (2CH), 129.54 (2CH), 130.28 (2CH), 132.78 (C), 136.89 (C), 141.39 (C), 168.22 (C), 181.78 (C); Anal. calcd for $C_{21}H_{22}ClN_3O$: C, 68.56; H, 6.03; N, 11.42, found: C, 68.79; H, 6.01; N, 11.37.

EXCLI Journal 2021;20:907-921 – ISSN 1611-2156
Received: February 27, 2021, accepted: May 10, 2021, published: May 18, 2021

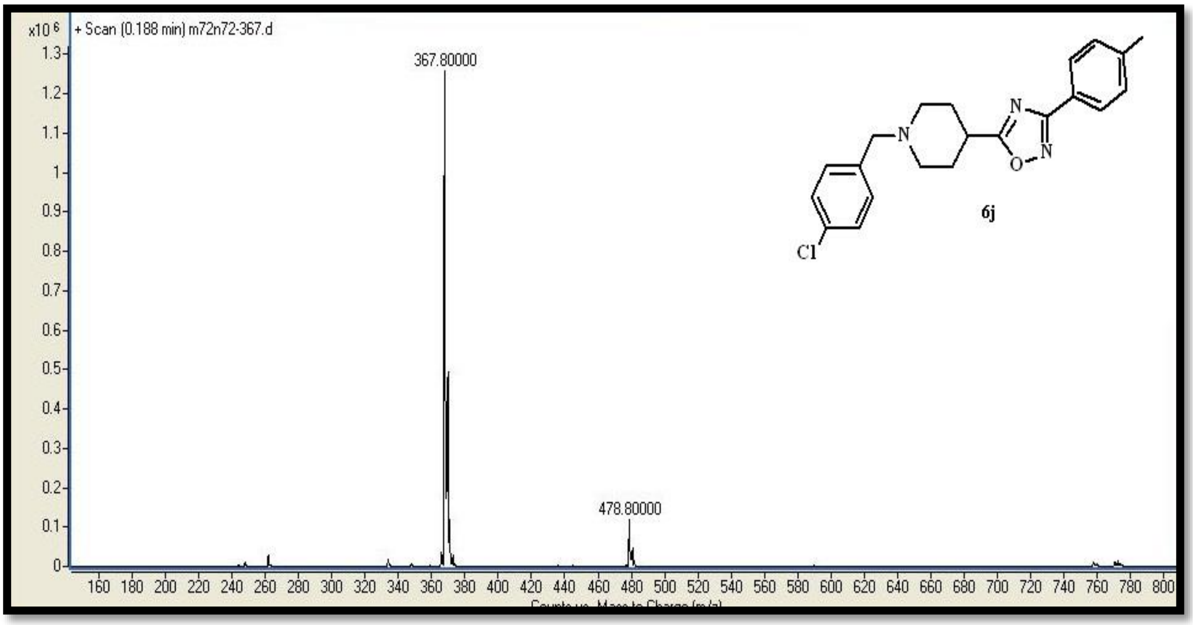

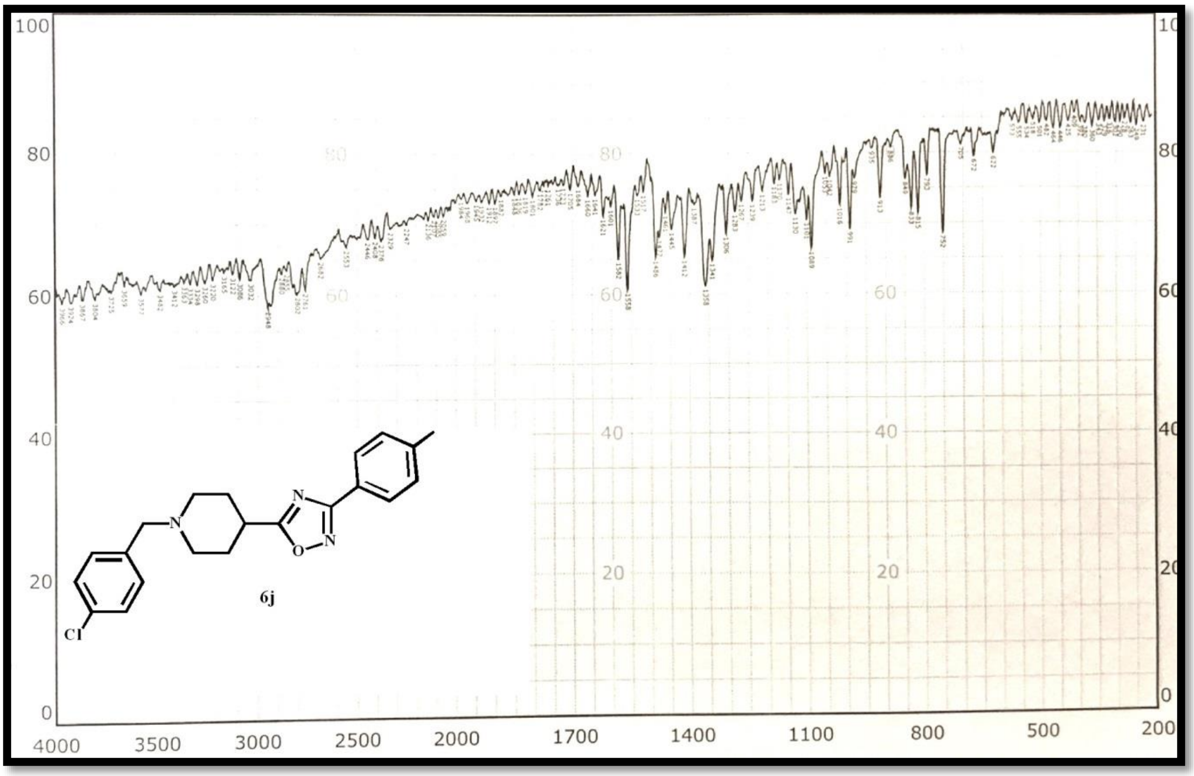

EXCLI Journal 2021;20:907-921 – ISSN 1611-2156
Received: February 27, 2021, accepted: May 10, 2021, published: May 18, 2021

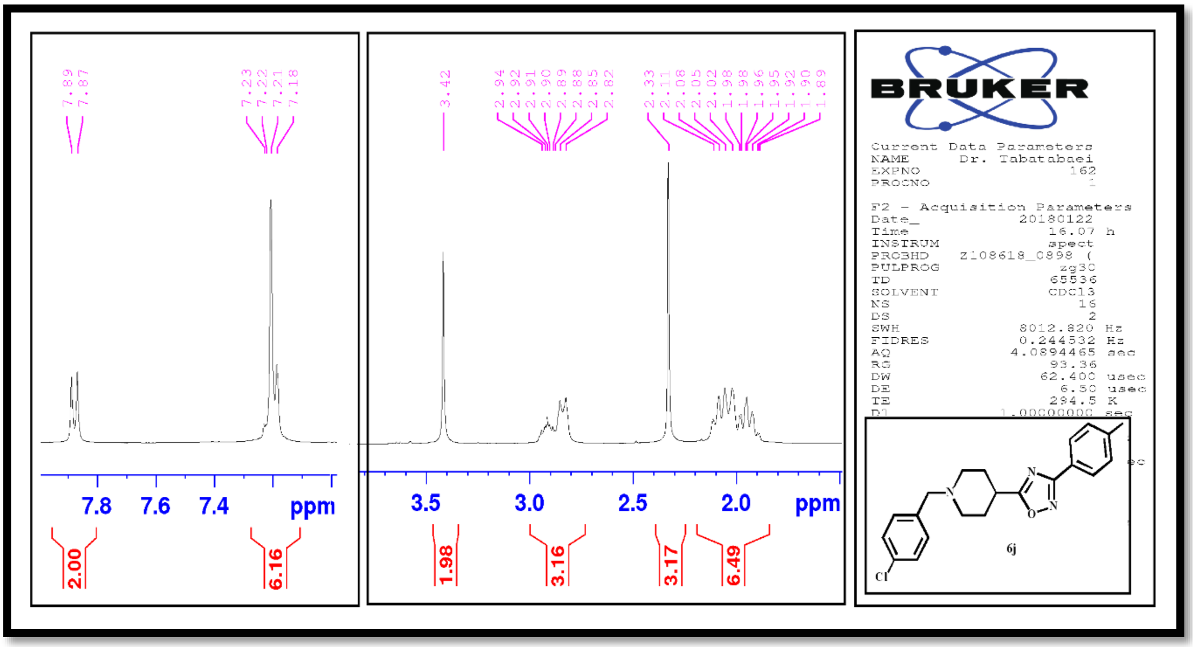

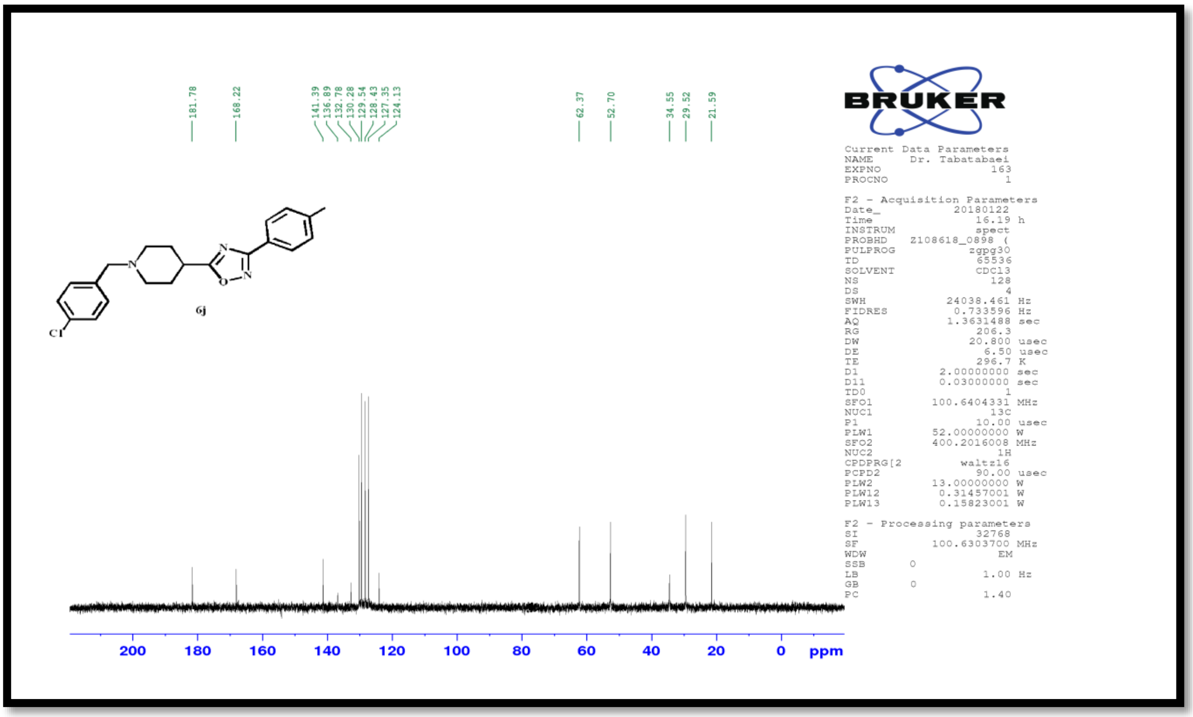

EXCLI Journal 2021;20:907-921 – ISSN 1611-2156
Received: February 27, 2021, accepted: May 10, 2021, published: May 18, 2021

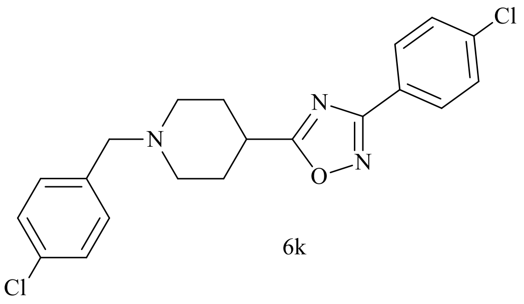

6k

5-(1-(4-chlorobenzyl)piperidin-4-yl)-3-(4-chlorophenyl)-1,2,4-oxadiazole (6k)

Light yellow powder; yield: 51.7 %; mp: 110.7-113.7 °C; IR (KBr, cm^{-1}): 1582 (C=N), 1213 (C-O); LC-MS $[M + 1]^+$: m/z 387.8; ^1H NMR ($CDCl_3$, 400 MHz) δ: 1.88-2.11 (m, 6H, H-piperidine), 2.82-2.95 (m, 3H, H-piperidine), 3.41 (s, 2H, CH_2-benzyl), 7.18-7.22 (m, 4H, H_2, H_3, H_5, H_6-benzyl), 7.35 (d, 2H, J = 8 Hz, H_3, H_5-phenyl), 7.92 (d, 2H, J = 8 Hz, H_2, H_6-phenyl); ^{13}C NMR ($CDCl_3$, 100 MHz) δ: 29.47 ($2CH_2$), 34.50 (CH), 52.63 ($2CH_2$), 62.36 (CH_2), 125.45 (C), 128.75 (2CH), 129.16 (2CH), 129.46 (2CH), 130.32 (2CH), 132.82 (C), 136.73 (C), 137.23 (C), 167.41 (C), 182.19 (C); Anal. calcd for $C_{20}H_{19}Cl_2N_3O$: C, 61.86; H, 4.93; N, 10.82, found: C, 62.09; H, 4.91; N, 10.78.

EXCLI Journal 2021;20:907-921 – ISSN 1611-2156
Received: February 27, 2021, accepted: May 10, 2021, published: May 18, 2021

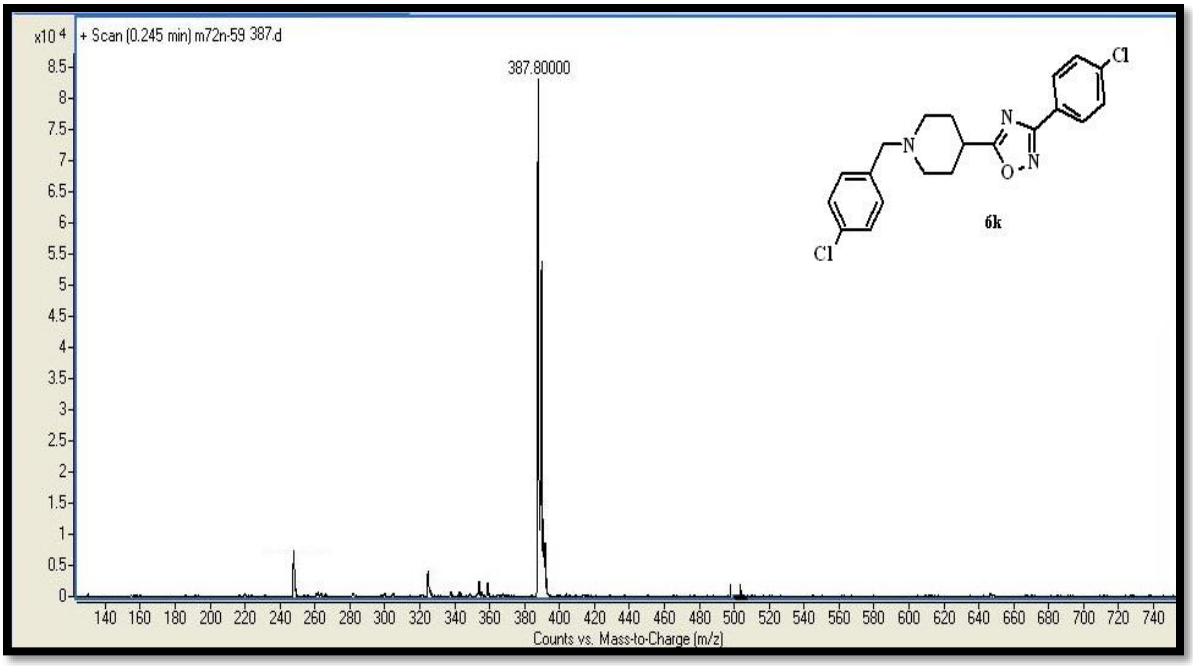

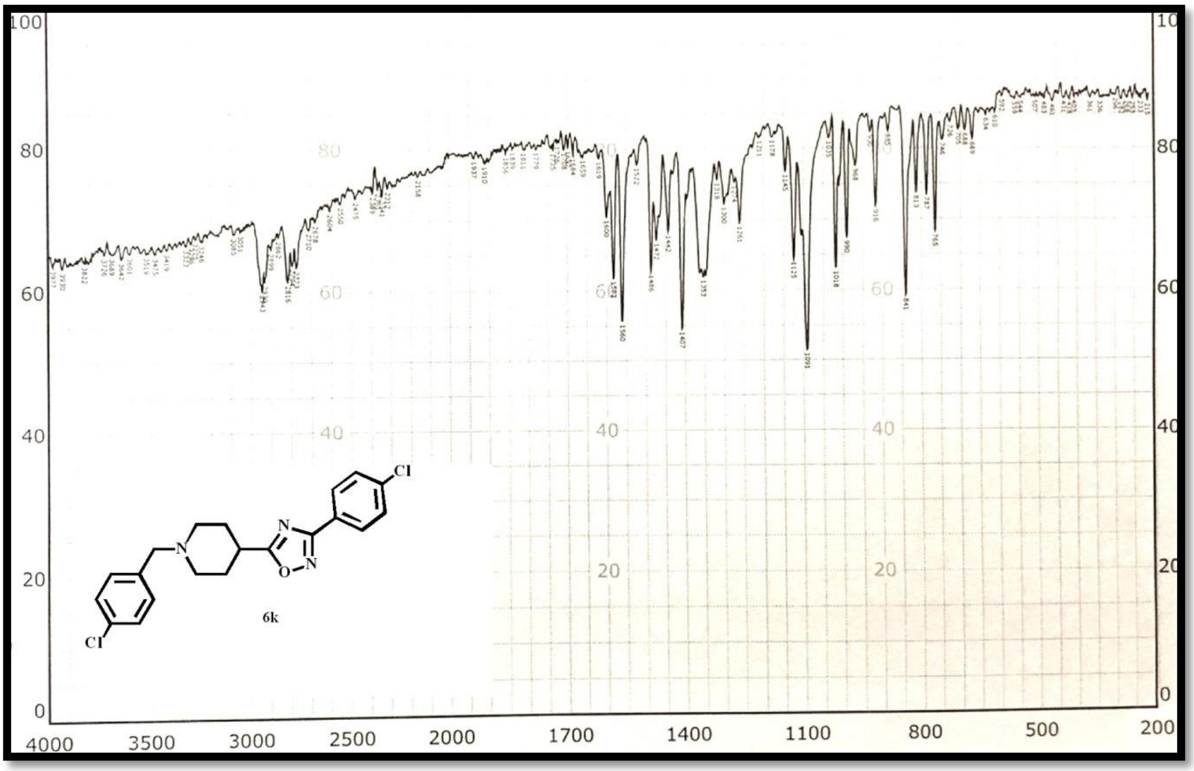

EXCLI Journal 2021;20:907-921 – ISSN 1611-2156
Received: February 27, 2021, accepted: May 10, 2021, published: May 18, 2021

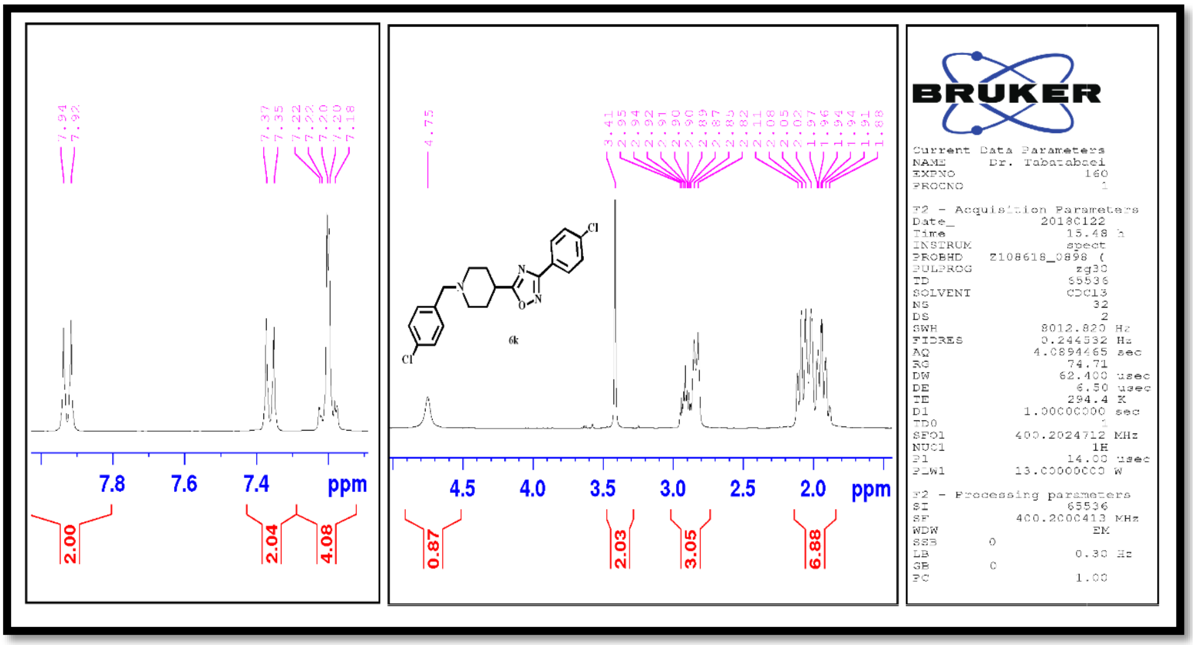

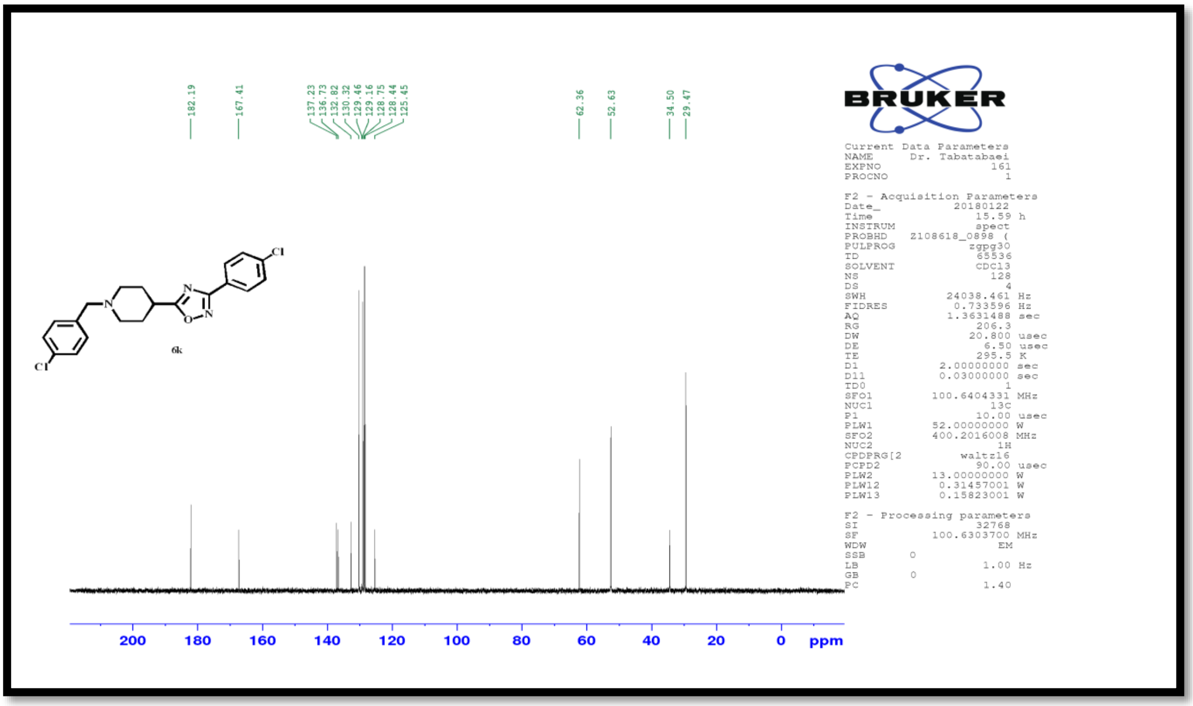

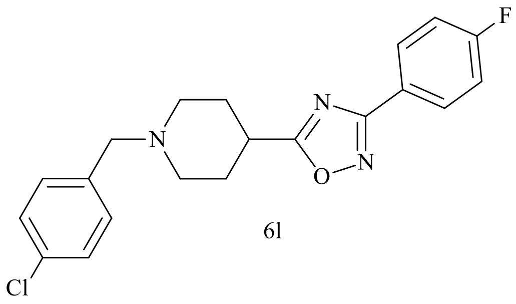

6l

5-(1-(4-chlorobenzyl)piperidin-4-yl)-3-(4-fluorophenyl)-1,2,4-oxadiazole (6l)

Light yellow powder; yield: 40.7 %; mp: 89.9-90.4 °C; IR (KBr, cm^{-1}): 1600 (C=N), 1221 (C-O); LC-MS $[M + 1]^+$: m/z 371.8; ^{1}H NMR ($CDCl_3$, 400 MHz) δ: 1.99-2.19 (m, 6H, H-piperidine), 2.90-3.02 (m, 3H, H-piperidine), 3.49 (s, 2H, CH_2-benzyl), 7.13-7.18 (m, 2H, H_3, H_5-phenyl), 7.26-7.29 (m, 4H, H_2, H_3, H_5, H_6-benzyl), 8.05-8.09 (m, 2H, H_2, H_6-phenyl); ^{13}C NMR ($CDCl_3$, 100 MHz) δ: 29.51 ($2CH_2$), 34.51 (CH), 52.66 ($2CH_2$), 62.35 (CH_2), 115.88 (2CH), 123.16 (C), 129.51 (2CH), 130.23 (2CH), 132.74 (2CH), 136.86 (C), 163.24 (C), 165.73 (C), 167.37 (C), 182.09 (C); Anal. calcd for $C_{20}H_{19}ClFN_3O$: C, 64.60; H, 5.15; N, 11.30, found: C, 64.81; H, 5.12; N, 11.37.

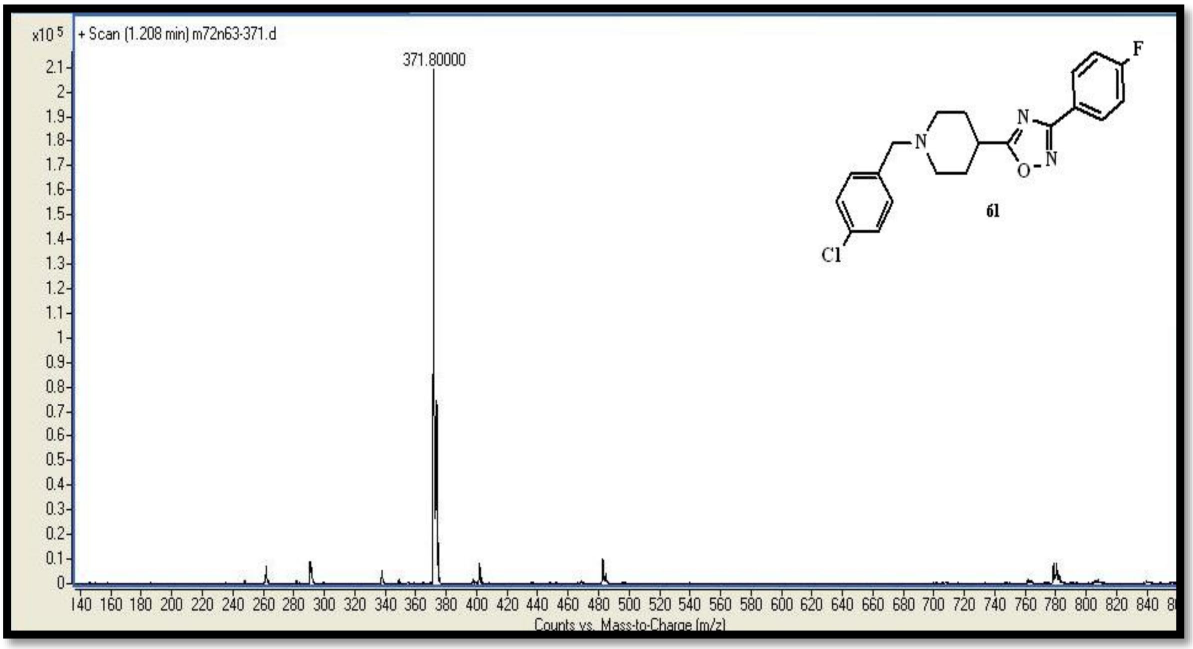
x10 5
+ Scan (1.208 min) m72n63-371.d
371.80000
6l
Counts vs. Mass-to-Charge (m/z)

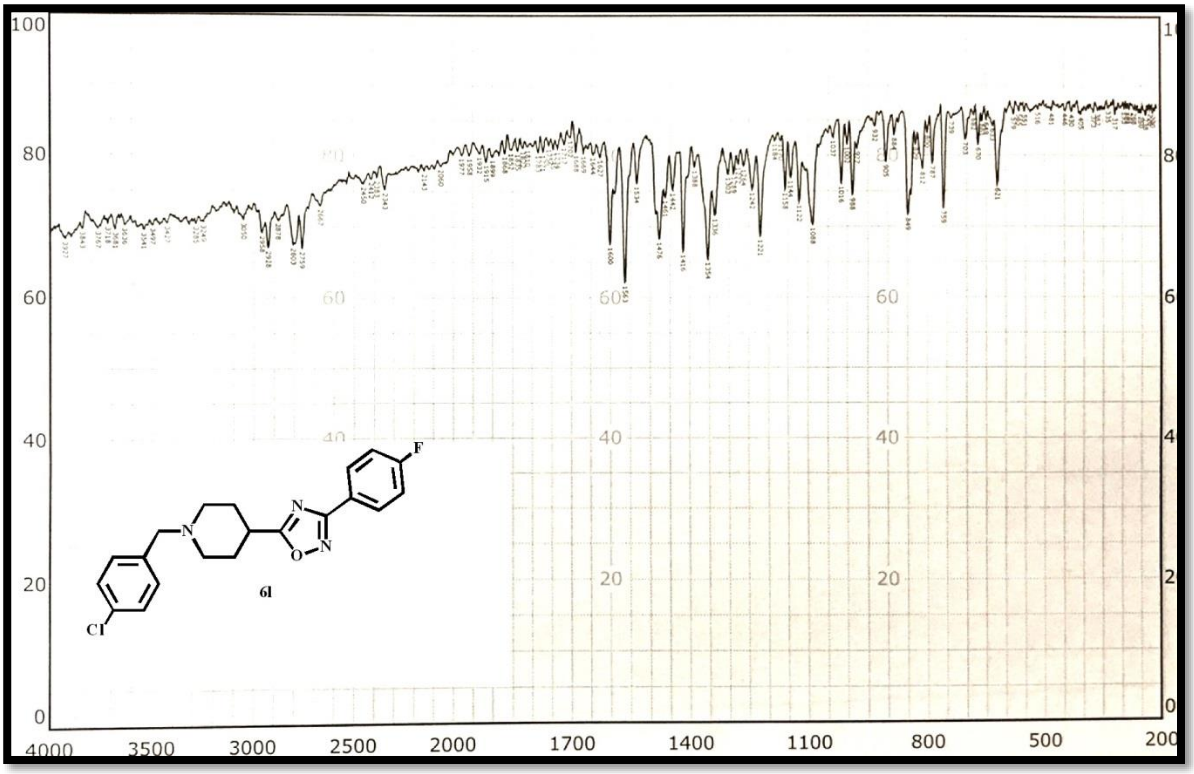
6l

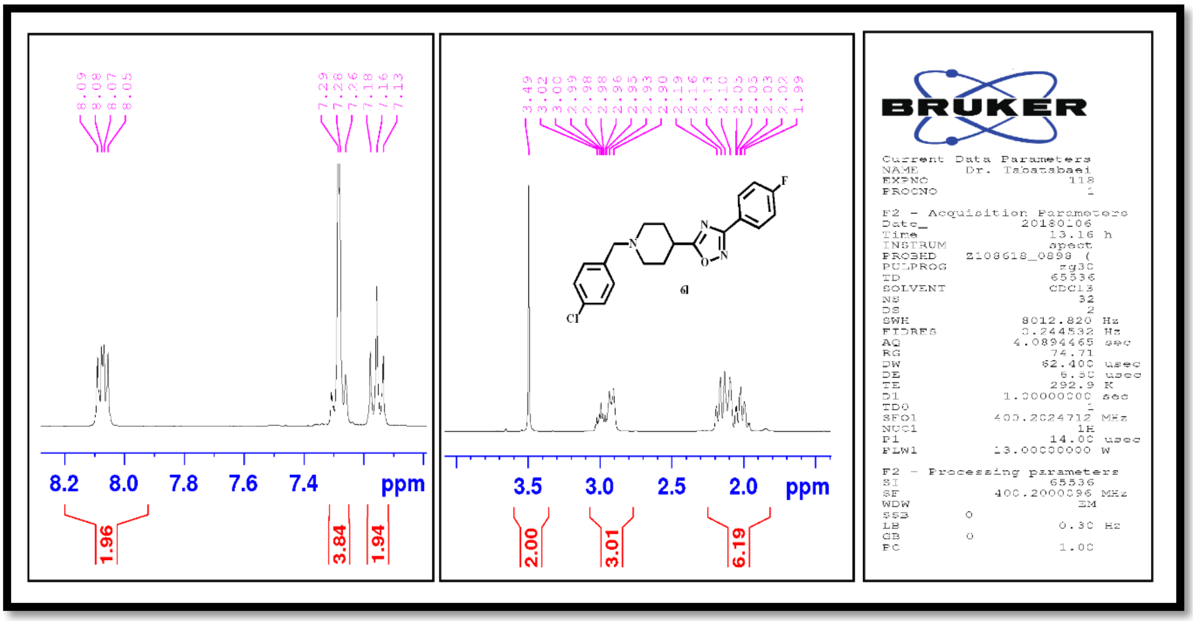
8.09
8.08
8.07
8.05
7.29
7.28
7.26
7.26
7.18
7.16
7.13
8.2 8.0 7.8 7.6 7.4 ppm
1.96
3.84
1.94
3.49
3.02
3.00
2.99
2.98
2.98
2.96
2.95
2.93
2.90
2.19
2.16
2.13
2.10
2.05
2.05
2.03
2.02
1.99
3.5 3.0 2.5 2.0 ppm
2.00
3.01
6.19
6l
Cl
F
BRUKER
Current Data Parameters
NAME Dr. Tabatabaei
EXPNO 118
PROCNO 1
F2 - Acquisition Parameters
Date_ 20180106
Time 13.16 h
INSTRUM spect
PROBHD Z108618_0898 (
PULPROG zg30
TD 65536
SOLVENT CDCl3
NS 32
DS 2
SWH 8012.820 Hz
FIDRES 0.244532 Hz
AQ 4.0894465 sec
RG 74.71
DW 62.400 usec
DE 6.50 usec
TE 292.9 K
D1 1.00000000 sec
TD0 1
SFO1 400.2024712 MHz
NUC1 1H
P1 14.00 usec
PLW1 13.00000000 W
F2 - Processing parameters
SI 65536
SF 400.2000096 MHz
WDW EM
SSB 0
LB 0.30 Hz
GB 0
PC 1.00

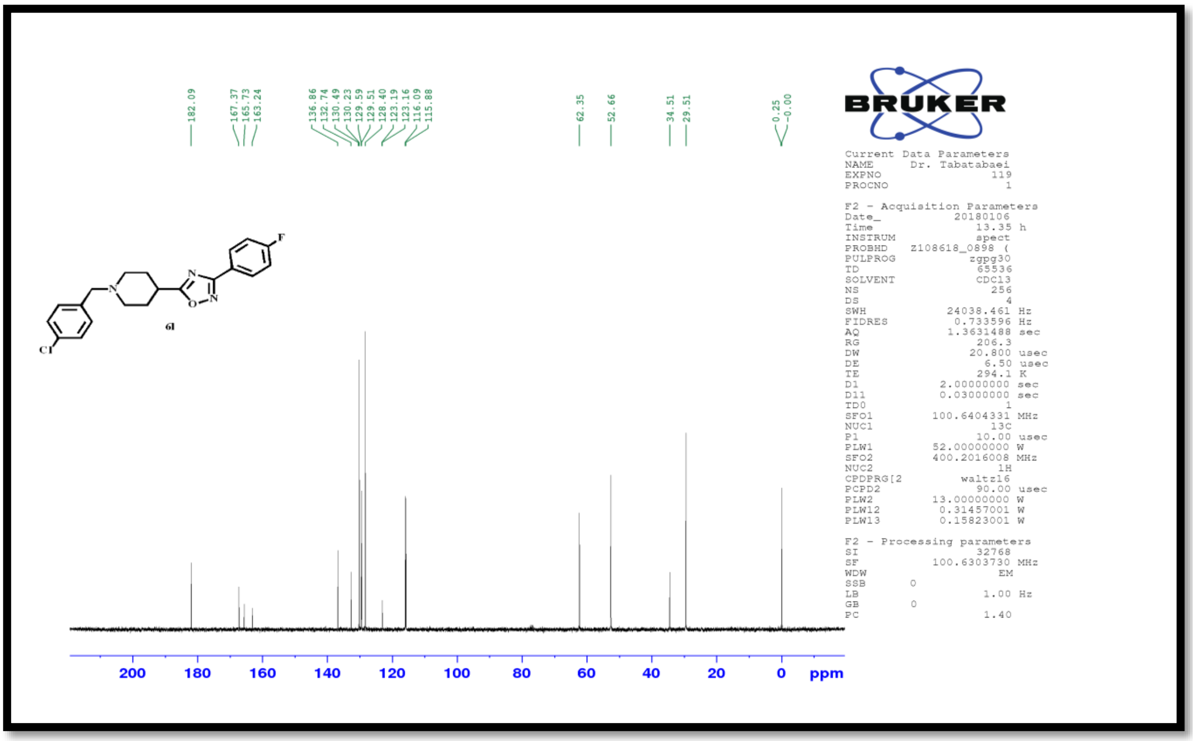
182.09
167.37
165.73
163.24
136.86
132.74
130.49
130.23
129.59
129.51
128.40
123.19
123.16
116.09
115.88
62.35
52.66
34.51
29.51
0.25
-0.00
6l
Cl
F
200 180 160 140 120 100 80 60 40 20 0 ppm
BRUKER
Current Data Parameters
NAME Dr. Tabatabaei
EXPNO 119
PROCNO 1
F2 - Acquisition Parameters
Date_ 20180106
Time 13.35 h
INSTRUM spect
PROBHD Z108618_0898 (
PULPROG zgpg30
TD 65536
SOLVENT CDCl3
NS 256
DS 4
SWH 24038.461 Hz
FIDRES 0.733596 Hz
AQ 1.3631488 sec
RG 206.3
DW 20.800 usec
DE 6.50 usec
TE 294.1 K
D1 2.00000000 sec
D11 0.03000000 sec
TD0 1
SFO1 100.6404331 MHz
NUC1 13C
P1 10.00 usec
PLW1 52.00000000 W
SFO2 400.2016008 MHz
NUC2 1H
CPDPRG[2 waltz16
PCPD2 90.00 usec
PLW2 13.00000000 W
PLW12 0.31457001 W
PLW13 0.15823001 W
F2 - Processing parameters
SI 32768
SF 100.6303730 MHz
WDW EM
SSB 0
LB 1.00 Hz
GB 0
PC 1.40

EXCLI Journal 2021;20:907-921 – ISSN 1611-2156
Received: February 27, 2021, accepted: May 10, 2021, published: May 18, 2021

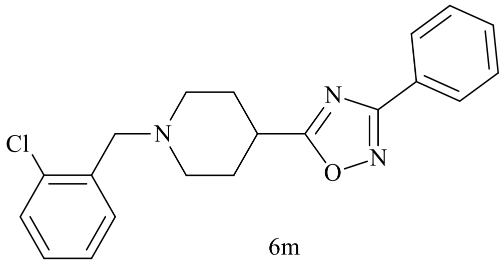

6m

5-(1-(2-chlorobenzyl)piperidin-4-yl)-3-phenyl-1,2,4-oxadiazole (6m)

Light yellow powder; yield: 28.6 %; mp: 61.5-62.2 °C; IR (KBr, cm^{-1}): 1583 (C=N), 1134 (C-O); LC-MS $[M + 1]^+$: m/z 354; ^1H NMR ($CDCl_3$, 400 MHz) δ: 2.01-2.18 (m, 6H, H-piperidine), 2.93-3.02 (m, 3H, H-piperidine), 3.50 (s, 2H, CH_2-benzyl), 7.13-7.15 (m, 3H, H_4, H_5, H_6-benzyl), 7.21-7.25 (m, 3H, H_3, H_4, H_5-phenyl), 7.44-7.49 (m, 1H, H_3-benzyl), 8.06 (d, 2H, J = 8 Hz, H_2, H_6-phenyl); ^{13}C NMR ($CDCl_3$, 100 MHz) δ: 29.56 ($2CH_2$), 34.67 (CH), 52.69 ($2CH_2$), 62.92 (CH_2), 126.89 (C), 127.00 (CH), 127.42 (2CH), 128.79 (2CH), 128.93 (2CH), 129.04 (CH), 131.04 (CH), 135.14 (C), 136.66 (C), 168.18 (C), 182.09 (C); Anal. calcd for $C_{20}H_{20}ClN_3O$: C, 67.89; H, 5.70; N, 11.88, found: C, 68.13; H, 5.67; N, 11.80.

EXCLI Journal 2021;20:907-921 – ISSN 1611-2156
Received: February 27, 2021, accepted: May 10, 2021, published: May 18, 2021

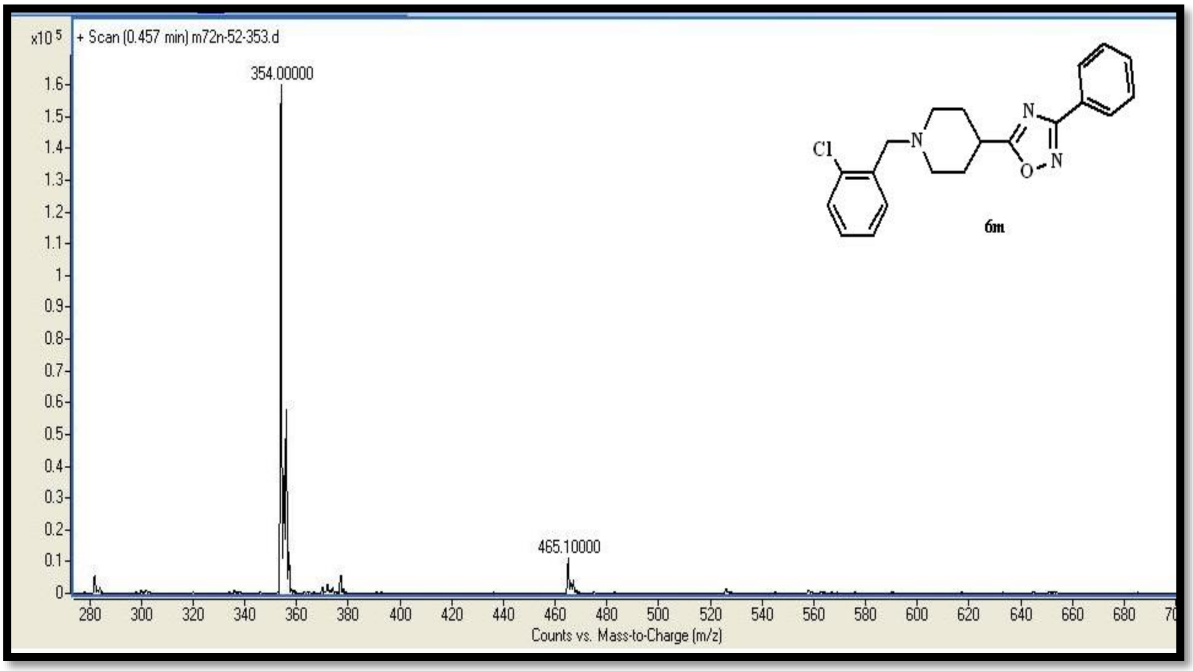

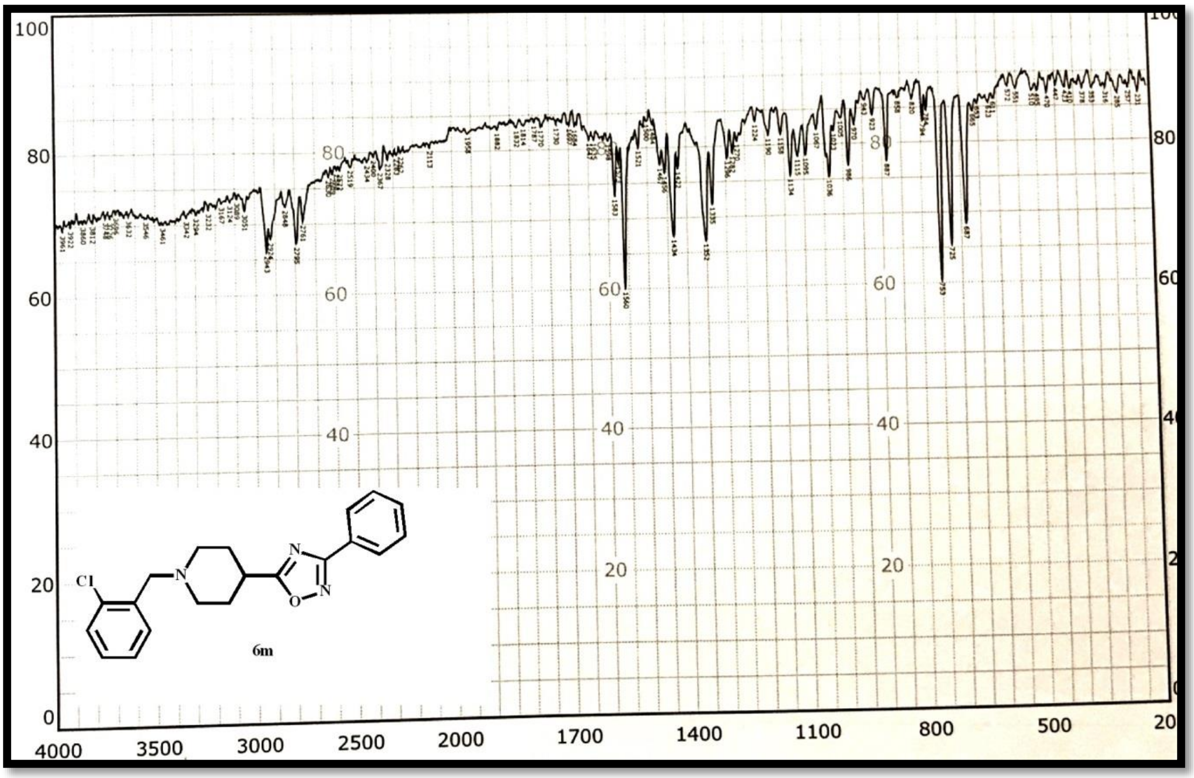

EXCLI Journal 2021;20:907-921 – ISSN 1611-2156
Received: February 27, 2021, accepted: May 10, 2021, published: May 18, 2021

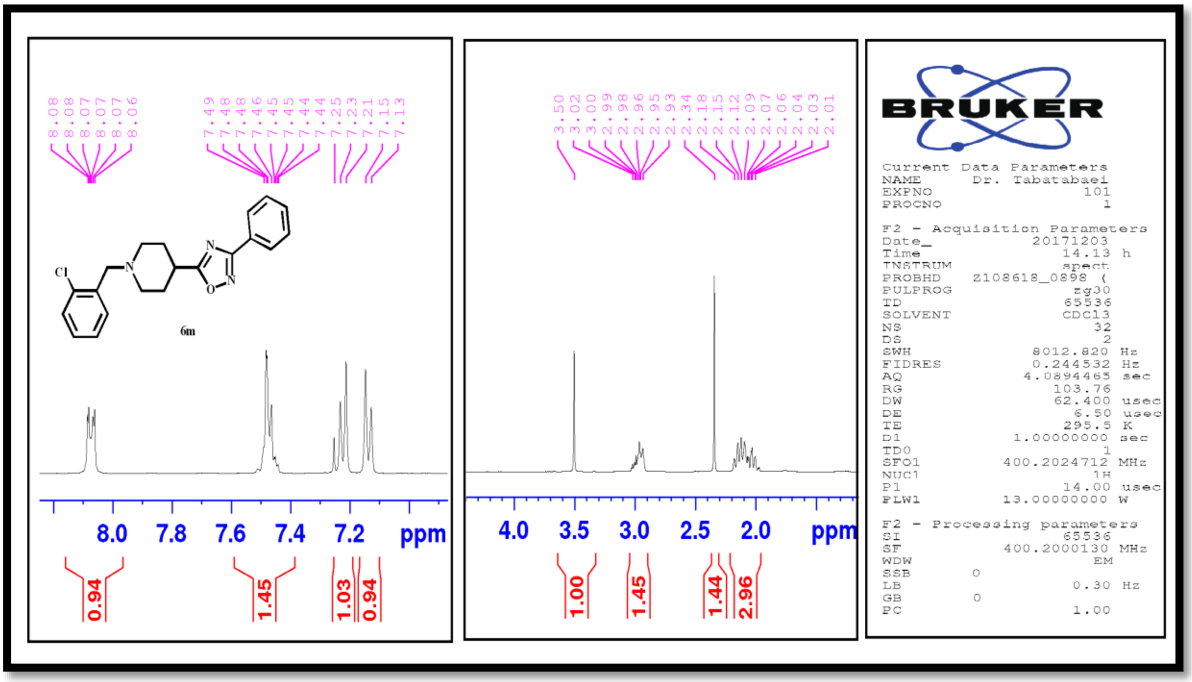

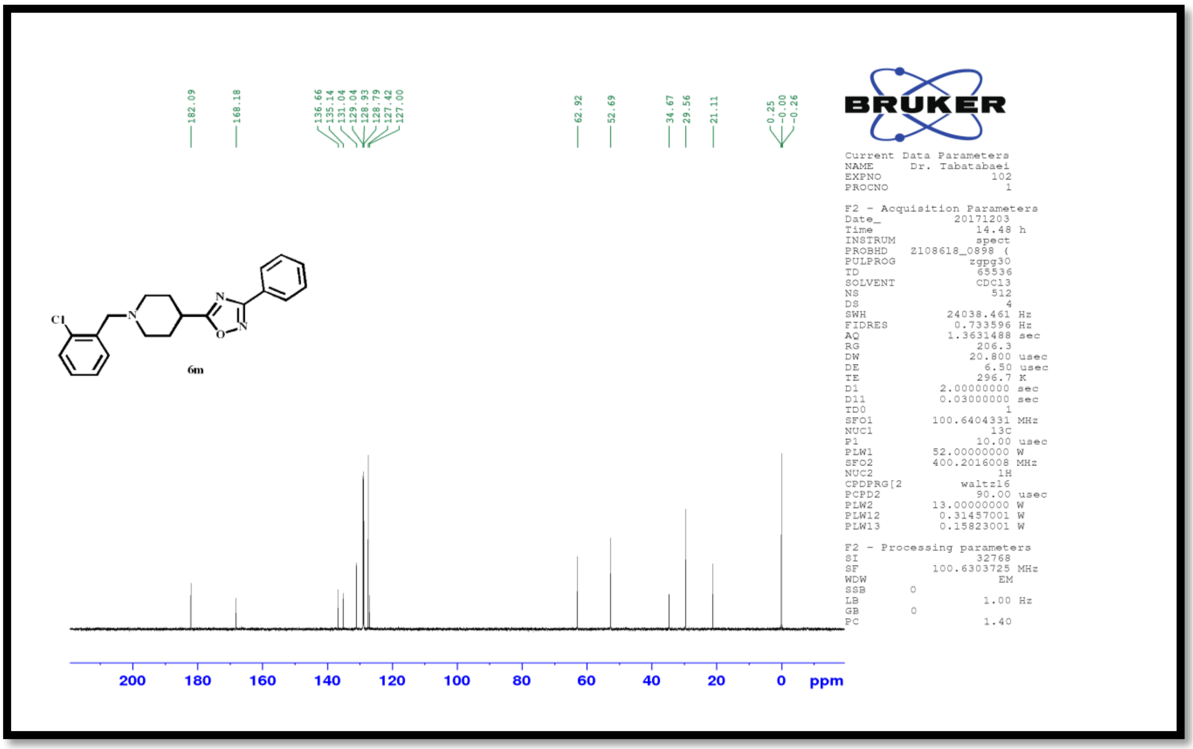

EXCLI Journal 2021;20:907-921 – ISSN 1611-2156
Received: February 27, 2021, accepted: May 10, 2021, published: May 18, 2021

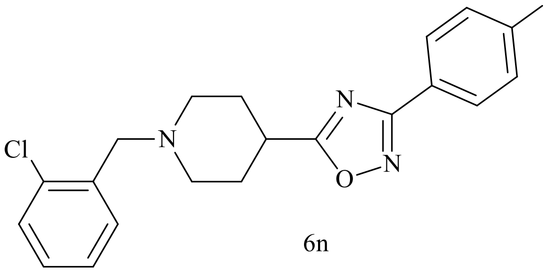

6n

5-(1-(2-chlorobenzyl)piperidin-4-yl)-3-(p-tolyl)-1,2,4-oxadiazole (6n)

White powder; yield: 33.8 %; mp: 121.8-124.7 °C; IR (KBr, cm^{-1}): 1582 (C=N), 1146 (C-O), 1442,1350 (CH_3); LC-MS $[M + 1]^+$: m/z 367.9; ^1H NMR ($CDCl_3$, 400 MHz) δ: 2.09-2.14 (m, 4H, H-piperidine), 2.24-2.31 (m, 2H, H-piperidine), 2.40 (s, 3H, CH_3), 2.97-3.04 (m, 3H, H-piperidine), 3.65 (s, 2H, CH_2-benzyl), 7.17-7.28 (m, 3H, H_4, H_5, H_6-benzyl), 7.34 (d, 2H, *J* = 8 Hz, H_3, H_5-phenyl), 7.50-7.51 (m, 1H, H_3-benzyl), 7.95 (d, 2H, *J* = 8 Hz, H_2, H_6-phenyl); ^{13}C NMR ($CDCl_3$, 100 MHz) δ: 21.56 (CH_3), 29.62 ($2CH_2$), 34.56 (CH), 52.85 ($2CH_2$), 59.34 (CH_2), 124.14 (C), 126.62 (2CH), 127.33 (CH), 128.07 (2CH), 129.51 (2CH), 130.48 (CH), 134.19 (C), 136.05 (C), 141.33 (C), 168.18 (C), 181.85 (C); Anal. calcd for $C_{21}H_{22}ClN_3O$: C, 68.56; H, 6.03; N, 11.42, found: C, 68.75; H, 5.99; N, 11.38.

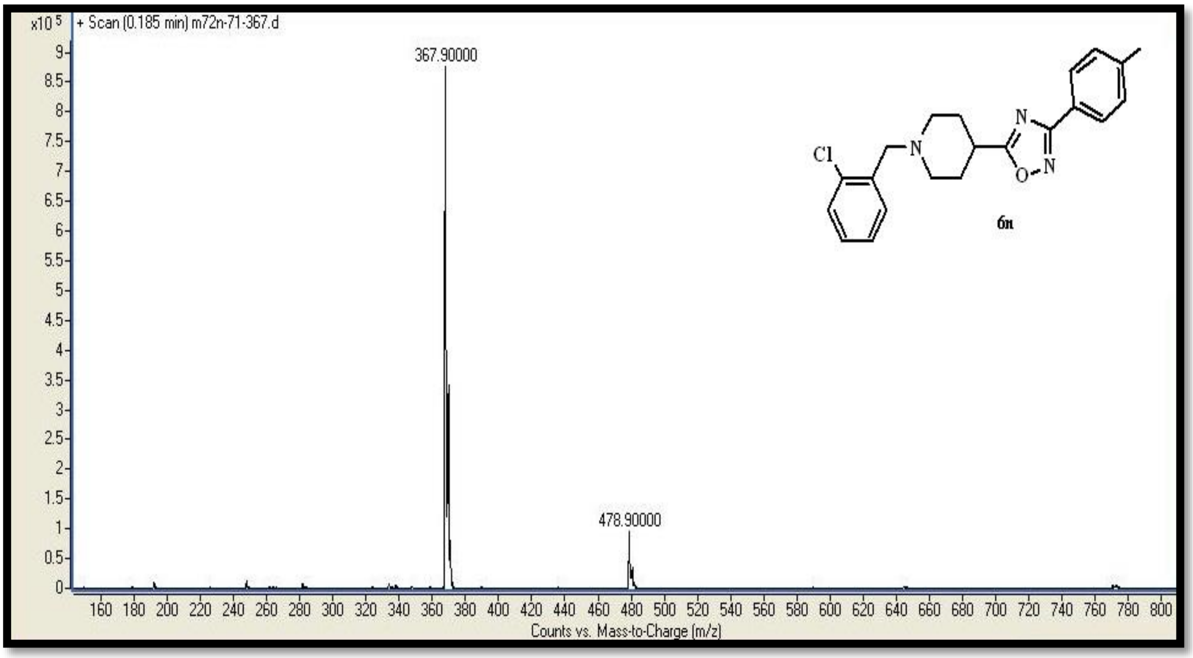
x10^5
+ Scan (0.185 min) m72n-71-367.d
367.90000
478.90000
Counts vs. Mass-to-Charge (m/z)
6n

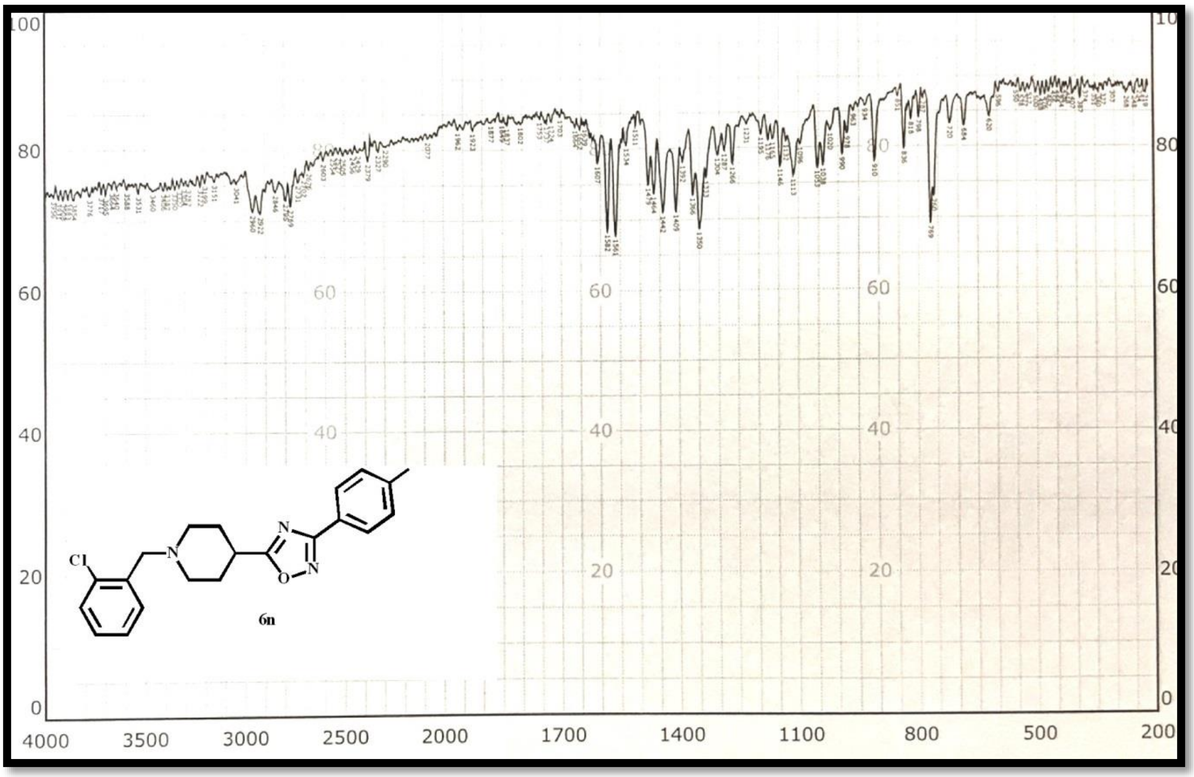
6n

EXCLI Journal 2021;20:907-921 – ISSN 1611-2156
Received: February 27, 2021, accepted: May 10, 2021, published: May 18, 2021

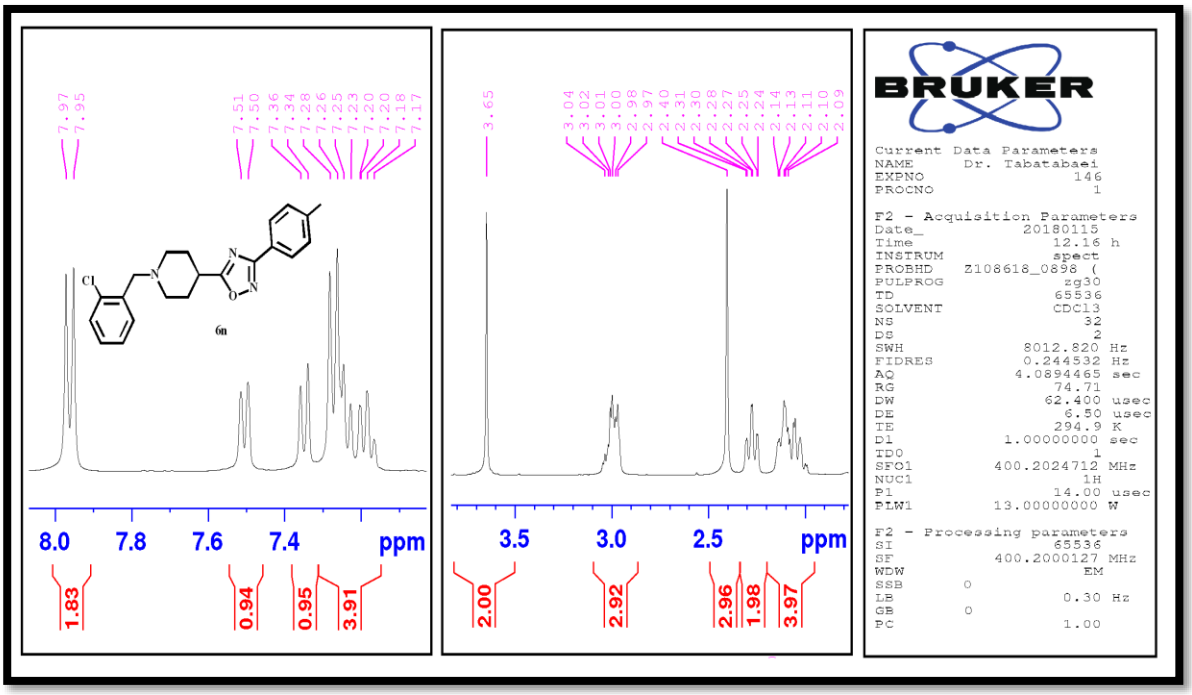

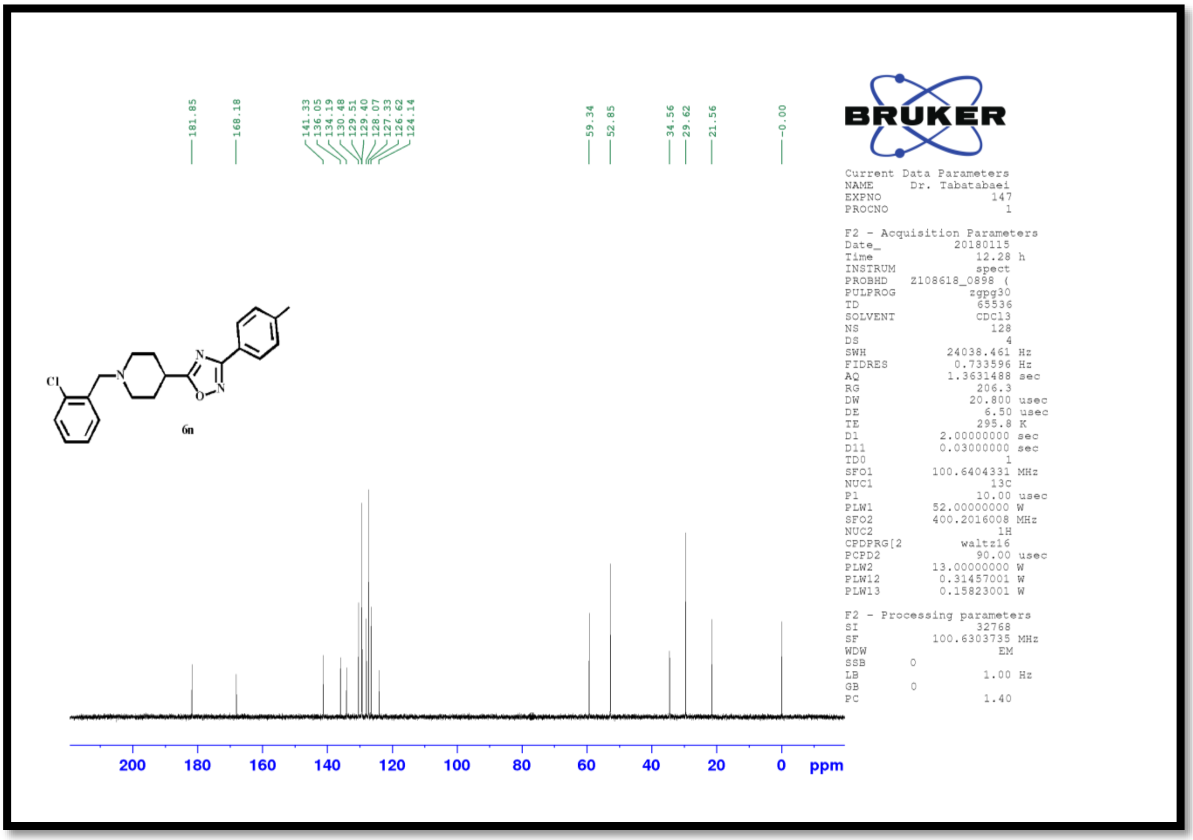

EXCLI Journal 2021;20:907-921 – ISSN 1611-2156
Received: February 27, 2021, accepted: May 10, 2021, published: May 18, 2021

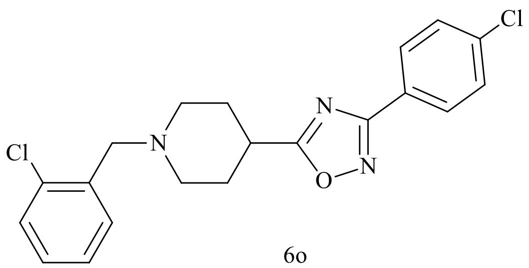

6o

5-(1-(2-chlorobenzyl)piperidin-4-yl)-3-(4-chlorophenyl)-1,2,4-oxadiazole (6o)

White powder; yield: 33.8 %; mp: 85.5-87 °C; IR (KBr, cm^{-1}): 1597 (C=N), 1141 (C-O); LC-MS $[M + 1]^+$: m/z 387.8; ^1H NMR ($CDCl_3$, 400 MHz) δ: 1.95-2.07 (m, 4H, H-piperidine), 2.19-2.24 (m, 2H, H-piperidine), 2.90-2.97 (m, 3H, H-piperidine), 3.58 (s, 2H, CH_2-benzyl), 7.14-7.19 (m, 2H, H_4, H_5-benzyl), 7.27 (d, 1H, *J* = 8 Hz ,H_3-benzyl), 7.36 (d, 2H, *J* = 8 Hz, H_3, H_5-phenyl), 7.42 (d, 1H, *J* = 8 Hz ,H_6-benzyl), 7.93 (d, 2H, *J* = 8 Hz, H_2, H_6-phenyl); ^{13}C NMR ($CDCl_3$, 100 MHz) δ: 29.60 ($2CH_2$), 34.55 (CH), 52.81 ($2CH_2$), 59.35 (CH_2), 125.50 (C), 126.67 (CH), 128.16 (2CH), 129.16 (2CH), 129.45 (2CH), 130.54 (CH), 134.25 (C), 135.99 (C), 137.21 (C), 167.43 (C), 182.28 (C); Anal. calcd for $C_{20}H_{19}Cl_2N_3O$: C, 61.86; H, 4.93; N, 10.82, found: C, 62.09; H, 4.92; N, 10.75.

EXCLI Journal 2021;20:907-921 – ISSN 1611-2156
Received: February 27, 2021, accepted: May 10, 2021, published: May 18, 2021

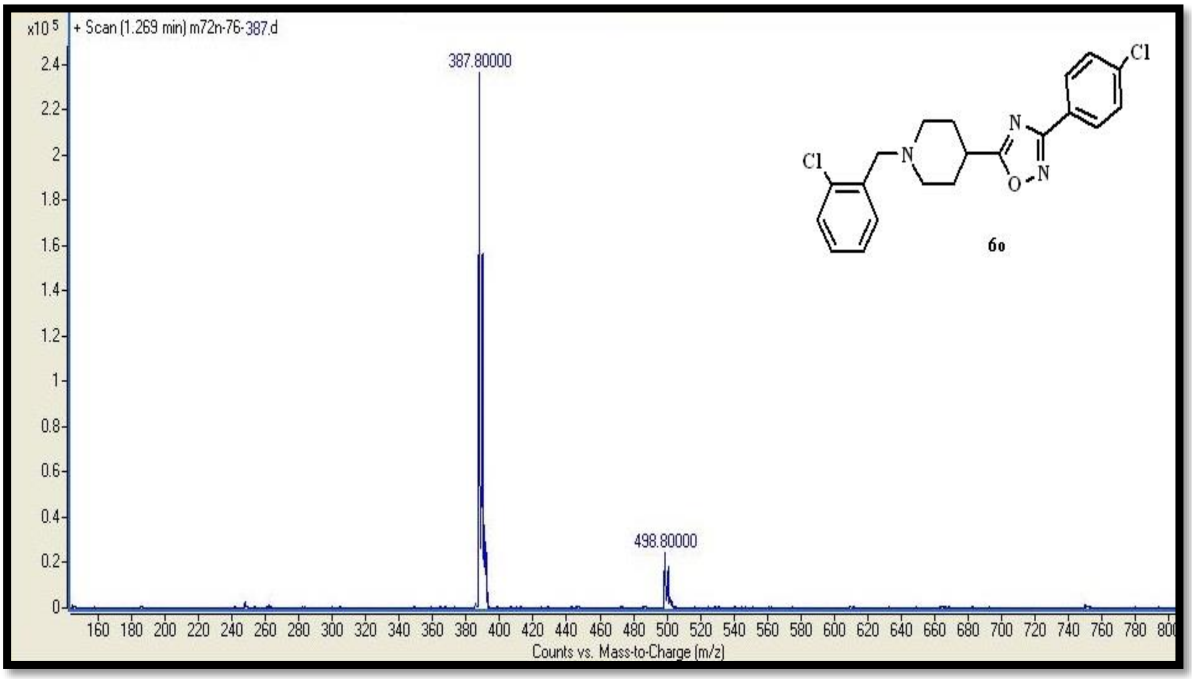

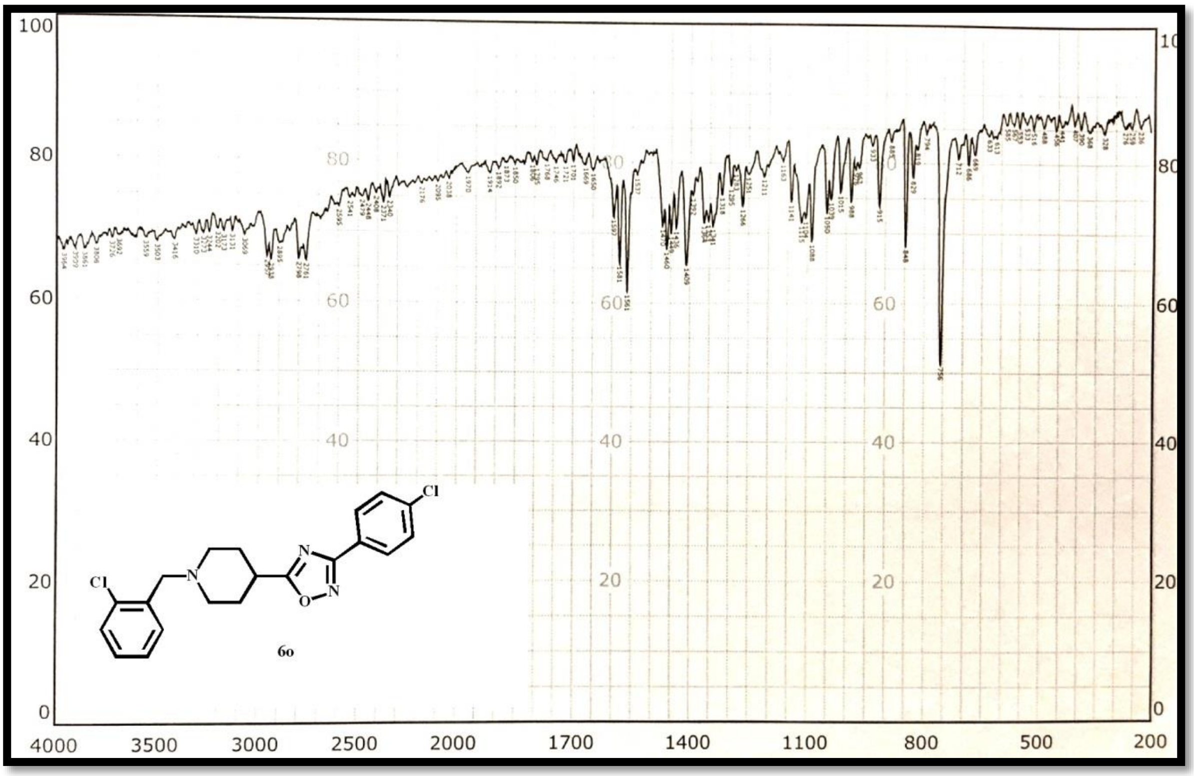

EXCLI Journal 2021;20:907-921 – ISSN 1611-2156
Received: February 27, 2021, accepted: May 10, 2021, published: May 18, 2021

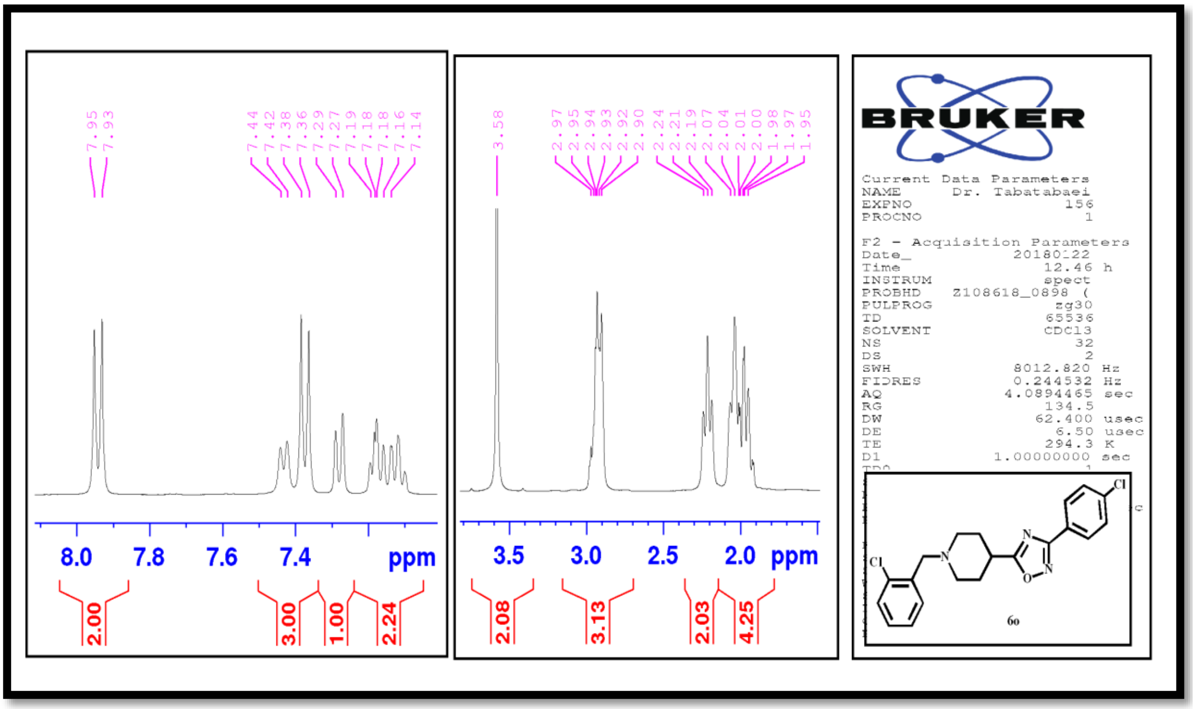

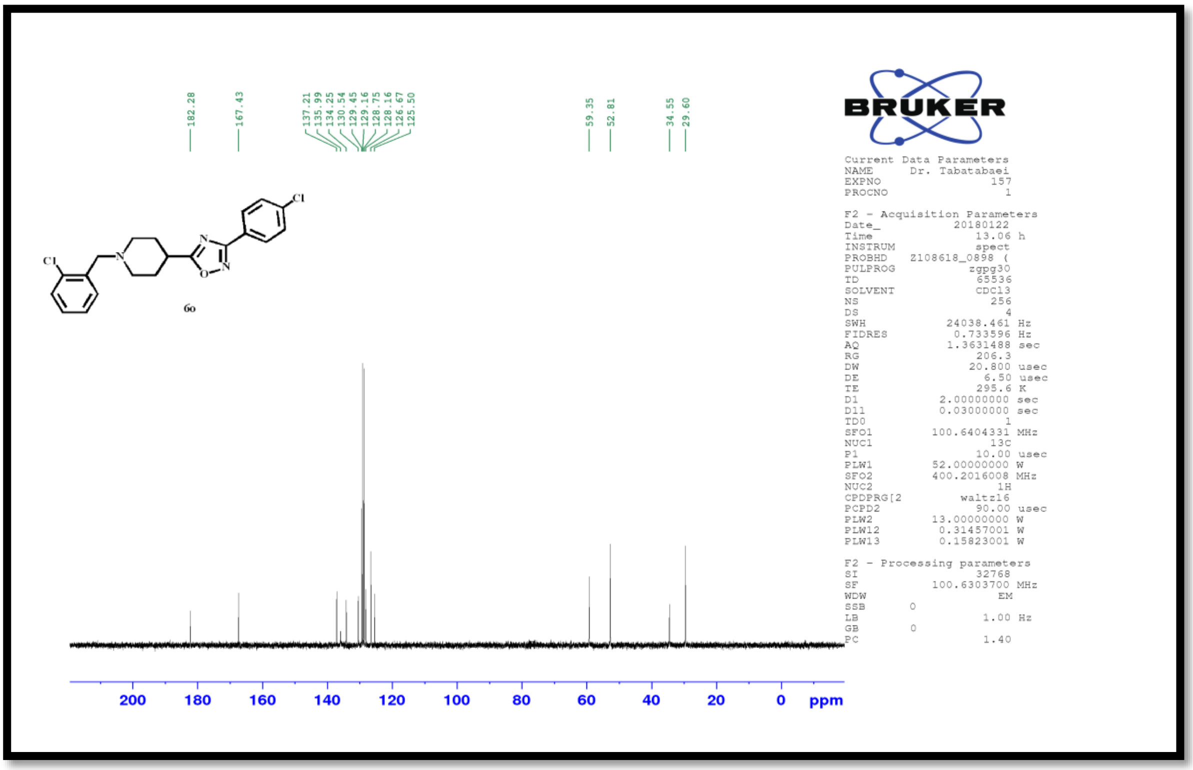

EXCLI Journal 2021;20:907-921 – ISSN 1611-2156
Received: February 27, 2021, accepted: May 10, 2021, published: May 18, 2021

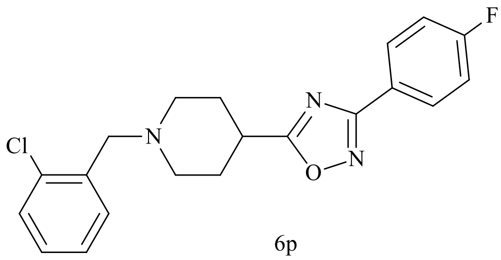

6p

5-(1-(2-chlorobenzyl)piperidin-4-yl)-3-(4-fluorophenyl)-1,2,4-oxadiazole (6p)

Light yellow powder; yield: 30.5 %; mp: 69.5-70 °C; IR (KBr, cm^{-1}): 1600 (C=N), 1212 (C-O); LC-MS $[M + 1]^+$: m/z 371.8; ^1H NMR ($CDCl_3$, 400 MHz) δ: 2.05-2.17 (m, 4H, H-piperidine), 2.29-2.34 (m, 2H, H-piperidine), 3.00-3.03 (m, 3H, H-piperidine), 3.68 (s, 2H, CH_2-benzyl), 7.20-7.29 (m, 3H, H_4, H_5, H_6-benzyl), 7.37 (d, 2H, *J* = 8 Hz, H_3, H_5-phenyl), 7.53-7.54 (m, 1H, H_3-benzyl), 8.08-8.12 (m, 2H, H_2, H_6-phenyl); ^{13}C NMR ($CDCl_3$, 100 MHz) δ: 29.61 ($2CH_2$), 34.54 (CH), 52.82 ($2CH_2$), 59.35 (CH_2), 115.90 (C), 123.22 (CH), 126.66 (2CH), 128.15 (2CH), 129.45 (2CH), 130.54 (CH), 134.24 (C), 136.00 (C), 163.27 (C), 167.41 (C), 182.18 (C); Anal. calcd for $C_{20}H_{19}ClFN_3O$: C, 64.60; H, 5.15; N, 11.30, found: C, 64.79; H, 5.13; N, 11.23.

EXCLI Journal 2021;20:907-921 – ISSN 1611-2156
Received: February 27, 2021, accepted: May 10, 2021, published: May 18, 2021

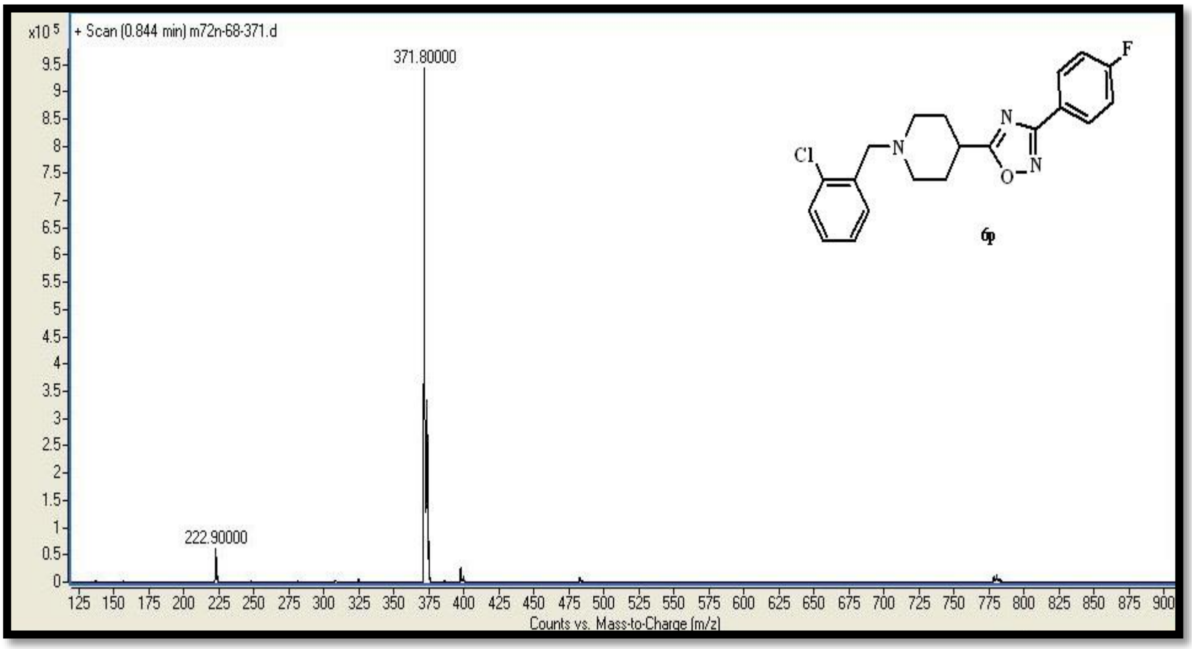

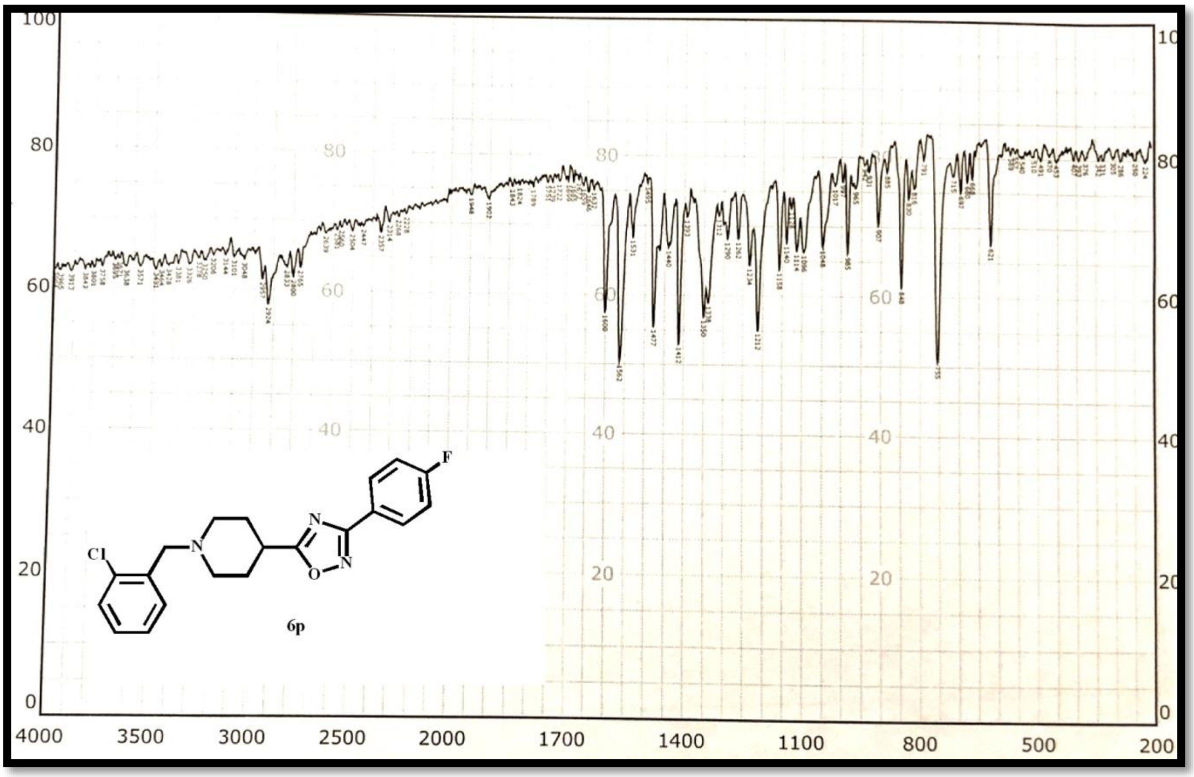

EXCLI Journal 2021;20:907-921 – ISSN 1611-2156
Received: February 27, 2021, accepted: May 10, 2021, published: May 18, 2021

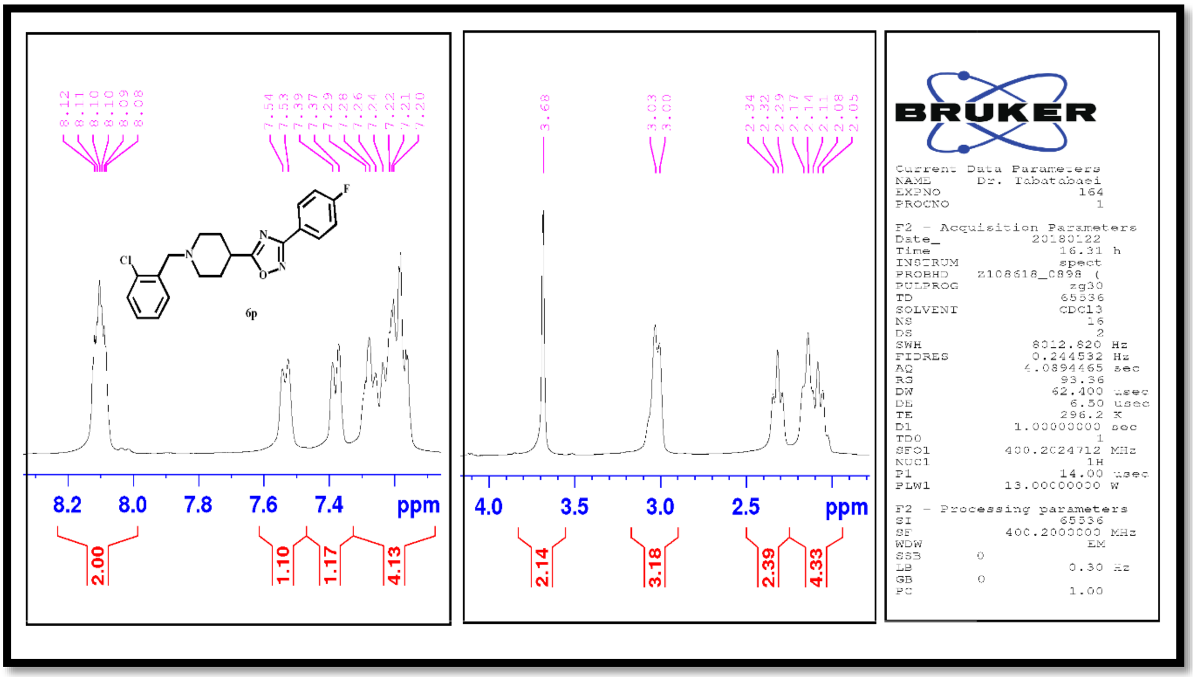

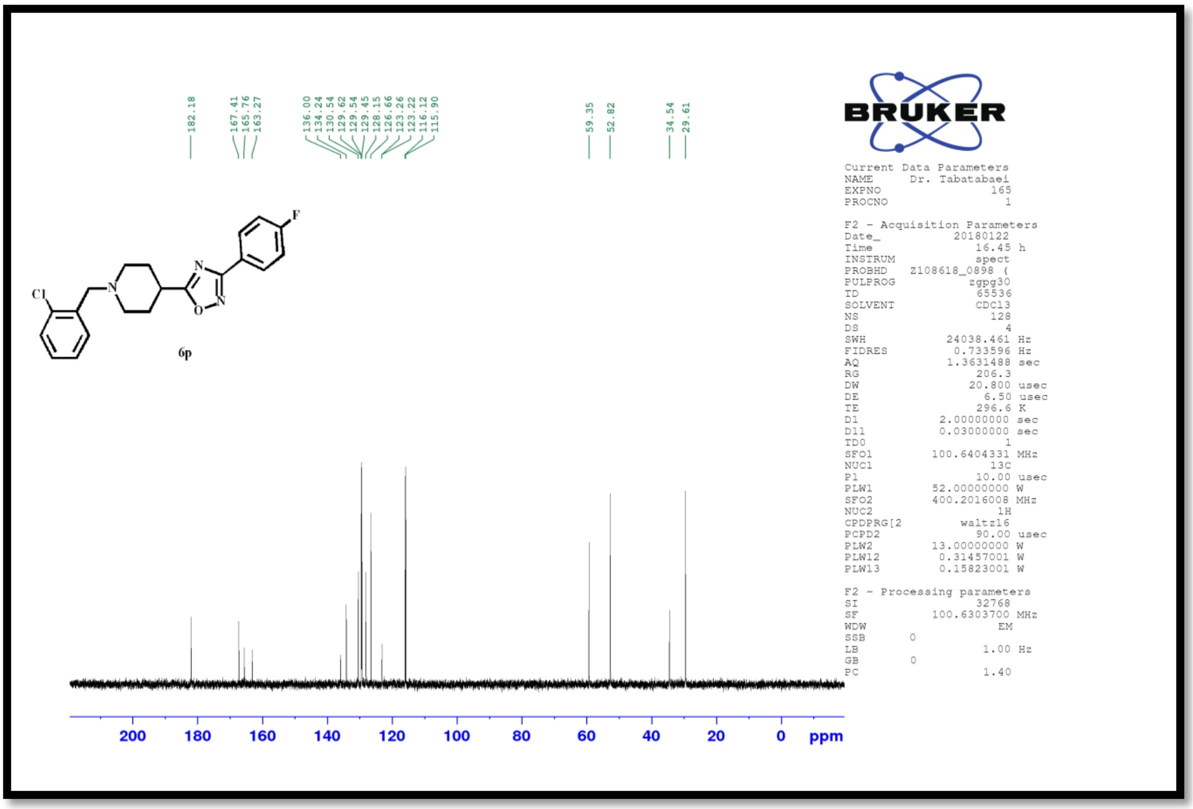

EXCLI Journal 2021;20:907-921 – ISSN 1611-2156
Received: February 27, 2021, accepted: May 10, 2021, published: May 18, 2021

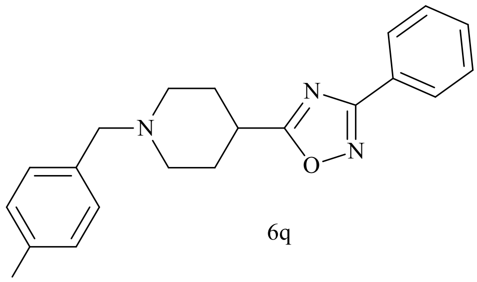

6q

5-(1-(4-methylbenzyl)piperidin-4-yl)-3-phenyl-1,2,4-oxadiazole (6q)

Light yellow powder; yield: 32.8 %; mp: 90.3-91.4 °C; IR (KBr, cm^{-1}): 1589 (C=N), 1145 (C-O), 1363,1440 (CH_3); LC-MS $[M + 1]^+$: m/z 334; ^1H NMR ($CDCl_3$, 400 MHz) δ: 2.09-2.14 (m, 6H, H-piperidine), 2.26-2.31 (m, 3H, CH_3), 2.98-3.05 (m, 3H, H-piperidine), 3.65 (s, 2H, CH_2-benzyl), 7.17-7.27 (m, 2H, H_3, H_5-benzyl), 7.34 (d, 2H, J = 8 Hz, H_2, H_6-benzyl),7.47-7.52 (m, 3H, H_3, H_4, H_5-phenyl), 8.07 (d, 2H, J = 8 Hz, H_2, H_6-phenyl); ^{13}C NMR ($CDCl_3$, 100 MHz) δ: 21.55 (CH_3), 29.62 ($2CH_2$), 34.56 (CH), 52.84 ($2CH_2$), 59.35 (CH_2), 126.63 (C), 127.42 (2CH), 128.80 (2CH), 129.41 (2CH), 130.50 (2CH), 131.06 (CH), 134.21 (C), 136.04 (C), 168.20 (C), 182.04 (C); Anal. calcd for $C_{21}H_{23}N_3O$: C, 75.65; H, 6.95; N, 12.60, found: C, 75.86; H, 6.94; N, 12.55.

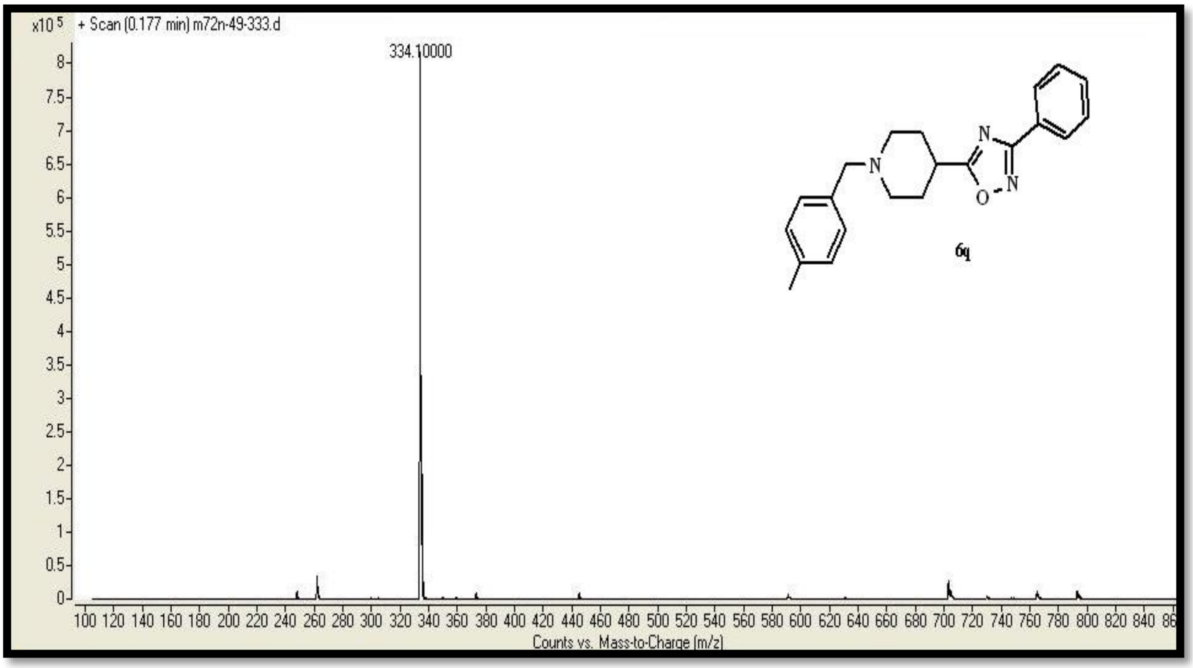
x10 5 + Scan (0.177 min) m72n-49-333.d
334.10000
Counts vs. Mass-to-Charge (m/z)
6q

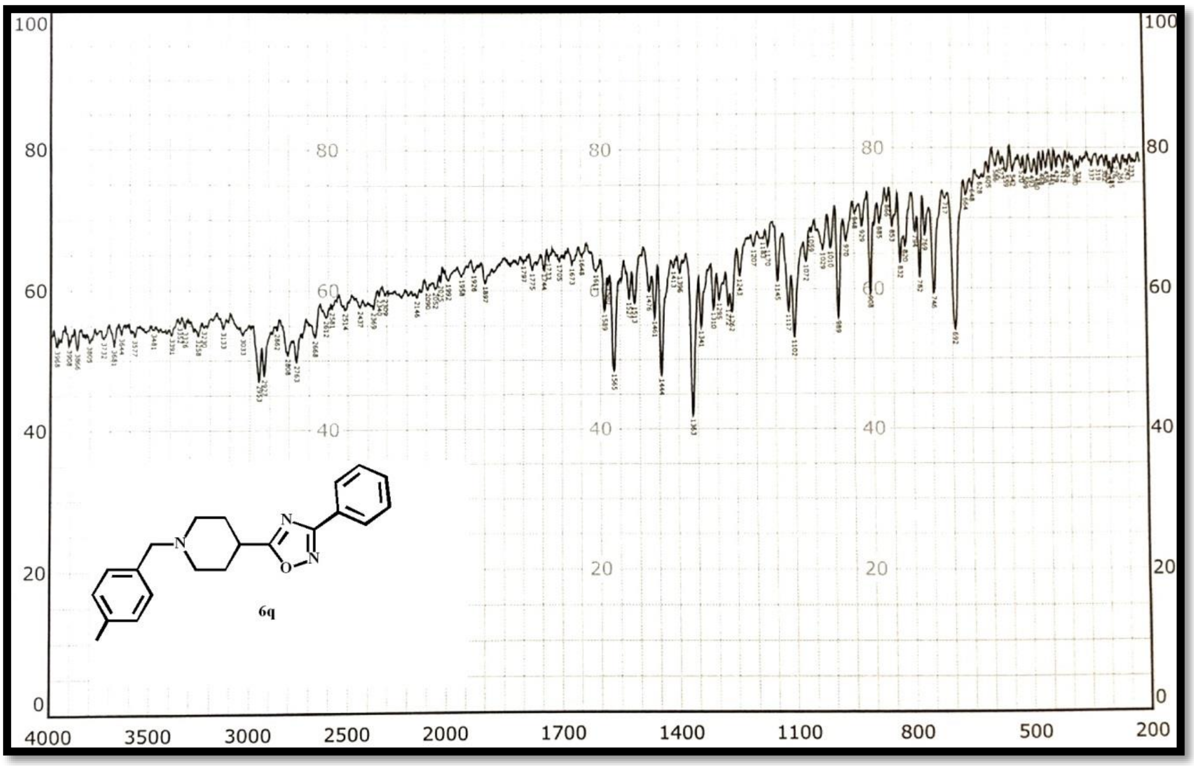
6q

EXCLI Journal 2021;20:907-921 – ISSN 1611-2156
Received: February 27, 2021, accepted: May 10, 2021, published: May 18, 2021

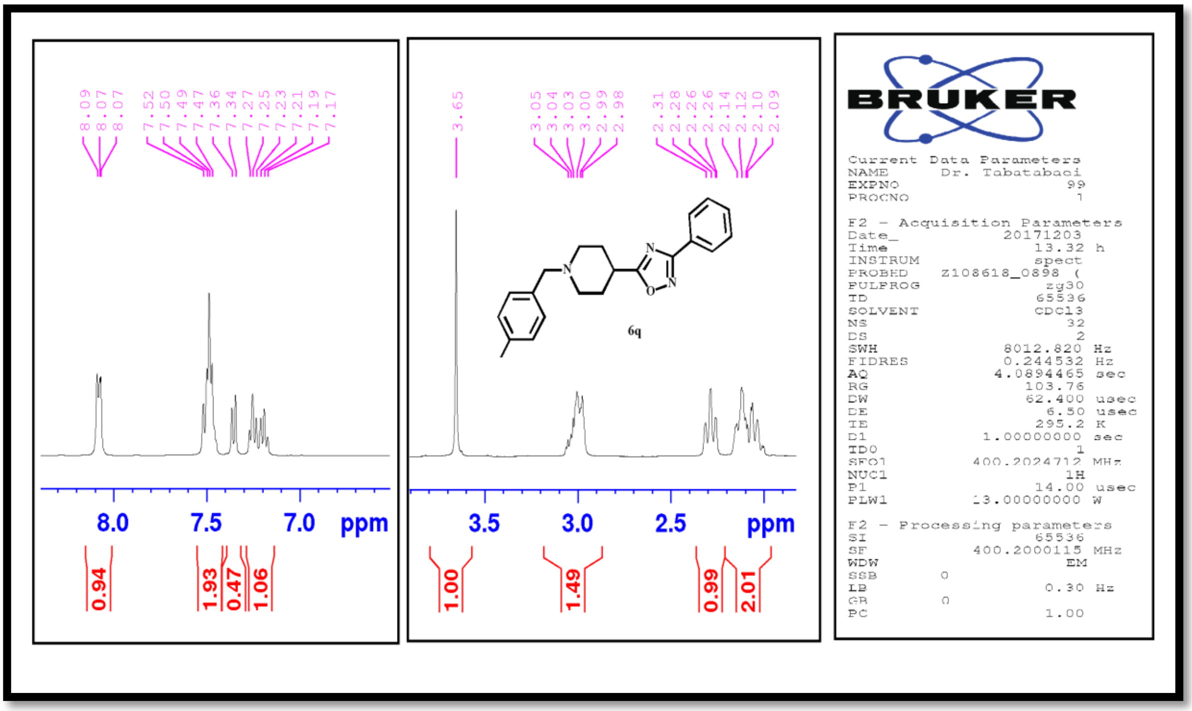

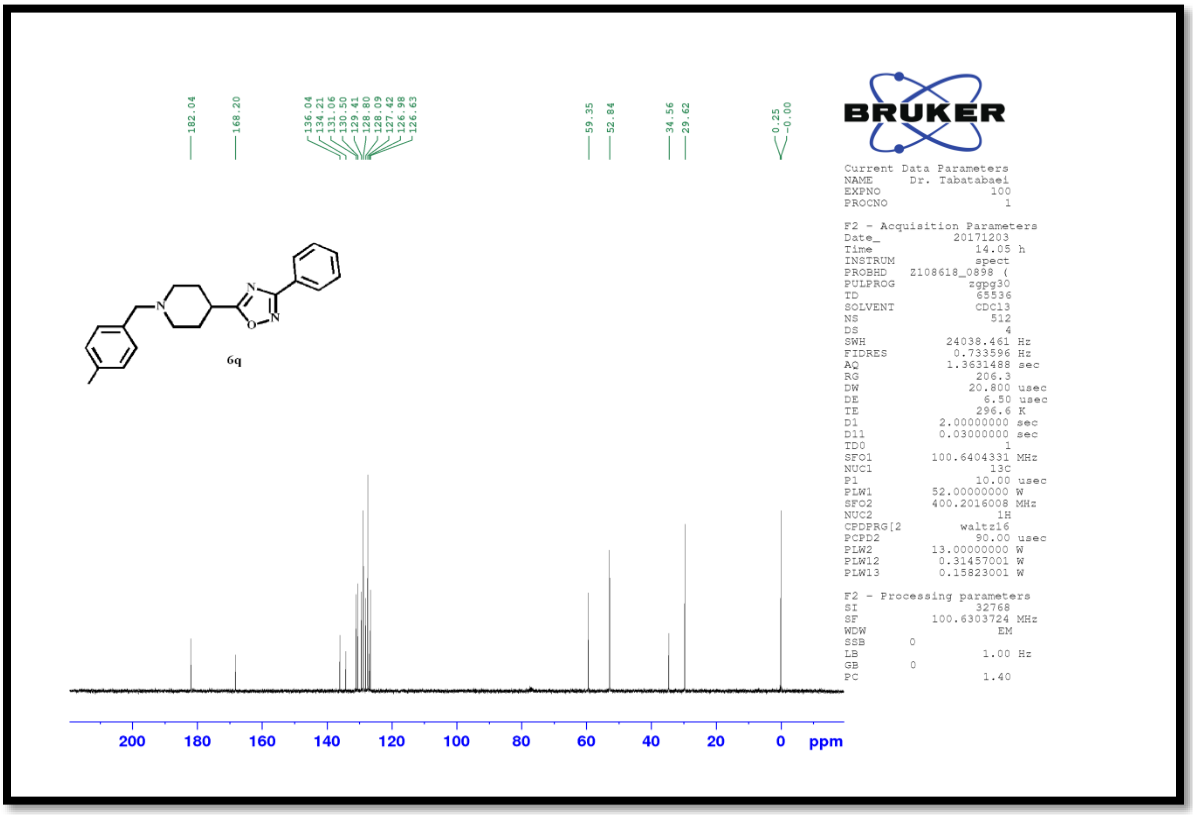

EXCLI Journal 2021;20:907-921 – ISSN 1611-2156
Received: February 27, 2021, accepted: May 10, 2021, published: May 18, 2021

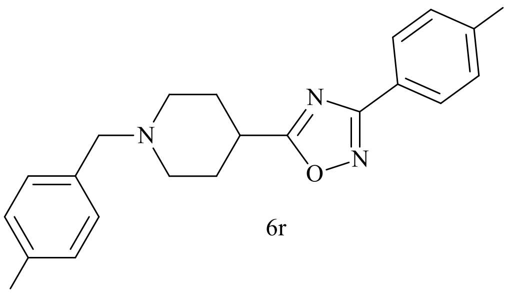

6r

5-(1-(4-methylbenzyl)piperidin-4-yl)-3-(p-tolyl)-1,2,4-oxadiazole (6r)

Light yellow powder; yield: 47.2 %; mp: 84.5-85.3 °C; IR (KBr, cm^{-1}): 1579 (C=N), 1117 (C-O), 1358,1410 (CH_3); LC-MS $[M + 1]^+$: m/z 347.9; ^1H NMR ($CDCl_3$, 400 MHz) δ: 2.02-2.15 (m, 6H, H-piperidine), 2.33 (s , 3H , CH_3-benzyl), 2.39 (s , 3H , CH_3-phenyl), 2.92-2.96 (m, 3H, H-piperidine), 3.49 (s, 2H, CH_2-benzyl), 7.12 (d, 2H, J = 8 Hz, H_3, H_5-benzyl), 7.20 (d, 2H, J = 8 Hz, H_2, H_6-benzyl), 7.25 (d, 2H, J = 8 Hz, H_3, H_5-phenyl), 7.95 (d, 2H, J = 8 Hz, H_2, H_6-phenyl); ^{13}C NMR ($CDCl_3$, 100 MHz) δ: 21.10 (CH_3), 21.54 (CH_3), 29.54 ($2CH_2$), 34.64 (CH), 52.68 ($2CH_2$), 62.90 (CH_2), 124.15 (C), 127.31 (2CH), 128.91 (2CH), 129.00 (2CH), 129.48 (2CH), 135.15 (C), 136.60 (C), 141.28 (C), 168.14 (C), 181.88 (C); Anal. calcd for $C_{22}H_{25}N_3O$: C, 76.05; H, 7.25; N, 12.09, found: C, 76.28; H, 7.23; N, 12.02.

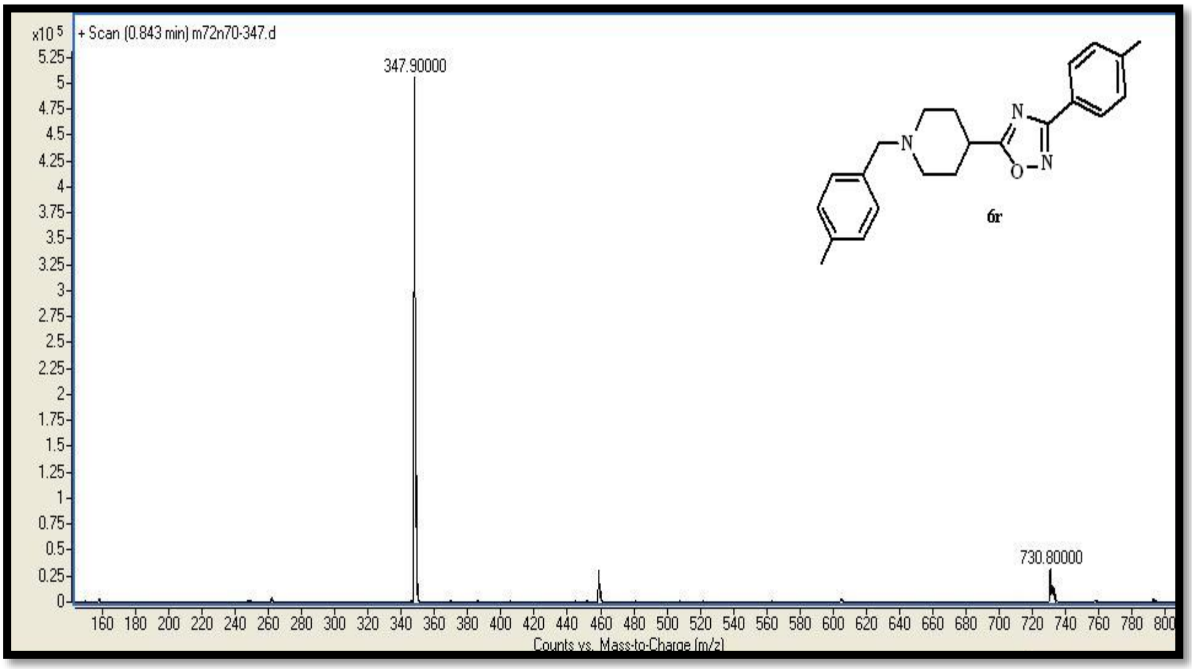
+ Scan (0.843 min) m72n70-347.d
347.90000
730.80000
Counts vs. Mass-to-Charge (m/z)
6r

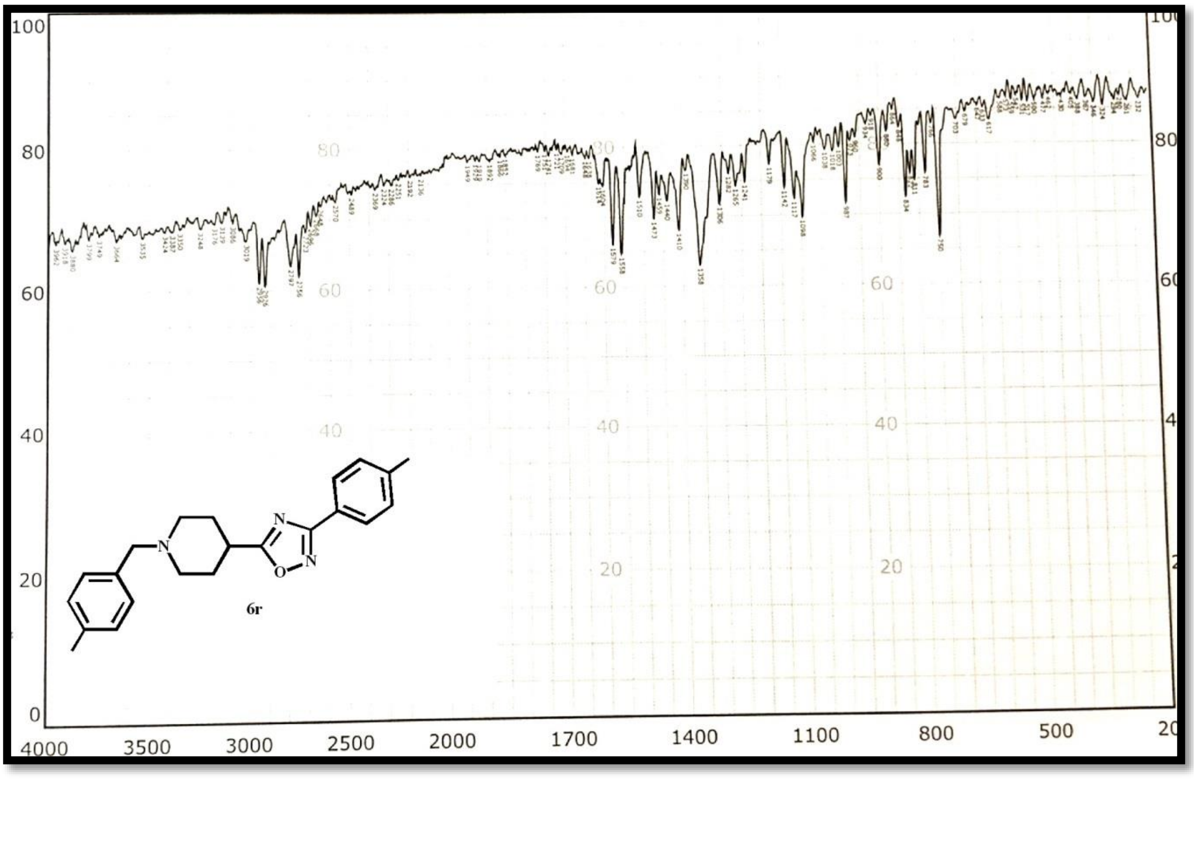
6r

EXCLI Journal 2021;20:907-921 – ISSN 1611-2156
Received: February 27, 2021, accepted: May 10, 2021, published: May 18, 2021

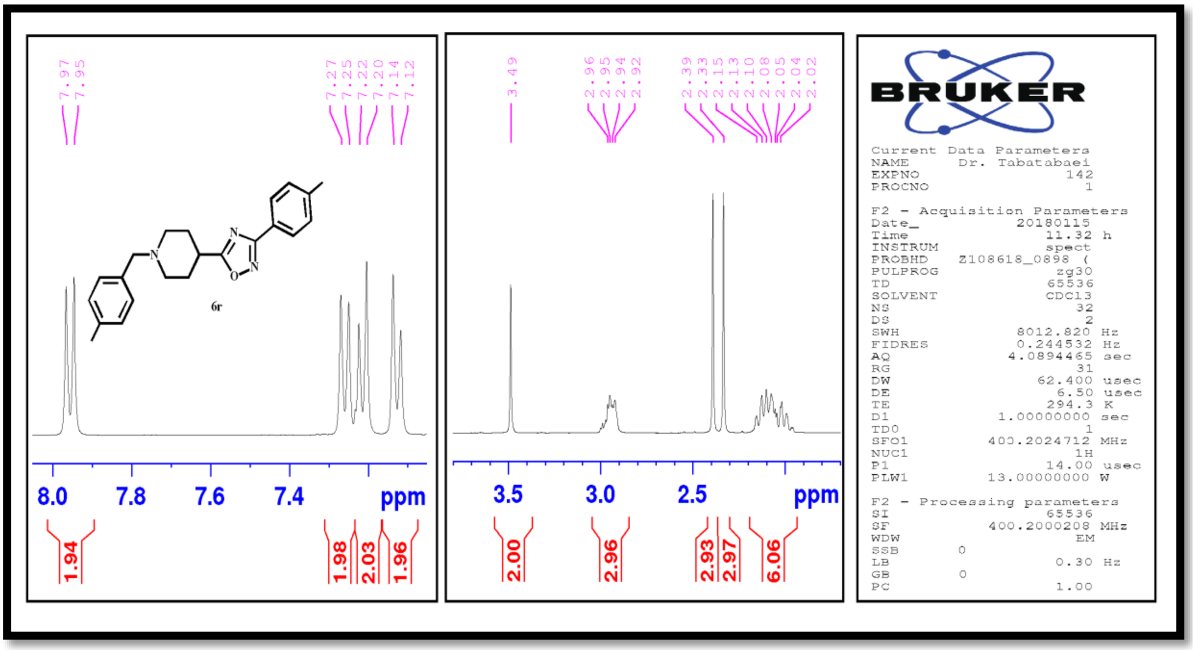

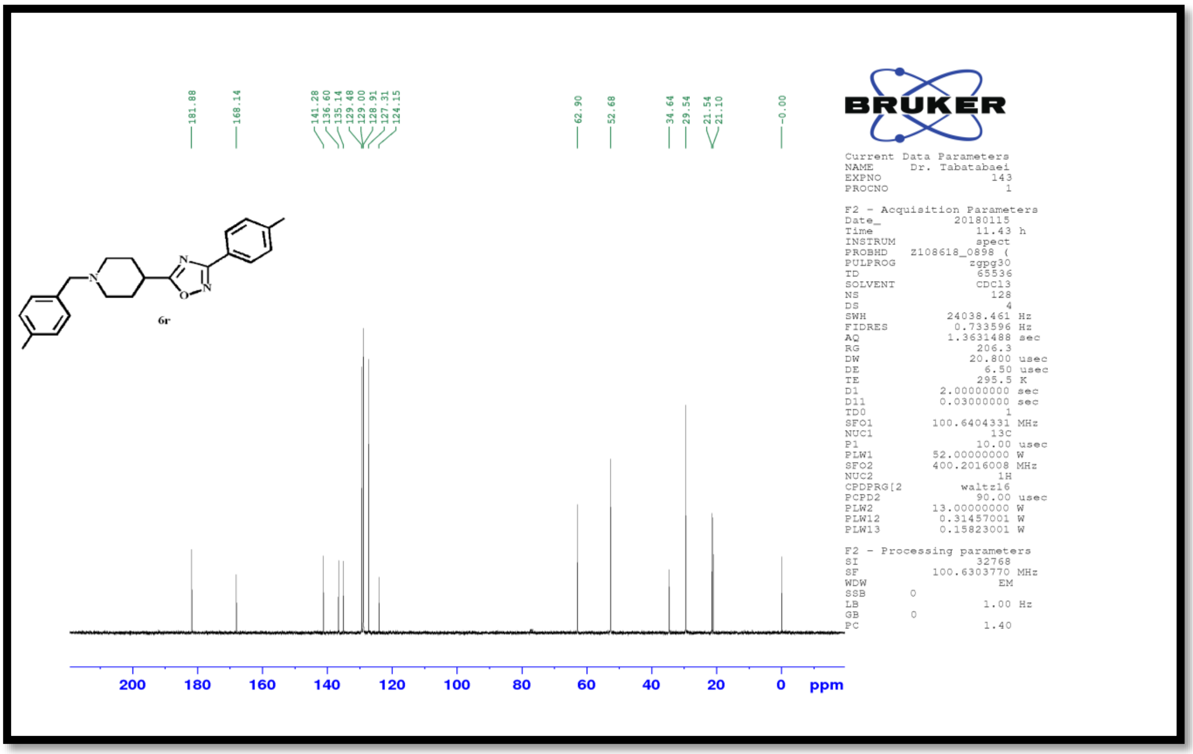

EXCLI Journal 2021;20:907-921 – ISSN 1611-2156
Received: February 27, 2021, accepted: May 10, 2021, published: May 18, 2021

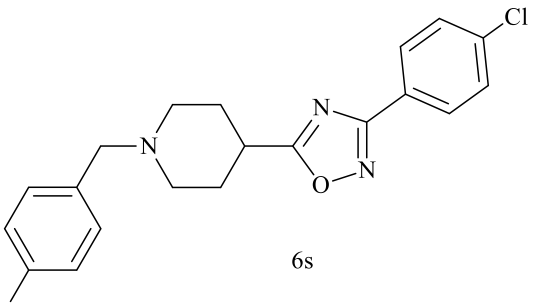

6s

3-(4-chlorophenyl)-5-(1-(4-methylbenzyl)piperidin-4-yl)-1,2,4-oxadiazole (6s)

Light yellow powder; yield: 47.3 %; mp: 111.4-112.5 °C; IR (KBr, cm^{-1}): 1585 (C=N), 1128 (C-O), 1361,1446 (CH_3); LC-MS $[M + 1]^+$: m/z 367.8; ^1H NMR ($CDCl_3$, 400 MHz) δ: 1.95-2.10 (m, 6H, H-piperidine), 2.26 (s, 3H, CH_3), 2.86-2.91 (m, 3H, H-piperidine), 3.43 (s, 2H, CH_2-benzyl), 7.05 (d, 2H, J = 8 Hz, H_3, H_5-benzyl), 7.15 (d, 2H, J = 8 Hz, H_2, H_6-benzyl), 7.35 (d, 2H, J = 8 Hz, H_3, H_5-phenyl), 7.92 (d, 2H, J = 8 Hz, H_2, H_6-phenyl); ^{13}C NMR ($CDCl_3$, 100 MHz) δ: 21.15 (CH_3), 29.55 ($2CH_2$), 34.66 (CH), 52.68 ($2CH_2$), 62.93 (CH_2), 125.53 (C), 128.76 (2CH), 128.97 (2CH), 129.07 (2CH), 129.15 (2CH), 135.09 (C), 136.72 (C), 137.19 (C), 167.42 (C), 182.35 (C); Anal. calcd for $C_{21}H_{22}ClN_3O$: C, 68.56; H, 6.03; N, 11.42, found: C, 68.79; H, 6.01; N, 11.38.

EXCLI Journal 2021;20:907-921 – ISSN 1611-2156
Received: February 27, 2021, accepted: May 10, 2021, published: May 18, 2021

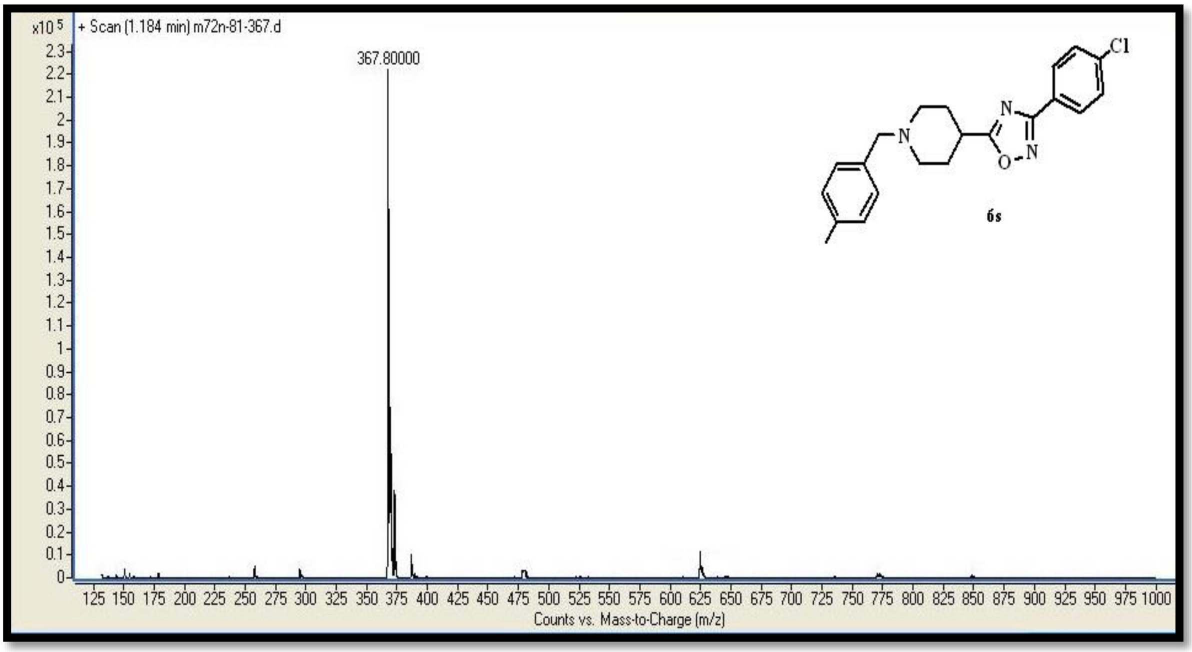

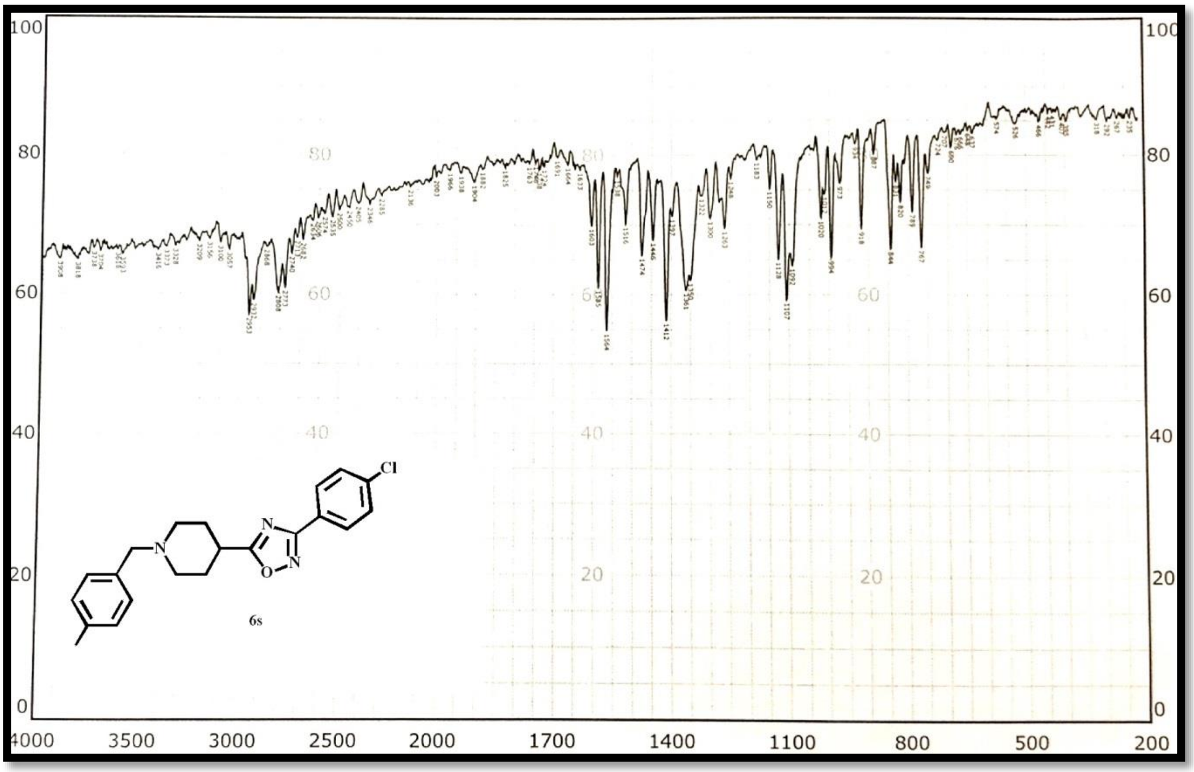

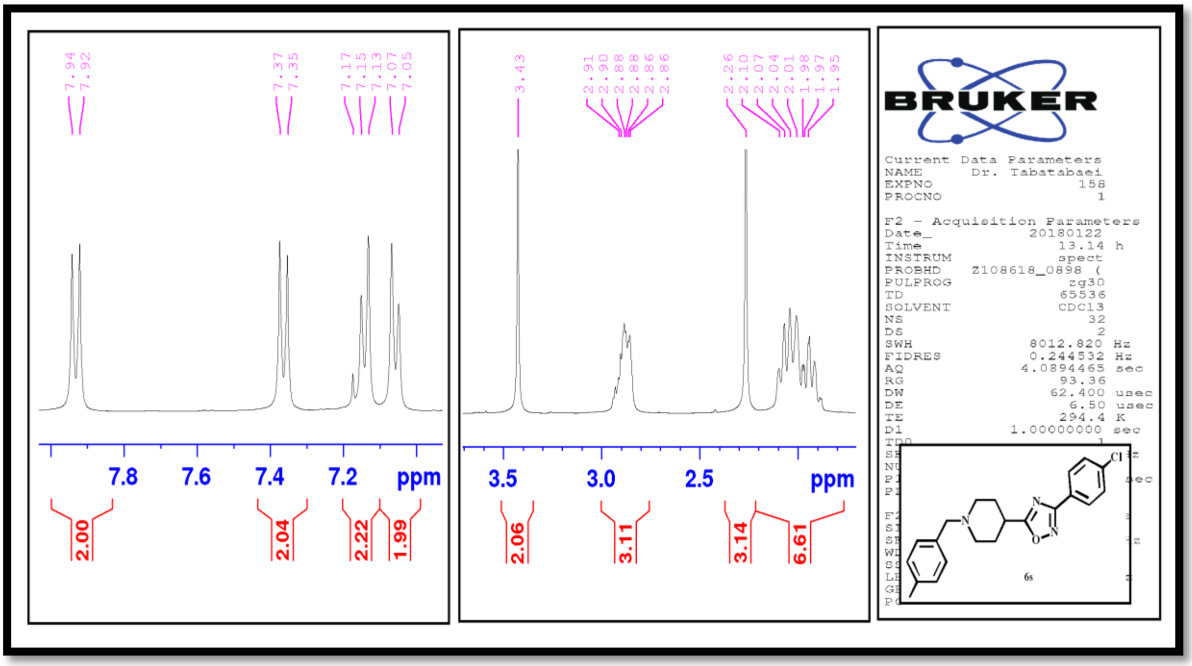

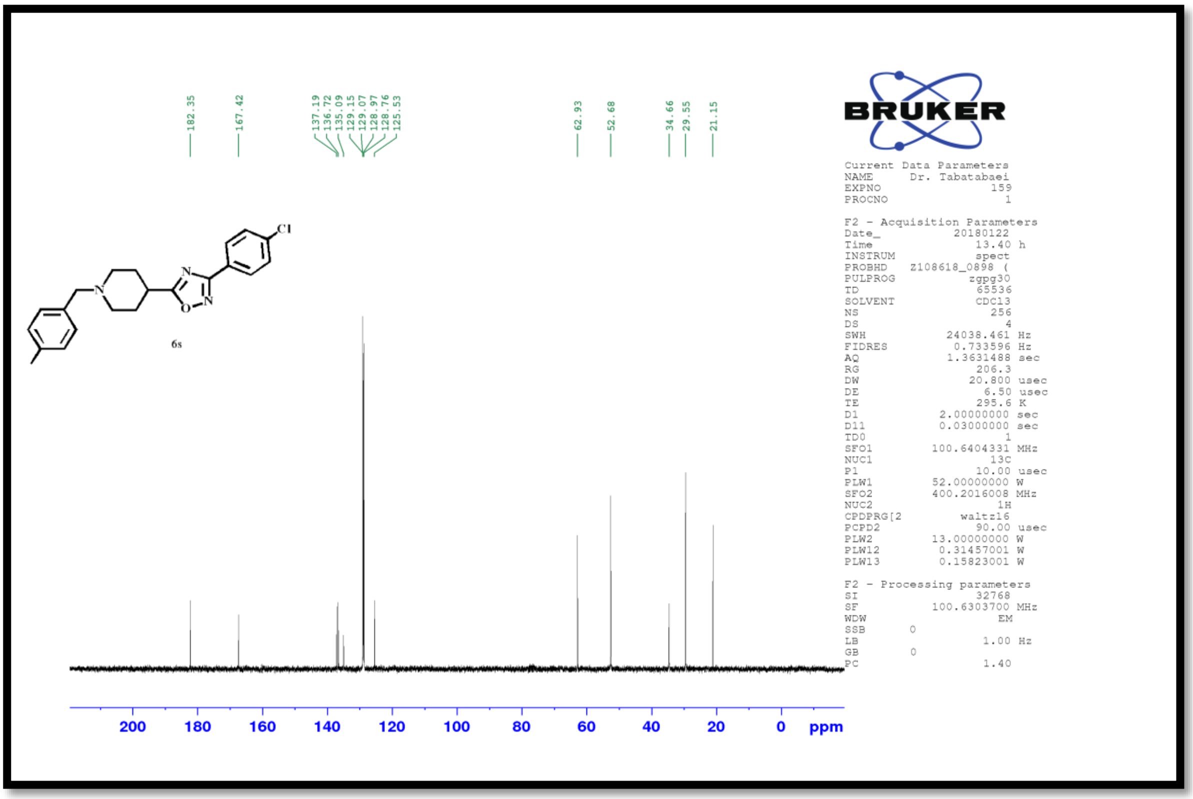

EXCLI Journal 2021;20:907-921 – ISSN 1611-2156
Received: February 27, 2021, accepted: May 10, 2021, published: May 18, 2021

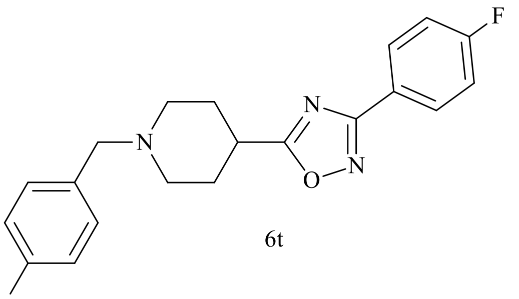

6t

3-(4-fluorophenyl)-5-(1-(4-methylbenzyl)piperidin-4-yl)-1,2,4-oxadiazole (6t)

Light yellow powder; yield: 48.2 %; mp: 96.5-97.2 °C; IR (KBr, cm^{-1}): 1600 (C=N), 1223 (C-O), 1352,1444 (CH_3); LC-MS $[M + 1]^+$: m/z 351.7; ^{1}H NMR ($CDCl_3$, 400 MHz) δ: 1.99-2.17 (m, 6H, H-piperidine), 2.34 (s, 3H, CH_3), 2.94-2.98 (m, 3H, H-piperidine), 3.50 (s, 2H, CH_2-benzyl), 7.13-7.17 (m, 4H, H_2, H_3, H_5, H_6-benzyl), 7.23 (t, 2H, J = 8 Hz, H_3, H_5-phenyl), 8.05-8.09 (m, 2H, H_2, H_6-phenyl); ^{13}C NMR ($CDCl_3$, 100 MHz) δ: 21.11 (CH_3), 29.54 ($2CH_2$), 34.64 (CH), 52.67 ($2CH_2$), 62.92 (CH_2), 115.86 (2CH), 123.20 (C), 128.93 (2CH), 129.51 (2CH), 135.09 (2CH), 136.68 (C), 163.23 (C), 165.72 (C), 167.36 (C), 182.22 (C); Anal. calcd for $C_{21}H_{22}FN_3O$: C, 71.77; H, 6.31; N, 11.96, found: C, 72.02; H, 6.30; N, 11.87.

EXCLI Journal 2021;20:907-921 – ISSN 1611-2156
Received: February 27, 2021, accepted: May 10, 2021, published: May 18, 2021

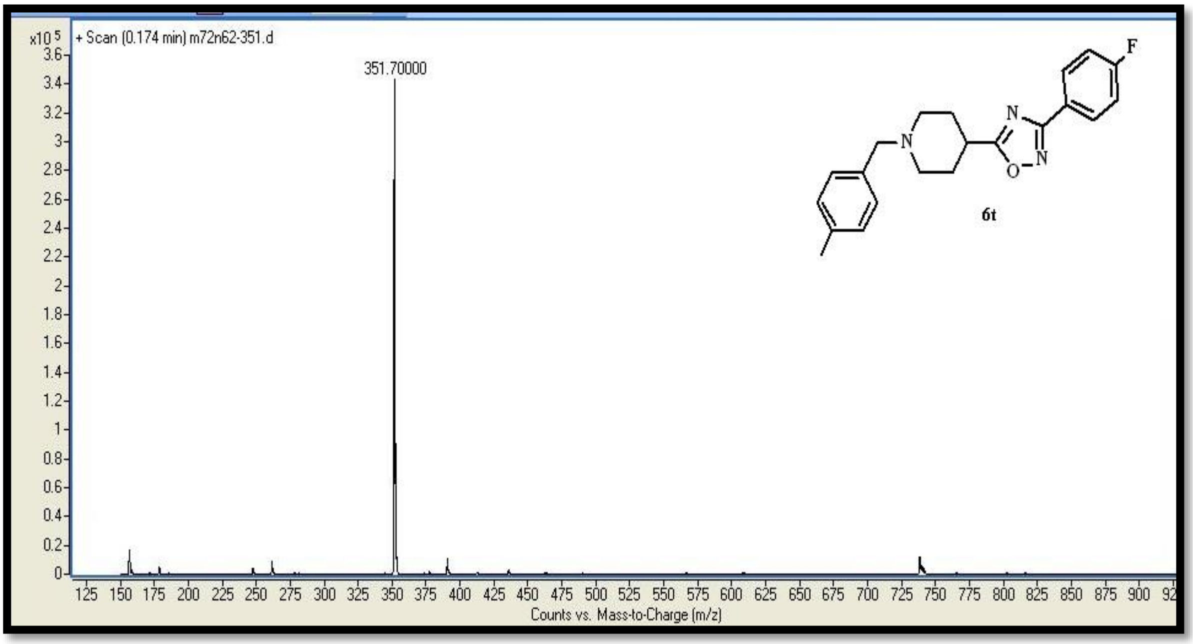

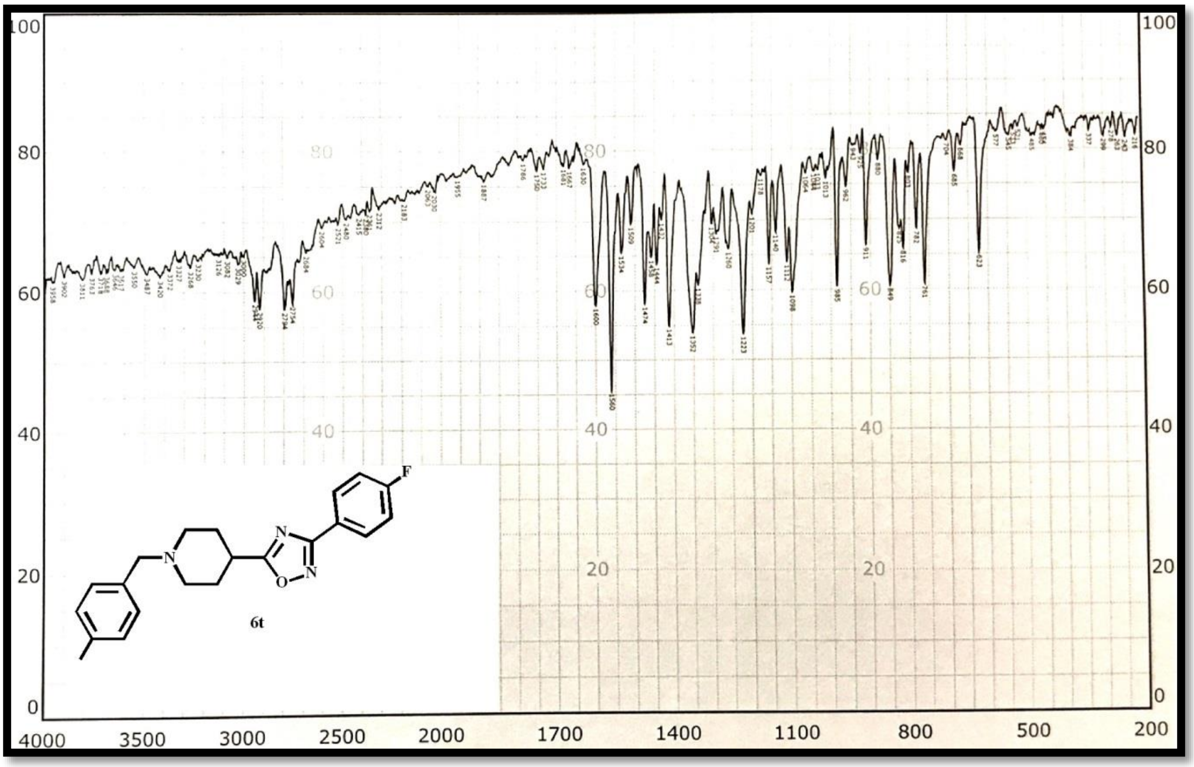

EXCLI Journal 2021;20:907-921 – ISSN 1611-2156
Received: February 27, 2021, accepted: May 10, 2021, published: May 18, 2021

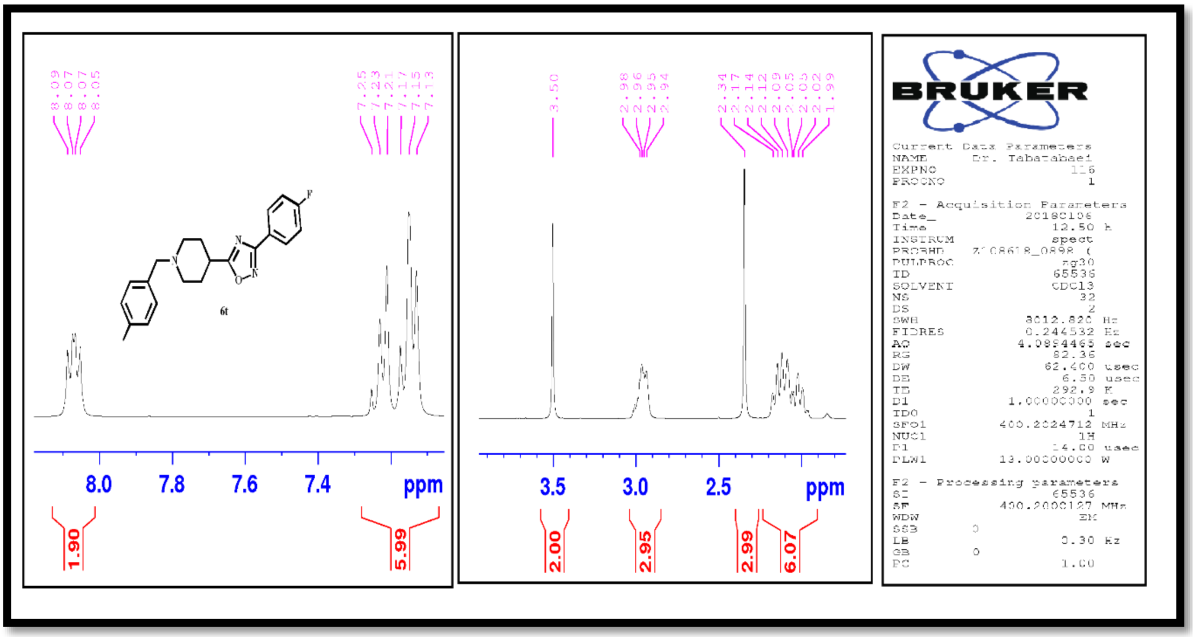

Supplement: Supplementary information [file EXCLI-20-907-s-001.pdf]
